# Adaptive shifts in amygdala–hippocampal theta coupling govern aversive learning and extinction

**DOI:** 10.1038/s41467-026-73967-4

**Published:** 2026-06-10

**Authors:** Saurabh Sonkusare, Qiong Ding, Christopher Weirich, Yashu Feng, Wei Liu, Ruoqi Yang, Alekhya Mandali, Samantha Sallie, Violeta Casero, Chunyan Cao, Dianyou Li, Bomin Sun, Shikun Zhan, Valerie Voon

**Affiliations:** 1 Institute of Science and Technology for Brain-Inspired Intelligence, Fudan University, Shanghai, China; 2 Department of Psychiatry, University of Cambridge, Cambridge, UK; 3 Department of Neurosurgery, Centre for Functional Neurosurgery, Ruijin Hospital, Shanghai Jiao Tong University School of Medicine, Shanghai, China; 4 School of Psychology, University of Sheffield, Sheffield, UK

**Keywords:** Fear conditioning, Extinction, Hippocampus, Amygdala

## Abstract

Adaptive behaviour relies on the flexible encoding and suppression of aversive associations often underpinned by amygdala-hippocampal interactions. Yet the spectral and directional dynamics underlying these interactions in humans remain poorly understood. Using intracranial EEG recordings from the amygdala and the hippocampus acquired during a two-day aversive learning and extinction task, we identified frequency-specific shifts: amygdala theta (3–8 Hz) and gamma (30–45 Hz) power increased during conditioning and decreased during extinction, while hippocampal alpha and gamma activity gave way to theta and gamma during extinction. Directional phase connectivity, results showed frequency-specific reversals: amygdala-to-hippocampus dominance at 3-5 Hz and hippocampus-to-amygdala predominance at 6-8 Hz, a reconfiguration validated by computational modelling. These findings uncover distinct theta sub-bands coordinating dynamic, bidirectional communication in the human amygdala–hippocampal circuit, elucidating a neural mechanism for the flexible regulation of emotional memory.

## Abstract (web summary)

How the human amygdala and hippocampus coordinate the learning and suppression of aversive associations has remained unclear. Here, human intracranial recordings reveal that the direction of communication reverses in distinct theta sub-bands between these regions across learning and extinction.

## Introduction

Organisms form associations between environmental stimuli and outcomes, a process critical for adaptive behaviour and survival. Aversive conditioning—where a neutral stimulus acquires negative valence through pairing with an aversive event, and its subsequent suppression via extinction learning—form the cornerstone of emotional memory regulation^1,2^. These processes support not only fundamental to learning but also central to understanding maladaptive responses in psychiatric disorders such as anxiety and post-traumatic stress disorder (PTSD)^3,4^.

The conditioning-extinction paradigm, rooted in Pavlovian learning principles, provides a widely used framework to investigate these associative processes across species^5,6^. In animal models, aversive conditioning is typically induced using unconditioned stimuli (US) such as foot shocks or loud auditory stimuli, while human studies often employ painful shocks, airpuffs, or visual/auditory stimuli^5,7^. However, the salience of aversive US can confound interpretations of learning effects, as the observed responses may reflect general arousal rather than specific associative learning^8^. Moreover, extinction is often interpreted as the weakening of conditioned responses, but it may instead involve the formation of new inhibitory memories that compete with the original association, complicating the distinction between unlearning and response suppression^9,10^. These challenges are further compounded in real-world scenarios, where fear memories are often embedded in complex networks involving multiple stimuli and contextual cues^3,11^.

Nevertheless, the conditioning-extinction paradigm has revealed a distributed network supporting aversive learning and extinction, prominently involving the amygdala, hippocampus, insula, anterior cingulate cortex (ACC), and prefrontal cortex^12–14^. Within this network, the amygdala and hippocampus are central to the neural circuits governing aversive learning and extinction. The amygdala encodes emotional salience and drives fear responses, while the hippocampus supports contextual memory and the flexible regulation of conditioned associations^7,15^. Although these regions interact dynamically, the neural mechanisms underlying their coordination during aversive learning and extinction remain incompletely understood in humans, particularly at the level of fast-timescale neuronal oscillations.

Oscillatory activity in the theta band (3–8 Hz) has emerged as a key candidate mediating amygdala-hippocampal interactions, with distinct sub-bands within the theta range potentially supporting different phases of learning^16^. For instance, rodent studies have identified a frequency-specific dissociation, wherein lower theta frequencies (<6 Hz) dominate during aversive learning, while higher theta (>6 Hz) frequencies are associated with extinction and safety learning^17^. These findings suggest a frequency-specific reconfiguration of network dynamics that tracks the transition from threat acquisition to safety learning. Additionally, functional heterogeneity within theta band, is supported by animal studies^18^ and converging human EEG, including iEEG evidence demonstrating dissociable roles of low (3-6 Hz)- and high-theta activity (>6 Hz)^19–21^. However, direct evidence for such frequency-resolved mechanisms in human amygdala-hippocampal coupling is limited and lacking in learning paradigms, owing to the spatial and temporal constraints of non-invasive neuroimaging. Nevertheless, related intracranial EEG studies have reported frequency-specific connectivity, including theta–alpha coupling between the hippocampus and entorhinal cortex during memory^22^, and theta-band amygdala–prefrontal interactions^23^.

Intracranial electroencephalography (iEEG) in neurosurgical patients offers a unique opportunity to bridge this gap, providing high temporal and spatial resolution to investigate the spectral signatures and directionality of amygdala-hippocampal interactions during emotional learning. Although such invasive data provide opportunities for fundamental neuroscience, their generalizability may be limited, as such recordings are only feasible in clinical populations. In this study, we leverage iEEG recordings from the human amygdala and hippocampus during a novel conditioning-extinction paradigm. We employ negative and neutral images from the International Affective Picture System (IAPS) as unconditioned stimuli (US), ensuring ecological validity and enhancing the generalizability of our findings^24^. This design allows us to isolate neural signatures specific to aversive learning while providing a non-aversive baseline to disentangle associative learning from general arousal effects. By examining both conditioning and extinction phases, we aim to delineate the oscillatory dynamics and directional interactions that support emotional memory formation and its suppression. We anticipated task-induced differences in the low frequency range (2–12 Hz) between conditioning (learning) and extinction phases, as well as between negative and neutral conditions within each phase, with rapid onset latencies for emotionally valenced stimuli, based on our previous amygdala neural recordings^25^. We also expected stronger theta connectivity based on recent findings of hippocampal theta sub bands^21^. On an exploratory basis, we also characterised interactions across various frequencies termed “cross frequency coupling (CFC)”, speculated to play a crucial role in the coordination of complex cortical computation^26^.

## Results

**Fig. 1:**
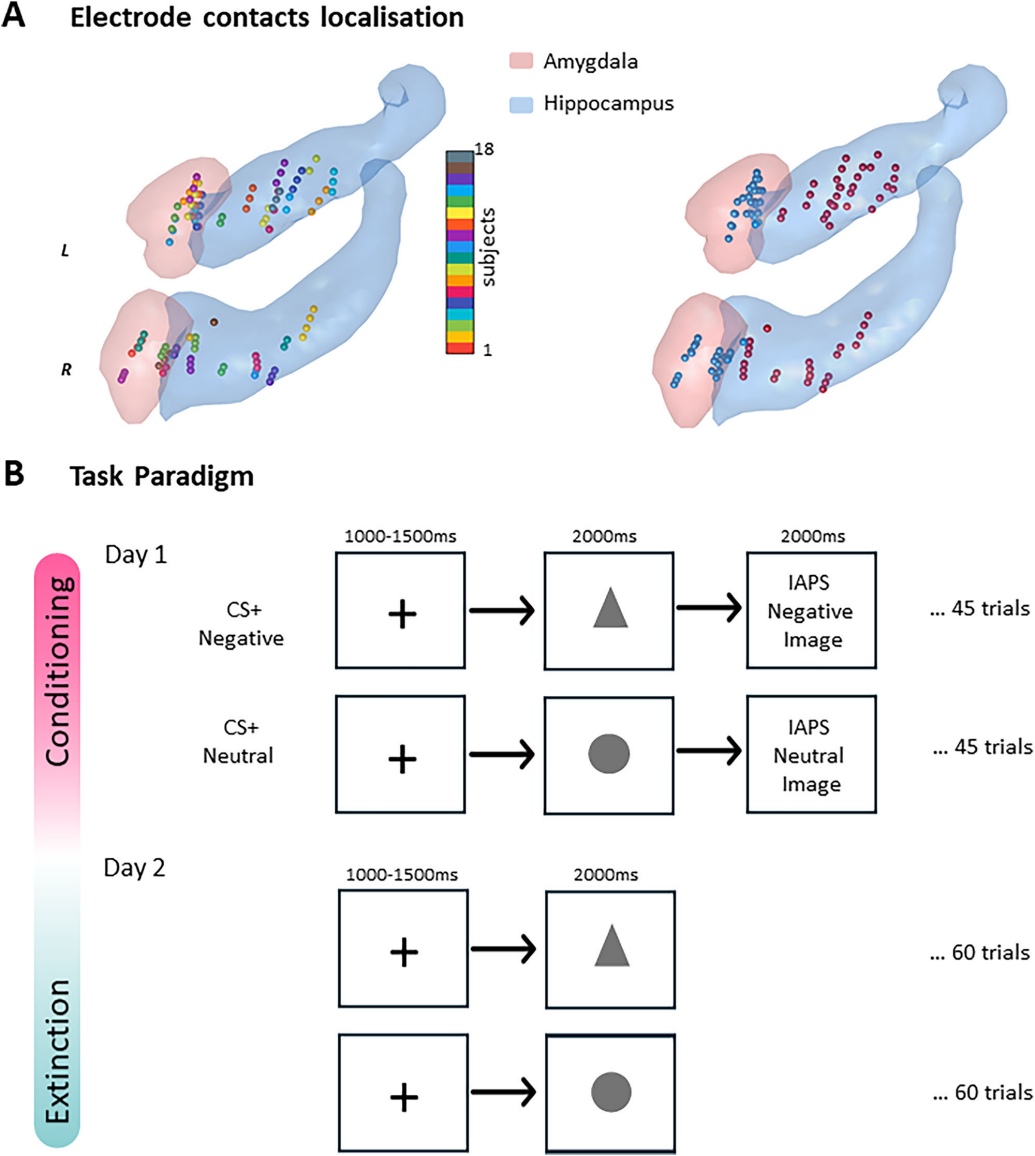
Localization of electrode contacts and task paradigm. **A** Amygdala shown in pink and hippocampus shown in blue in standard MNI brain space (L=left side brain, R=right side brain). Each dot is an electrode contact colour coded corresponding to each patient (left). Amygdala electrode contacts shown in blue and hippocampus contacts shown in red (right). **B** Conditioning and extinction task paradigm: The paradigm was conducted across two successive days in a clinical setting, with conditioning (pink) undertaken on Day 1 and extinction (teal) undertaken on Day 2. Conditioned stimuli (CS + ) consisted of a triangle or a circle presented for 2000 ms, followed by presentation of the unconditioned stimulus (US) for 2000 ms. The triangle was paired with a negative US, comprising negatively valenced images, whereas the circle was paired with a neutral US, comprising neutrally valenced images selected from the International Affective Picture System (IAPS). Extinction procedure was carried on Day 2 which followed similar process as conditioning except negative and neutral images were not shown. A fixation cross preceded each CS presentation and was jittered between 1000–1500 ms.

This study was undertaken in a cohort of 30 patients with refractory epilepsy who underwent stereo-electroencephalography neurosurgical procedure for clinical evaluation. Overall, there were 18 patients with a total of 53 contacts in the amygdala and 18 patients with 57 contacts in the hippocampus. Of these 14 subjects had concomitant electrodes in amygdala and the hippocampus. The electrode contacts localised in the amygdala and the hippocampus are shown in Fig. 1A. The conditioning-extinction paradigms is shown in Fig. 1B. The conditioning and extinction phase of the experiments were conducted on successive days. After preprocessing the data (see methods) we systematically evaluated the dynamics and condition difference of the amygdala and the hippocampal activity by employing event related spectral perturbation maps and using permutation testing to find frequency specific condition differences. Subsequently, we also computed high frequency broadband (HFB) activity (70-180 Hz) – a precise marker of neuronal firing^27^. Ultimately, comprehensive connectivity analytical approaches including computational modelling were utilised to probe the connectivity between amygdala and hippocampus.

### Amygdala Event related spectral perturbations (ERSPs)

**Fig. 2:**
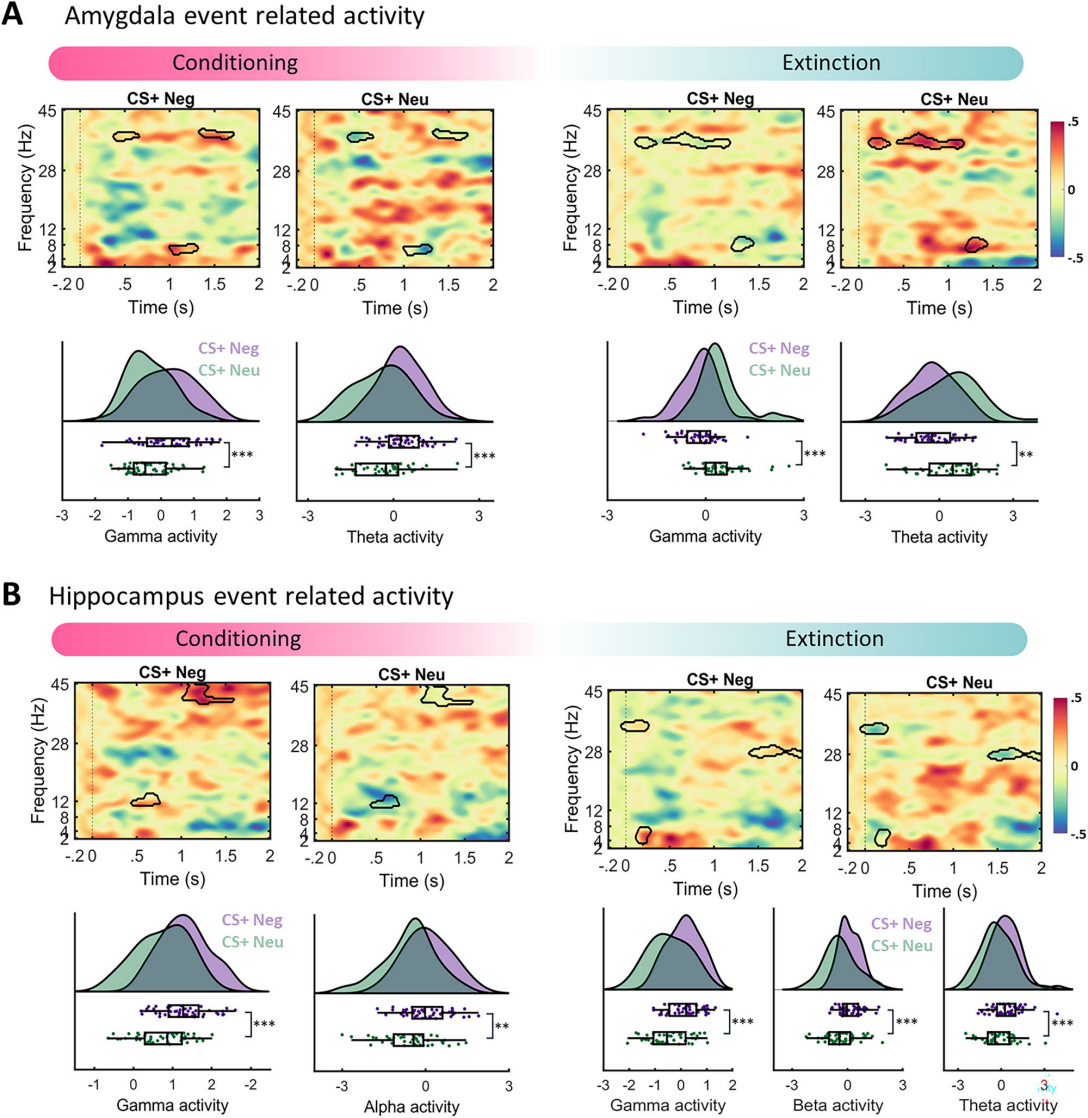
Task induced responses and condition differences. **A** *Amygdala:* Grand-averaged event-related spectral perturbation (ERSP) maps during conditioning (left) and extinction (right) for CS+ Negative (CS+ Neg) and CS+ Neutral (CS+ Neu) stimuli. Vertical dotted line indicates the onset of stimuli. Warmer colours indicate task-related increases in spectral power, whereas cooler colours indicate power decreases relative to baseline. Black contours denote time–frequency clusters showing significant differences between CS+ Neg and CS+ Neu conditions, identified using permutation testing. Raincloud plots below the ERSP maps summarize mean spectral power extracted from the significant clusters. Purple distributions represent CS+ Neg trials and green distributions represent CS+ Neu trials. Y-axis in these raincloud plots depict the distribution density and has no independent interpretive scale. Notably, theta- and gamma-band responses show a reversal in relative activity during extinction compared with conditioning. Analyses was undertaken on a total of 45 contacts in the amygdala from 18 patients. Two-sided t-tests were used. **B** *Hippocampus***:** Similar grand averaged ERSP maps as for amygdala during conditioning (left) and extinction (right). Raincloud plots below the ERSP maps summarize mean spectral power extracted from the significant clusters. Purple distributions represent CS+ Neg trials and green distributions represent CS+ Neu trials. Y-axis in these raincloud plots depict the distribution density and has no independent interpretive scale. Analyses was undertaken on a total of 45 contacts in the hippocampus from 20 patients. Two-sided t-tests were used.

In the time-frequency analysis of the aversive learning phase, the amygdala showed greater theta and gamma (~35 Hz) activity to negative compared to neutral CS+ (Fig. 2A*top*): gamma – CS+ negative: mean = 0.23, sd = 0.83), CS+ neutral: mean = −0.32, sd = 0.66), *T*_*44*_ = *4.10, P*_*FDR*_ = 0.0001); theta – CS+ negative: mean = 0.30, sd = 0.78, CS+ neutral: mean = −0.39, sd = 0.96), *t*_*44*_ = *3.54, P*_*FDR*_ = 0.0009). During the extinction phase, the same frequencies of theta and gamma showed reduced activity to negative compared to neutral CS+ (Fig. 2A*bottom*): gamma – extinction negative: −0.21 (0.57), extinction neutral: mean = 0.43, sd = 0.66, *t*_*52*_ = − *3.56, P*_*FDR*_ = 0.00003; theta – extinction negative: mean = −0.27, sd = 0.93), extinction neutral: mean = 0.48, sd = 1.21), *t*_*42*_ = − *3.17, P*_*FDR*_ = 0.0006).

### Hippocampal ERSPs

The hippocampus showed greater alpha and gamma activity to negative compared to neutral CS+ (Fig. 2B*top*): gamma – CS+ negative: mean = 0.30, sd = 0.61, CS+ neutral: mean = −0.25, sd = 0.65, *t*_*45*_ = *4.33, P*_*FDR*_ = 0.00008; alpha – CS+ negative: mean = 0.06, sd = 0.82; CS+ neutral: mean = −0.55, sd = 0.94, *t*_*45*_ = *3.16, P*_*FDR*_ = 0.002. During extinction increased activity to negative compared to neutral CS+ in the theta and gamma range was observed (Fig. 2B*bottom*): gamma – extinction negative: mean = 0.13, sd = 0.62, neutral: mean = −0.45, sd = 0.77, *t*_*45*_ = *3.87, P*_*FDR*_ = 0.0003; beta negative: mean = 0.16, sd = 0.60, extinction neutral: mean = −0.39, sd = 0.87, *t*_*45*_ = *4.06, P*_*FDR*_ = 0.0001; theta – negative: mean = 0.33, sd = 1.02), neutral: mean = −0.27, sd = 1.11, *t*_*42*_ = − *3.17, P*_*FDR*_ = 0.0006.

### Amygdala’s theta activity predicts emotional valence of negative and neutral USs

**Fig. 3:**
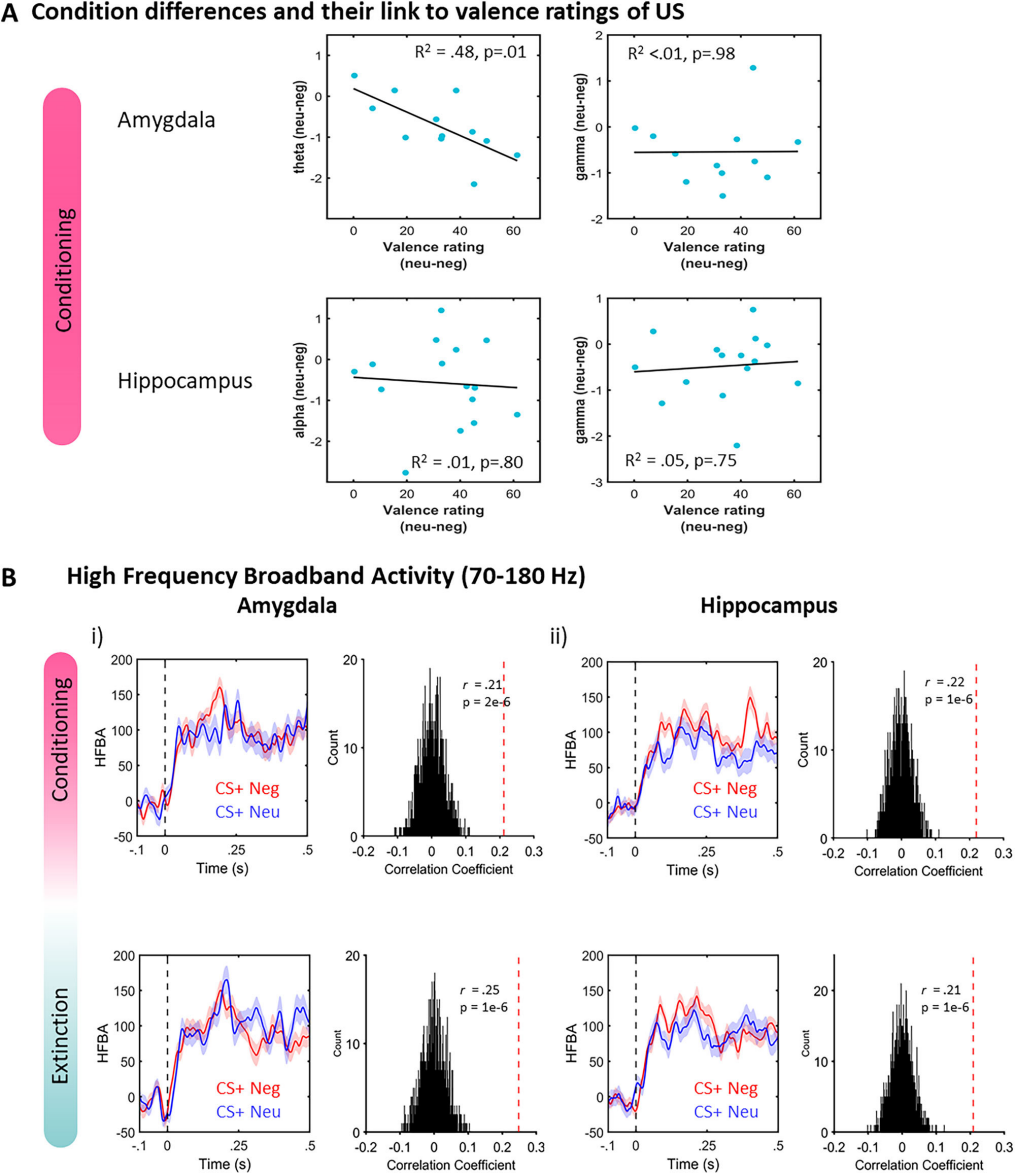
Event related condition differences during conditioning and their association with valence ratings of unconditioned stimuli and characterisation of high frequency activity profile. **A** *Frequency-specific activity and valence ratings*: Mean event-related activity for each frequency band cluster during the conditioning phase (Fig. 2) was computed separately for each participant. For both the amygdala and hippocampus, frequency specific condition differences were calculated as CS+ Negative (Neg) minus CS+ Neutral (Neu). This resulted in theta- and gamma-band difference measures for the amygdala, and alpha- and gamma-band difference measures for the hippocampus. These were associated with the mean valence ratings of the negative and neutral unconditioned stimuli with Pearson’s correlation corrected for multiple comparisons (see methods) (i) Amygdala: The mean theta-band difference between CS+ Negative and CS+ Neutral significantly linked to US valence ratings (left), such that larger differences in neutral versus negative valence ratings were associated with more negative neutral versus negative theta activity (greater CS+ Neg theta activity). In contrast, gamma-band activity did not show a significant association with valence ratings (right) (ii) Hippocampus: Neither alpha-band (left) nor gamma-band (right) activity differences showed a significant association with valence ratings. **B** *High frequency broadband activity* (*HFBA*) (i) Amygdala: HFBA during the conditioning phase for negative and neutral images (top) and for extinction phase (bottom) showing high similarity with adjacent plot showing statistically testing with permutation testing showing null distribution and correlation value in dashed vertical line. Shading indicates SEM. (ii) Hippocampus: HFBA during the conditioning phase for negative and neutral images (top) and for extinction phase (bottom) showing high similarity with adjacent plot showing statistically testing with permutation testing showing null distribution and correlation value in dashed vertical line. Shading indicates SEM.

We next assessed the relationship between condition differences and valence ratings of the USs on an exploratory basis. Subjects had rated the valence of the negative and neutral images (USs) in another experiment, the results of which have been published earlier^25^. To avoid multiple comparisons, we computed the difference of the mean cluster activity in each condition for each subject and linked it with the difference between neutral and negative valence ratings (see methods). Amygdalar theta activity predicted the relative valence ratings i.e. participants who rated negative images as more aversive (i.e., had larger neutral vs negative valence differences) showed greater theta power to CS+ negative stimuli compared to CS+ neutral ones (i.e., more negative y-values) (R^2^ = 0.48, **p = 0.01) (Fig. 3A). Amygdalar gamma (R^2^ = 0.0006, *p* = 0.98) and hippocampus alpha (R^2^ = 0.005, *p* = 0.80) and gamma (R^2^ = 0.007, *p* = 0.76) activity difference did not significantly relate to valence ratings. Significance was considered at 0.01 after multiple comparison correction.

### Similar HFB activity patterns in amygdala and hippocampus across negative and neutral conditions

We next examined whether high-frequency band (HFB) activity—a proxy for neuronal firing—exhibited similar patterns in the amygdala and hippocampus. Permutation tests revealed strong correlations between negative and neutral conditioning and in extinction (amygdala - conditioning: *r* = 0.21, *p* < 0.001; extinction: *r* = 0.25, *p* < 0.001) (Fig. 3B*left*) and extinction phases (hippocampus - conditioning: *r* = 0.22, *p* < 0.001; extinction: *r* = 0.21, *p* < 0.001) (Fig. 3B*right*). These results suggest that these regions may engage a common local processing mechanism in response to stimulus presentation and not strictly valence-specific responses.

### Amygdala-hippocampal connectivity

For connectivity analyses, we only included subjects with concomitant contacts in the amygdala and the hippocampus and used hierarchical connectivity approaches to investigate undirected functional connectivity, directed and effective connectivity. Specifically, we used weight phase lag index (wpli) for functional connectivity, transfer entropy for directed connectivity and dynamic causal modelling for effective connectivity. Here, we wanted to investigate the differences in learning and extinction, and consequently, we contrasted the conditioning vs extinction phases for negative and neutral conditions separately. Our systematic connectivity analyses revealed reconfiguration in distinct high and low theta bands.

### Weighted phase lag index (wPLI)

wPLI is a complementary phase measure which overcomes the limitations posed by volume conduction in coherence analysis^28^. This measure evaluates the consistency of non-zero phase lag relationships between two signals, providing a robust estimate of true interaction^29^.

**Fig. 4:**
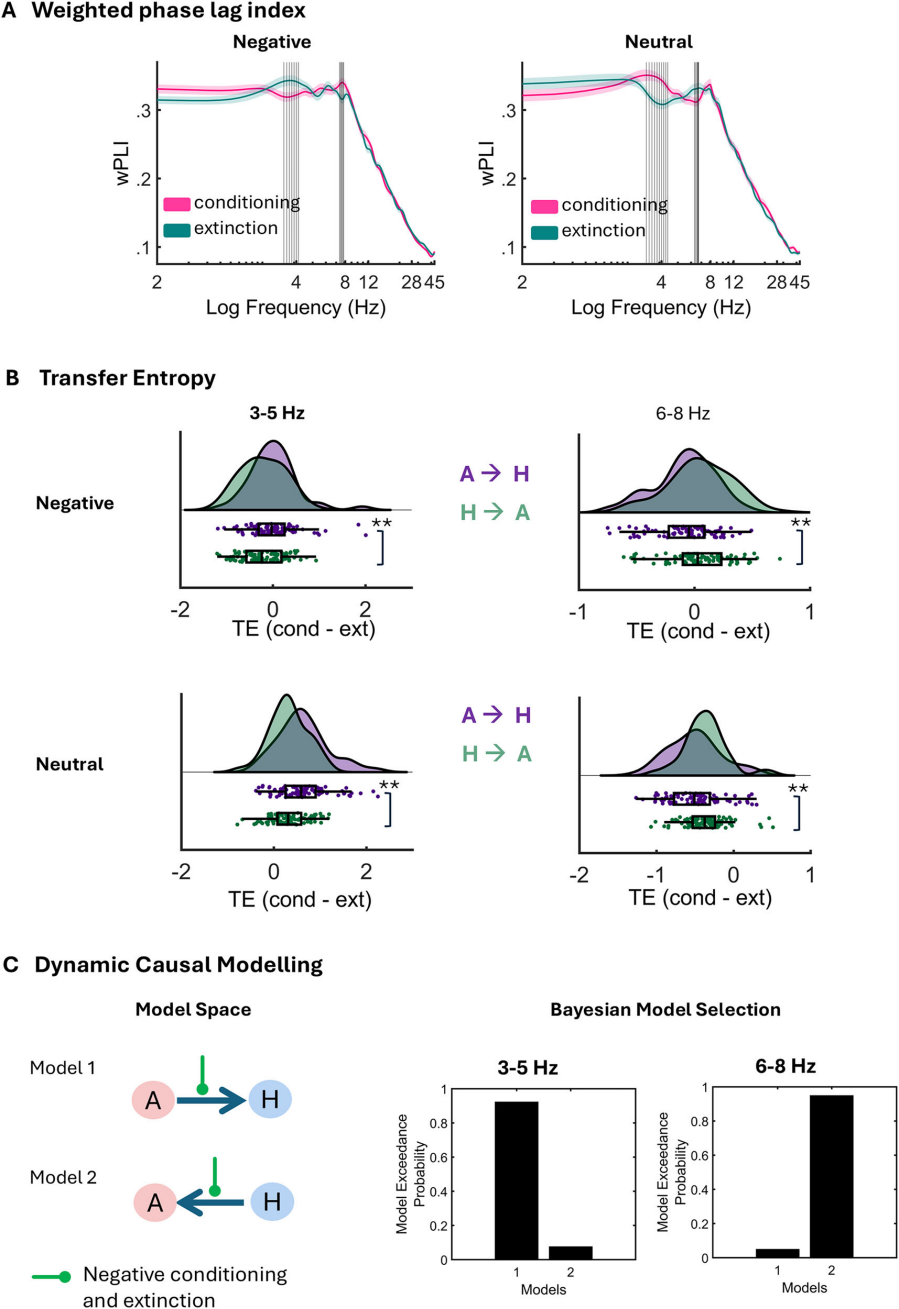
Amygdala-hippocampal connectivity. **A** *Weighted phase lag index* (*wPLI*)*:* connectivity differences in low theta (3–5 HZ) and high theta (6-8 Hz) were observed. Specifically, a double dissociation was found with lower low-theta and high theta connectivity in negative and neutral conditioning and extinction. Grey vertical lines show frequencies where significant differences were found with permutation testing (see methods). Shading indicates SEM. 90 pairs of amygdala-hippocampal contacts were used to derive these connectivity analyses. **B** *Transfer entropy* (*TE*)*:* Directional information flow between the amygdala and hippocampus was quantified using transfer entropy in the low theta (3–5 Hz; left) and high theta (6–8 Hz; right) frequency bands during conditioning (cond) and extinction (ext) for CS+ Negative (CS+ Neg) and CS+ Neutral (CS+ Neu) stimuli. TE was computed for the amygdala → hippocampus and hippocampus → amygdala directions. Condition differences were calculated as CS+ Neg in conditioning minus CS+ Neg in extinction and CS+ Neu in conditioning minus CS+ Neu in extinction, in for each direction and shown as raincloud plots. Y-axis in these raincloud plots depict the distribution density and has no independent interpretive scale. Statistical analyses revealed predominant amygdala-to-hippocampus information flow in the low-theta range, whereas hippocampus-to-amygdala information flow dominated in the high-theta range, for both negative and neutral conditions. Error bars indicate SD. ^*^p < 0.05, ^**^p < 0.01. Two-sided t-tests were used. 90 pairs of amygdala-hippocampal contacts were used to derive these connectivity analyses. **C** *Dynamic Causal Modelling:* For testing the effective connectivity in both 3-5 Hz and 6-8 Hz we employed a model space which comprised two models, with unidirectional connection from amygdala **A** to hippocampus **H** and from hippocampus to amygdala respectively and CS+ Negative conditioning and extinction task modulation effects influencing the connectivity. Bayesian model selection identified model 1 (amygdala to hippocampus) as possessing the highest exceedance probability (92.3%) for 3-5 Hz while for 6-8 Hz model 2 (hippocampus to amygdala) had the highest exceedance probability (94.9%). 90 pairs of amygdala-hippocampal contacts were used to derive these connectivity analyses.

wPLI results showed a double dissociation of connectivity: in the negative condition, we observed reduced low theta (3–5 Hz) and greater high theta-alpha (6–8 Hz) during conditioning with the opposite pattern observed during extinction (Fig. 4A*left*): low theta 3–5 Hz (CS+ negative: mean = 0.32, sd 0.06), extinction: 0.34, sd = 0.07, *T*_*89*_ = *-2.34, P*_*FDR*_ = 0.0001, p_FDR_ significance at 0.025; high theta 6–8 Hz (CS+ negative: mean = 0.32,sd = 0.06, extinction: mean = 0.34, sd = 0.07), *T*_*89*_ = *-2.34, P*_*FDR*_ = 0.0001, p_FDR_ significance at 0.025. For the neutral condition, wPLI results also showed a double dissociation of connectivity and opposite to that for negative condition: we observed greater low theta (3–5 Hz) and reduced high theta (6-8 Hz) during conditioning with the opposite pattern observed during extinction (Fig. 4A*right*): low theta 3–5 Hz - CS+ negative: mean = 0.32, sd 0.06, extinction: mean = 0.34, sd = 0.07 *T*_*89*_ = *-2.34, P*_*FDR*_ = 0.0001, p_FDR_ significance at 0.025; high theta 6-8 Hz - CS+ negative: mean = 0.34, sd = 0.06, extinction: mean = 0.32, sd 0.05, *T*_*89*_ = *2.66, P*_*FDR*_ = 0.009, p_FDR_ significance at 0.025.

### Transfer Entropy (TE)

TE is a powerful measure of directed connectivity as it captures the flow of information from one signal to another without assuming linearity or a specific model^30^. Unlike traditional correlation-based methods, it can detect asymmetrical and nonlinear interactions, making it especially well-suited for complex brain dynamics. Based on our wPLI results, we focused on low (3–5 Hz) and high theta (6–8 Hz) range to assess the directionality of information flow. We computed TE in both directions—amygdala-to-hippocampus and hippocampus-to-amygdala. To reduce multiple comparisons, we computed directional TE differences (conditioning minus extinction) for each frequency band and valence. A greater value indicates stronger connectivity during conditioning, while a lesser value indicates stronger connectivity during extinction. The results revealed a frequency-dependent directional pattern. For both the negative and neutral conditions, amygdala-to-hippocampus TE was significantly greater in the low theta range (3–5 Hz) i.e amygdala drove hippocampal activity during conditioning and hippocampus drove amygdala activity during extinction (negative stimuli conditioning: mean = 0.012, SD = 0.52; extinction: mean = –0.21, SD = 0.47; T₈₉ = 2.67, P_FDR_ = 0.009 (Fig. 4B*, top left*); neutral stimuli conditioning: mean = -0.04, sd = 0.26, extinction: mean = 0.049, sd 0.28, *T*_*89*_ = -*2.82, P*_*FDR*_ = 0.005 Fig. 4B*, bottom left*).

For both the negative and neutral conditions in the 6–sssssssssssssssssssssss8 Hz band, hippocampus-to-amygdala TE was significantly greater i.e hippocampus drove amygdala activity during conditioning and amygdala drove hippocampus activity during extinction (negative stimuli conditioning: mean = 0.63, SD = 0.40; extinction: mean = 0.32, SD = 0.54; T₈₉ = 3.51, P_FDR_ = 0.0006 (Fig. 4B*, top right*); neutral stimuli conditioning: mean = -0.50, sd = 0.34, extinction: mean = -0.36, sd 0.28, *T*_*89*_ = -*3.04, P*_*FDR*_ = 0.003 (Fig. 4B*bottom right*).

### Dynamic causal modelling (DCM)

DCM is a hypothesis-driven computational approach that estimates the direction of causal connectivity between brain regions, accounting for modulatory effects of experimental conditions^31^. It compares alternative models within a Bayesian framework to identify the most probable explanation for observed data^32^. We used DCM for phase coupling to assess how the phase of oscillatory activity in one region influences another over time^33,34^. Model spaces were informed by prior connectivity results of wPLI and TE, and we tested connectivity models in the 3–5 Hz and 6–8 Hz frequency bands. This task input modulates the strength of a directional connection in each model. For the 3–5 Hz band, the winning model robustly indicated amygdala-to-hippocampus connectivity with a probability of 92.3%, whereas in the 6–8 Hz band, the hippocampus-to-amygdala model was favoured with a probability of 94.9%. (Fig. 4C). In other words, the task (conditioning vs extinction) influences brain dynamics most clearly via amygdala’s influence on the hippocampus in 3-5 Hz and vice versa in 6–8 Hz. For neutral conditioning and extinction Bayesian model selection identified model 1 (amygdala to hippocampus) as possessing the highest exceedance probability (82.7%) for 3–5 Hz while for 6-8 Hz, model 2 (hippocampus to amygdala) had the highest exceedance probability (55%) (Supplementary Materials Fig. 1). These findings suggest that the directional motifs between amygdala and hippocampus are preserved across conditions but are robustly recruited only during negative emotional learning and extinction, which is consistent with emotional salience amplifying, rather than reconfiguring, amygdala–hippocampus theta dynamics.

### Cross Frequency Coupling

**Fig. 5:**
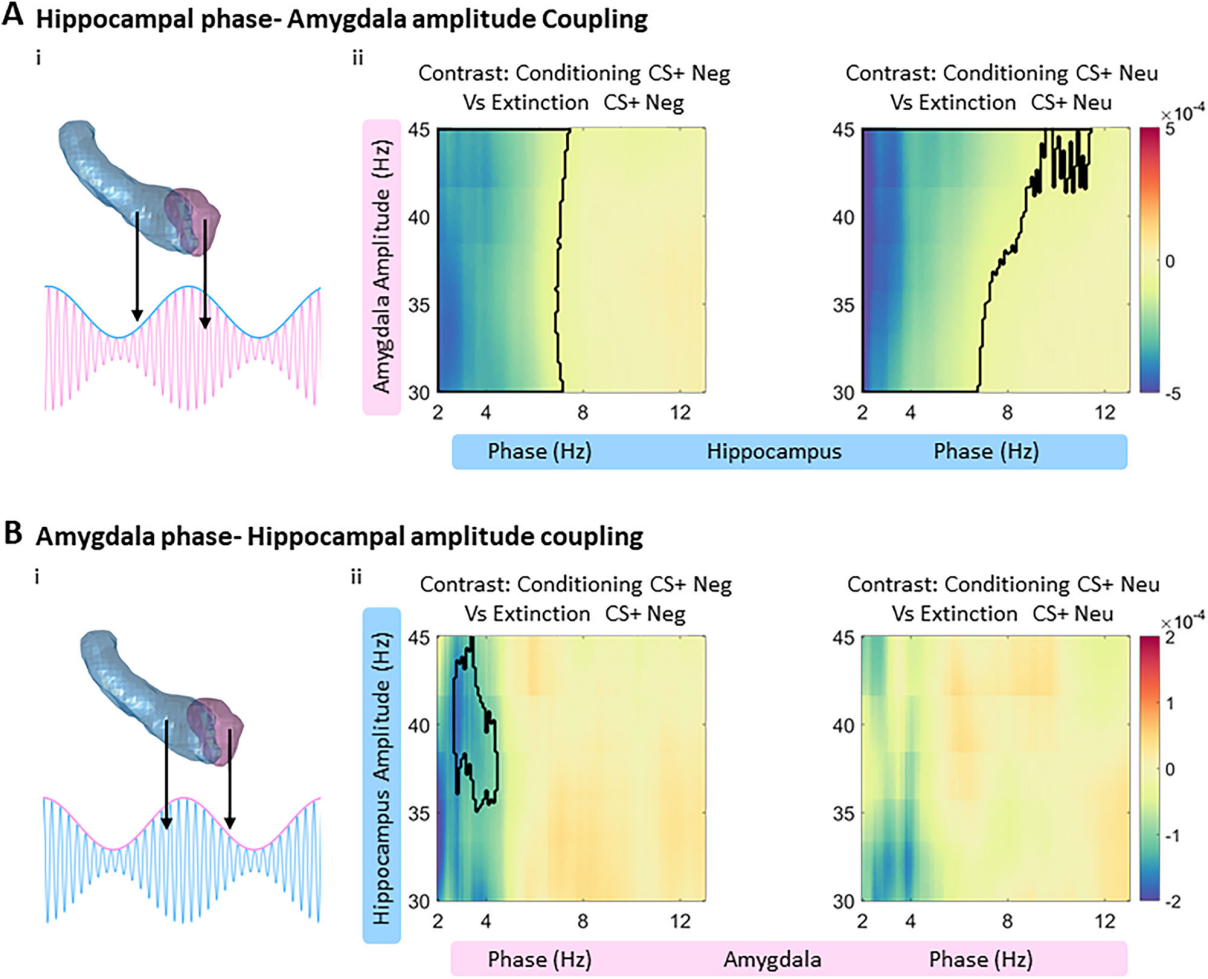
Phase–amplitude coupling (PAC) between the hippocampus and amygdala. **A** *Hippocampal phase–amygdala amplitude coupling* (i) illustration of hippocampal low frequency phase shown in blue and amygdala frequency amplitude shown in pink (ii) PAC comodulograms contrast between conditioning and extinction for CS+ Negative (left) and CS+ Neutral (right) stimuli. Black outlines denote clusters that reached statistically significant difference between the two conditions based on permutation testing. **B** *Amygdala phase– Hippocampal amplitude coupling* (i) illustration of amygdala low frequency (2−12 Hz) phase shown in pink and hippocampal frequency amplitude (30-45 Hz) shown in blue (ii) PAC comodulograms contrast between conditioning and extinction for CS+ Negative (left) and CS+ Neutral (right) stimuli. Black outlines denote clusters that reached statistically significant difference between the two conditions based on permutation testing (see methods).

These preceding results show the presence of local responses in these regions via coherent changes in gamma activity, together with functional and effective connectivity in low frequencies. We next asked whether there were condition differences in cross-frequency coupling (CFC) in conditioning and extinction between amygdala and hippocampus in low frequency phase activity and high frequency amplitude activity^26,35^. Specifically, how low frequency phase of the amygdala modulates high frequency activity of the hippocampus and vice versa. We undertook a similar approach as earlier to avoid multiple comparisons by subtracting the individual CFC maps of conditioning and extinction. To find the differences between CFC we used permutation testing. We observed significant differences between conditioning and extinction for both negative and neutral conditions where low frequency phase of hippocampus was modulating high frequency amplitude of amygdala activity (negative: conditioning mean = 0.0016, sd = 0.0006, extinction mean = 0.0019, sd = 0.0004, *t*_*89*_ =− *8.35, P*_*FDR*_ = 7e^-13^; neutral: conditioning mean = 0.0015, sd = 0.0006, extinction mean = 0.0017, sd = 1.11, *T*_*89*_ =− *8.35, P*_*FDR*_ = 2e^-12^) (Fig. 5A). For low frequency phase of amygdala modulating hippocampus high frequency amplitude, we only found significant differences for negative condition and the cluster of which was also much smaller (negative: conditioning mean = 0.0026, sd = 0.0003, extinction mean = 0.0028, sd = 0.0003, *T*_*89*_ =− *8.35, P*_*FDR*_ = 0.005). No significant differences were found for the neutral condition (Fig. 5B). These results support the predominant role of hippocampal phase-coordinating amygdalar gamma dynamics to gate associative memory updating across learning and extinction.

## Discussion

Understanding how the human brain flexibly learns, stores, suppresses and forms new emotional associations remains a significant challenge in neuroscience. Using intracranial recordings with high spatiotemporal resolution, our study provides direct evidence for frequency-specific oscillatory dynamics within the amygdala–hippocampal circuit during emotional learning and extinction. Complex high and low theta dynamics dominated amygdala-hippocampal connectivity with asymmetric dependencies from the amygdala to the hippocampus. Amygdala–hippocampal coupling supports emotional memory formation across valences, and our results should be interpreted as reflecting aversive learning within this broader functional framework. These findings advance our mechanistic understanding of how distinct frequency bands coordinate to support adaptive emotional behaviour from rich high-fidelity signals of which human neuronal data have been notably scarce.

### Frequency-selective reorganisation of amygdala–hippocampal activity

The observed increases in theta and gamma band activities in the amygdala during conditioning, particularly in response to negative conditioned stimuli (CS+ negative), underscore the amygdala’s role in processing emotional stimuli^1,15^. Specifically, theta oscillations in the amygdala are linked to the encoding of emotional salience^23^. Our previous studies have also attributed amygdalar gamma and beta activity to emotional valence^25^. Of note, here, theta response to CS+ (not the response to emotional USs), predicted subjective valence rating of USs, suggesting that theta oscillations encode both stimulus valence and emotional associations. Our findings of amygdala theta activity thus suggest a nuanced role in emotional processing beyond simple emotional recognition but rather of learned emotional association, expectation and appraisal. Gamma activity modulation has been observed in prior animal studies during fear conditioning^36^. Interestingly, the reversal of these spectral patterns during extinction i.e., reduced theta and gamma activity for negative relative to neutral trials in extinction, suggests a dynamic shift in amygdala engagement, likely reflecting its involvement in not only attenuating emotional responses to previously aversive stimuli but possibly also in the parallel unlearning of aversive associations^13^.

The hippocampus showed a complementary pattern, with an increase in alpha and gamma activity during conditioning, shifting to early theta and gamma during extinction. The transition from alpha to theta in these conditioning and extinction contexts might reflect a shift from a state of vigilance or alertness to one of memory reconsolidation or contextual updating, crucial for extinction learning^37^. Furthermore, hippocampal dynamics exhibited a temporal shift (time relevant to stimulus presentation i.e., 0-2000 milliseconds): during conditioning, late ~1 s alpha and gamma activity predominated, whereas extinction was characterised by early (300 ms) and increased theta and gamma activity. This early theta and gamma during extinction may also reflect learned prediction of the CS+ stimulus. Intricate links between gamma oscillations in the hippocampus arise from local circuit dynamics and are temporally organised by rhythmic theta inputs^38,39^. Overall, these are in line with findings suggesting that the hippocampus integrates contextual information with emotional content during memory formation and retrieval, contextual memory and flexible updating of learned associations^40–42^.

Extinction is widely considered to reflect new learning rather than erasure of the original fear memory^43^. Our results support this account by showing that both the amygdala and hippocampus remain engaged during conditioning and extinction, but with a reconfiguration of spectral power and directional connectivity. Conditioning and extinction were distinguished by frequency-specific shifts in theta-band communication and reversals in information flow between regions. This dynamic reorganization suggests that extinction arises from new learning implemented through altered amygdala–hippocampal coordination, rather than simple unlearning of prior associations.

### Valence-specific modulation of amygdala–hippocampal theta connectivity

Beyond local dynamics, our data reveal frequency-specific patterns of functional coupling between the amygdala and hippocampus that differ by both learning phase and stimulus valence. Prior animal work has shown that theta-band synchronization between these regions supports both fear conditioning and safety learning^44,45^. Extending these findings to humans, we identified a double dissociation in phase synchrony: low-theta (3–5 Hz) connectivity increased during extinction but decreased during conditioning for aversive stimuli, while high-theta/alpha (6–8 Hz) connectivity followed the opposite pattern. Notably, these dynamics were reversed in the neutral condition, underscoring the valence specificity of these effects.

These findings suggest that distinct theta sub-bands support divergent processes: low-theta may subserve fear consolidation or initial encoding of affective salience, while high-theta/alpha could mediate cognitive control and memory updating processes more active during extinction^46,47^. The specificity of these oscillatory modes for emotionally salient learning points to a frequency-based multiplexing mechanism within the amygdala–hippocampal circuit.

The observation of similar high-frequency broadband (70–180 Hz) activity in the amygdala and hippocampus across negative and neutral conditions suggests that local neuronal processing in these regions is not selectively driven by stimulus valence. Such broadband activity may reflect general associative processes that operate alongside lower-frequency dynamics and interregional connectivity for valence-dependent learning and its updating during extinction.

### Directional reorganisation of information flow in low and high theta synchrony

A notable finding is the directional reorganisation of connectivity, inferred through transfer entropy analysis and computational modelling. In negative and neutral conditioning and extinction, amygdala-led low-theta synchrony predominated, consistent with the amygdala’s established role in initiating affective salience signals. However, for high theta synchrony was driven by hippocampus to amygdala directionality, potentially reflecting the recruitment of contextual processing mechanisms during extinction^48^. Such directionality in phase coordination aligns with the theoretical framework that extinction is not the erasure of emotional memory but its contextual regulation^49^. By applying a DCM framework, which allows for the incorporation of experimental manipulations as modulators of effective connectivity, we systematically tested competing models of directional coupling. Dynamic causal modelling revealed that conditioning and extinction differentially modulated both the directionality and frequency of amygdala–hippocampal interactions. During conditioning, connectivity was best explained by a 3–5 Hz amygdala-to-hippocampus model, consistent of the amygdala influence during aversive learning. In contrast, during extinction, the winning model shifted to a 6–8 Hz hippocampus-to-amygdala direction, suggesting a reversal of information flow i.e., hippocampus to amygdala, suggesting contextual modulation during safety learning^44,50^. Thus, although both frequency phases engaged amygdala–hippocampal coupling, they were characterised by distinct frequency bands and opposing directions of effective connectivity, rather than reflecting the same underlying mechanism. Oscillatory dynamics from hippocampal neural recordings are consistent with our findings of distinct theta sub-bands^21^.

Our findings align closely with human fMRI work demonstrating that fear conditioning and extinction rely on coordinated amygdala–hippocampal–prefrontal networks. fMRI studies have shown dynamic changes in functional coupling between the amygdala and hippocampus across conditioning and extinction, alongside increased engagement of medial prefrontal regions during extinction and extinction recall^5,14^. More broadly, human neuroimaging consistently implicates reconfiguration of this network during suppression and updating of aversive memories. The present iEEG results extend these findings by revealing the spectral and directional mechanisms—specifically theta sub-band–dependent bidirectional interactions—that underlie these large-scale fMRI connectivity patterns at a finer temporal scale.

### Incorporating neutral stimuli as non-aversive baseline condition

Our inclusion of a neutral conditioned stimulus control condition was instrumental in isolating the effects specific to aversive learning. This methodological choice allowed us to distinguish general learning effects from those uniquely tied to aversive emotion, thereby strengthening our conclusions about the role of theta-gamma dynamics in emotional learning^7^. Instead, they emerge preferentially in the context of emotionally salient learning, pointing to a valence-dependent recruitment of neural synchrony. In this way, our findings support the view that emotional learning is governed by dedicated neural mechanisms, rather than being a more intense version of neutral learning.

### Limitations and conclusion

This study has several limitations. First, our data come from patients with epilepsy which may limit generalisability. However, we adopted strict screening to only include electrode contacts not diagnosed as epileptogenic zones for our analyses. This limitation must be weighed against the unique strengths of iEEG, which provides direct recordings of neural population activity that are not accessible with non-invasive techniques such as functional MRI (fMRI) which measures slow hemodynamic response (~5 seconds) while EEG/MEG are constrained by poor spatial localization, particularly in deeper brain structures such as amygdala and hippocampus. Furthermore, we employed visual inspection of the data to remove noisy trials. Amygdala and hippocampus are composed of multiple nuclei and thus precise functionality of each subnuclei cannot be differentiated with these recordings. Subnuclear dissociation would be an important direction for the field and highlight future work using higher-density electrodes (e.g., Neuropixels-like designs with <1 mm spacing), larger datasets with balanced subnuclear coverage, and convergent imaging or histological validation to directly test differential basolateral amygdala (BLA) versus central amygdala (CeA) contributions to oscillatory dynamics during fear learning and expression. Furthermore, it is conceivable that recordings are influenced by nearby contacts in various brain structures. However, we used bipolar referencing in our analysis, which mitigates against these volume conduction issues^51^. Prefrontal contributions (including ACC and OFC) and their interactions with the amygdala and the hippocampus during learning and extinction are also critical, which should be systematically examined in future. Finally, we acknowledge that IAPS images, while providing ecological validity and standardised presentation, may elicit milder aversive responses compared to primary unconditioned stimuli such as electric shocks, and thus may not fully engage all fear-related circuits, particularly those mediating intense autonomic or defensive responses. However, we employed IAPS images as US, which are widely investigated in neuroimaging and easier to develop paradigms with, thus increasing the wide use of such conditioning-extinction paradigm. Furthermore, incorporating both positive and negative valenced stimuli will be essential for disentangling shared versus valence-specific dynamics within amygdala–hippocampal circuits. In this study, no explicit behavioural indices of learning were collected during the conditioning/extinction phases, which limits investigations of learning outcomes. We did not incorporate gender and age into our analyses, which have been shown to influence emotional memory processing. Intracranial EEG studies are necessarily constrained by small and clinically heterogeneous samples, and subgroup, laterality or covariate analyses for sex or age would be statistically underpowered. Larger sample sizes with harmonised datasets can investigate this in future, as well as to establish robust amygdala-hippocampal connectivity profile.

In sum, our study provides compelling evidence that emotional learning and extinction are mediated by a dynamic reconfiguration of amygdala–hippocampal circuitry across distinct frequency bands. By identifying a double dissociation in low- and high-theta connectivity and revealing directional shifts in information flow across learning phases, we offer a mechanistic account of how the brain flexibly encodes and subsequently updates emotional associations. These findings enrich current models of affective flexibility and suggest new spectral targets for neuromodulation therapies in disorders characterised by impaired extinction learning, such as PTSD^52,53^. The observed frequency-specific dissociation highlights oscillatory markers as potential biomarkers of treatment engagement or response, enabling patient stratification by symptom severity. Importantly, this study identifies two anatomical targets for neuromodulation. Theta burst stimulation (TBS; 5 Hz), currently FDA-approved for psychiatric conditions such as depression, could be adapted to target the amygdala or hippocampus in PTSD and anxiety disorders. Specifically, lower-theta inhibitory stimulation of the amygdala or higher-theta inhibitory stimulation of the hippocampus may modulate amygdala-hippocampal connectivity, offering a potential therapeutic approach for these disorders. Future work should investigate how these oscillatory dynamics interface with prefrontal regions and whether they can be harnessed to guide personalised interventions for affective dysregulation.

## Methods

### Participants

The study involved a cohort of 30 patients diagnosed with medically refractory epilepsy who underwent stereotactic electrode implantation for clinical evaluation at Ruijin Hospital in Shanghai. Participants were selected based on the following criteria: (1) age between 18 and 60 years, (2) right-handedness, (3) a Montreal Cognitive Assessment (MoCA) score of 24 or higher, and (4) electrode implantation in at least one of the following regions: the amygdala or hippocampus. The decision on electrode placement was determined solely by clinical considerations. Patient demographics are presented in Table 1. All participants provided written informed consent and retained the right to withdraw from the study at any stage. The study received approval from the Human Research Ethics Committees of Ruijin Hospital, Shanghai, China, and was conducted in accordance with the principles outlined in the Declaration of Helsinki.

**Table 1:** Patient demographics

PID	Sex	Age (yrs)	Handedness
P1	F	53	R
P2	F	20	R
P3	F	23	R
P4	M	19	R
P5	M	18	R
P6	F	24	R
P7	M	19	R
P8	M	24	R
P9	F	23	R
P10	M	38	R
P11	M	21	R
P12	F	36	R
P13	M	39	R
P14	M	20	R
P15	F	30	R
P16	M	43	R
P17	F	46	R
P18	F	25	R

### Data acquisition

#### Electrode localisation (Fig. 1A)

Intracranial electrodes were localised on a 3-D MNI brain template using a fusion of pre-operative magnetic resonance (MR) and post-operative computed tomography (CT) scans using Brainstorm^54^. We started by co-registering the CT image to MR T1 image and normalising them to the MNI template. The co-registered and normalised CT and MR images were then visually inspected for the anatomical location of the electrode channels. The electrode channels were also assessed on the AAL atlas [38] to confirm their location in the anatomical regions of interest. The electrode channels are shown in MNI space (Fig. 1). The implantation maps of the electrode contacts localised by the neurosurgeon were also used to corroborate the presence of contacts in the anatomical regions of interest. Only electrode contacts from the non-epileptogenic zone as diagnosed by the clinicians, were used for analyses. Overall, there were 18 patients with a total of 53 contacts in the amygdala and 18 patients with 57 contacts in the hippocampus. Of these 14 subjects had concomitant electrodes in the amygdala and the hippocampus. The mean age of the participants was 28.94 yrs (9 females). In our dataset, the inter-contact spacing in the electrodes is 1.5 mm, while the spatial spread of local field potentials is estimated to be ~2–3 mm, meaning that individual contacts likely sample activity from multiple neighbouring nuclei^55–57^. Therefore, we did not undertake subnuclear localisation of amygdala and hippocampus which may risk producing spurious or misleading results.

#### Stereo-EEG recordings and preprocessing

Intracranial electrodes with multiple contacts (Huake-Heushang SDE 08, containing 8–18 contacts, electrode diameter: 1 mm, intercontact spacing of 1.5 mm) were implanted using a stereotactic approach. The placement of electrodes was customized for each patient, guided by the likely seizure onset zone determined by clinicians (SZ, WL, and CC) based on non-invasive preoperative assessments. Intracranial EEG (iEEG) signals were recorded at a sampling rate of 1 kHz using a Brain Products amplifier, which had a 32-channel capacity for the first three patients and a 64-channel capacity for the remaining participants. Data from experimental tasks were analyzed offline utilizing EEGLAB^58^ and custom MATLAB (MathWorks, MA, USA) scripts. Electrode contacts which had noisy or flat recordings were excluded from the analyses. The recordings were downsampled to 500 Hz and band-pass filtered between 0.1 and 195 Hz using a zero-phase lag finite impulse response (FIR) filter. To remove power line interference, 50 Hz noise and its harmonics were notch filtered. Additionally, a bipolar re-referencing scheme was applied to the data. Overall, there were 18 patients with a total of 45 contacts in the amygdala, 20 patients with 45 contacts in the hippocampus.

#### Paradigm

Illustration of the task paradigm is shown in Fig. 1B. During the task, patients were instructed to view the stimuli displayed on the screen. Two different shapes (triangle and circle) constituted the CS. CS+ was reinforced with negative or neutral valence images from the International Affective Picture System (IAPS)^24^ which acted as the US (US; contingency of 100%). 40 images each of the negative and neutral categories, were selected. CS+ were presented 45 times each and were presented on a desktop screen 60cms away from the participant in random order. Intertrial interval consisting of a black fixation cross shown on a white background was jittered between 1000-1500 ms. Each CS+ and US lasted for 2 s. During the intertrial interval, a black fixation cross was shown on a white background. The US delivery occurred upon the termination of CS+ (i.e., delay conditioning). We did not undertake responses for the establishment of a conditioned response as this is arguably independent of conscious contingency awareness^59,60^. Further, we used 100% probability for reinforcing with USs.

Extinction testing was conducted on the following day, approximately 24–30 hours after conditioning, as clinical constraints precluded testing at an exact 24-hour interval. During the extinction phase, only CS+ stimuli were presented for 2 seconds, and these were not followed by either aversive or neutral unconditioned stimuli. The precise time-based control of sending trigger pulses for the paradigms was programmed with Psychtoolbox^61^ run on MATLAB version 2018b (The MathWorks, Inc., Natick, MA), interfaced via a parallel port. Due to logistical constraints and to avoid long experimental sessions, inherent to conducting intracranial EEG studies in clinical settings, we did not acquire any explicit behavioural measures of conditioning and extinction.

### Event Related Spectral Perturbation (ERSP)

Task induced changes were quantified and statistically tested with cluster-based permutations tests as employed in our previous work^25,62^. Specifically, event-related spectral perturbation (ERSP) quantifies changes in the power of ongoing neural oscillations across specific frequency bands and time points, relative to a baseline^58^. For our analyses, we used a 500 ms pre-stimulus period as baseline and examined a 2000 ms window following stimulus onset. ERSPs were computed using the fast Fourier transform (FFT) across 2–45 Hz in EEGLAB. These ERSP maps were first generated for individual electrode contacts (frequency × time) and then aggregated across contacts within each region of interest to produce three-dimensional datasets (frequency × time × contacts).

### High Frequency Broadband Activity (HFBA)

High-frequency activity (HFA) was estimated using high-frequency broadband amplitude (HFBA) following established approaches^63^. Preprocessed signals were then band-pass filtered between 70 and 180 Hz using consecutive 10-Hz frequency bands (70–80, 80–90, …, 170–180) with a zero-phase, two-pass finite impulse response (FIR) filter implemented in EEGLAB. The analytic amplitude of each band-limited signal was extracted using the Hilbert transform. These specific amplitudes were expressed as percentage change relative to this baseline (200-ms pre-stimulus) and averaged across 10 Hz frequency bands.

### Connectivity analyses

For connectivity analyses, only those subjects who had concomitant electrodes in the amygdala and the hippocampus were used in our analyses. We used all possible combinations of the contact pairs in amygdala-hippocampus dyad for each subject to compute connectivity analyses. No contralateral amygdala–hippocampus interactions were analysed, in recognition of known anatomical constraints. In two participants who had bilateral depth electrode coverage of both the amygdala and hippocampus, analyses were restricted to the hemisphere with a greater number of contacts. Concomitant contacts in both the amygdala and the hippocampus were available for 14 patients. However, with the removal of noisy recordings, we were left with 90 possible dyadic pairs.

#### Weighted phase lag index (wPLI)

The Weighted phase lag index (wPLI) was computed to quantify phase-based functional connectivity while minimising the influence of volume conduction and noise^28^. This measure evaluates the consistency of non-zero phase lag relationships between two signals, providing a robust estimate of true interaction^29^. The cross-spectrum of signal pairs was computed using multitaper spectral estimation, yielding the imaginary component of the cross-spectral density and wPLI was then calculated as:

$${wPLI}=\frac{|{\mathbb{E}}[{Im}({S}_{{xy}})]|}{{\mathbb{E}}[{|Im}({S}_{{xy}})|]}$$

where Im(*Sxy*) is the imaginary part of the cross-spectral density between signals *x* and *y*, and E denotes the expectation across epochs. To further reduce bias from limited sample sizes, the debiased wPLI (dwPLI) was computed, correcting for statistical bias by incorporating the variance of the imaginary components.

#### Transfer Entropy Estimation

Transfer entropy (TE) was used to estimate the directed information flow between two EEG channels during task-related epochs. EEG data were preprocessed using EEGLAB, including filtering in the theta band (3–5 Hz) and high theta band (6–8 Hz). For each subject, trials were extracted from two selected channels and transformed into trial-wise time series. TE was computed bidirectionally (channel 1 → channel 2 and channel 2 → channel 1) using a non-parametric k-nearest neighbour (k-NN) estimator implemented in the ITE toolbox (version 0.63). This method, based on mutual information theory, reliably estimates TE in continuous, non-Gaussian neural data without assuming linear interactions Vicente, R., et al. (2011) Schreiber, T. (2000). Parameters were set to a delay embedding of τ = 35 samples and k = 8 neighbours, values previously validated for neurophysiological data [2]. For each trial, TE was computed independently and averaged across trials to yield subject-level estimates of directional information flow. This approach allows robust, model-free quantification of dynamic, nonlinear interactions between brain regions and has been widely validated in both EEG and intracranial recordings^64^.

#### Dynamic Causal Modelling (DCM) for Phase Coupling

To infer effective connectivity between brain regions based on oscillatory phase relationships, we employed Dynamic Causal Modelling for Phase Coupling (DCM-PC), a validated generative modelling framework implemented in SPM12^65^. DCM-PC is designed to estimate the coupling of neural oscillators based on the phase time series extracted from band-limited electrophysiological data (typically EEG or MEG)^66^. This approach models each region as a limit-cycle oscillator and characterizes their interactions in terms of changes in phase relationships over time, enabling inference on the directionality and strength of coupling between sources. DCM-PC has been shown to offer a principled way of modelling frequency-specific interactions in oscillatory brain networks and is particularly useful for capturing non-linear and dynamic coupling mechanisms^31,33^.

We applied Dynamic Causal Modelling for Phase Coupling (DCM-PC)^33^ to examine directed phase-based interactions between the amygdala and hippocampus by modelling these regions as coupled oscillators where one region’s phase evolution depends on its phase difference relative to the other. Based on our prior wPLI and TE results, we specified two competing unidirectional models (amygdala→hippocampus and hippocampus→amygdala) with connectivity modulated by experimental conditions coded as +1 for conditioning and -1 for extinction. We only inverted DCMs for negative conditions as this modelling approach is computationally intensive, and we had a strong hypothesis based on our previous results. Using Bayesian model inversion, we estimated coupling parameters and employed random-effects Bayesian Model Selection^67^ to compare model evidence and identify the most likely directional interaction pattern. The winning model was determined based on the highest exceedance probability (≥0.90) in random-effects Bayesian model selection, indicating strong evidence for its superior explanatory power relative to competing models in characterizing the observed phase coupling dynamics^67^.

### Cross Frequency Coupling

We computed phase-amplitude coupling (PAC) using a validated modulation index approach^68^. For each participant and each condition trials, we extracted phase time series (2–14 Hz, 4 Hz bandwidth) and amplitude envelopes (30–45 Hz, 15 Hz bandwidth) through bandpass filtering and Hilbert transformation. The phase-amplitude relationship was quantified by binning gamma amplitudes according to low-frequency phase (15 bins) and calculating the Kullback-Leibler divergence from a uniform distribution. This produced trial-specific comodulograms that were averaged across trials for each electrode pair and each condition during conditioning and extinction. The resulting PAC metrics were stored for subsequent group-level comparisons between experimental conditions using permutation based statistical testing described below. For visualisation we show the contrast between the CS+ Neg for conditioning and extinction as well as and CS+ Neu for conditioning and extinction. This allowed specific PAC profiles that were different between learning and extinction.

### Statistical testing

To compare time frequency maps (ERSPs) between positive (relative to neutral) and negative conditions (relative to neutral), a nonparametric cluster-based permutation approach^69^ and has been previously employed by us^25,62^. This approach specifically corrects for multiple comparison issues in multidimensional data such as time-frequency decompositions or time domain data. It enables identification of clusters (time windows and/or frequency bins) with significant differences in the power changes induced by the presentation of pictures of different emotional valence. For permutations, the original paired samples for each region (condition ERSPs/connectivity metrics for each contact) were randomly permuted 1000 times such that each pair was maintained but its assignment to the condition (positive or negative) changed to create a null-hypothesis distribution. For ERSP maps, or wPLI or PAC maps, we applied a strict significance level of *p* < 0.01 for cluster differences. We restricted the size of time frequency clusters to>500 to find robust condition differences and to avoid small, potentially meaningless clusters. Average power in the significant clusters was further compared using post-hoc paired t-tests with multiple comparison false discovery rate (FDR) correction^70,71^. The condition differences were plotted raw time frequency plots (i.e., without subtraction of neutral condition) to appreciate early task induced activity and condition differences.

For the association of the mean of significant clusters in traditional frequency ranges from each patient with corresponding valence ratings, linear regression was employed with p significance at 0.01 after multiple comparisons corrections. Mean event-related activity for each frequency band cluster during the conditioning phase (Fig. 2) was computed separately for each participant. For those with multiple contacts in the region of interest, we averaged the significant cluster for those contacts. For both the amygdala and hippocampus, frequency-specific condition differences were calculated as CS+ Neutral (Neu) minus CS+ Negative (Neg). This resulted in theta- and gamma-band difference measures for the amygdala, and alpha- and gamma-band difference measures for the hippocampus. These were associated with the mean valence ratings of the negative and neutral unconditioned stimuli with Pearson’s correlation corrected for multiple comparisons.

The similarity of HFB activity for negative and neutral conditions was statistically tested by first obtaining the mean correlation between the HFB time series of all contact pairs for each condition. It was tested against a null distribution, which was obtained by circularly shifting the time series 1000 times and the mean correlation was computed for each iteration^72,73^. This method preserves the autocorrelation properties of the signal and provides a robust method for testing the similarity or dissimilarity of signals.

### Reporting summary

Further information on research design is available in the Nature Portfolio Reporting Summary linked to this article.

## Supplementary information

Supplementary Information

Reporting Summary

Transparent Peer Review file

## Source data

Source Data 1

Source Data 2

Source Data 3

Source Data 4

## Data Availability

The data for this project were acquired from patients undergoing clinical care and consenting to additional research protocols. Researchers wishing to access these data will require local ethics approval and a data sharing agreement with Ruijin Hospital, Shanghai, China. Open-source toolboxes have been used for analyses of this study. Open-source toolboxes have been used for analyses of this study. Specific codes are made available on github https://github.com/srbsonkusare/amygdala_hippocampal_dynamics_in_conditioning-extinction. Source data are provided with this paper.

## Code availability

The Code for this project were acquired from patients undergoing clinical care and consenting to additional research protocols. Researchers wishing to access these data will require local ethics approval and a data sharing agreement with Ruijin Hospital, Shanghai, China. Open-source toolboxes have been used for analyses of this study. Open-source toolboxes have been used for analyses of this study. Specific codes are made available on github https://github.com/srbsonkusare/amygdala_hippocampal_dynamics_in_conditioning-extinction.
