# Supplementary material for: Adaptive shifts in amygdala–hippocampal theta coupling govern aversive learning and extinction
Source: Nat Commun. 2026 Jun 10;17:7835. doi: 10.1038/s41467-026-73967-4 (PMC13439568; doi:10.1038/s41467-026-73967-4)
Supplement: Supplementary file 1 [file 41467_2026_73967_MOESM1_ESM.pdf]

# Adaptive shifts in amygdala–hippocampal theta coupling govern aversive learning and extinction

Saurabh Sonkusare, Qiong Ding, Christopher Weirich, Yashu Feng, Wei Liu, Ruoqi Yang, Alekhya Mandali, Samantha Sallie, Violeta Casero, Chunyan Cao, Dianyou Li, Bomin Sun, Shikun Zhan, and Valerie Voon

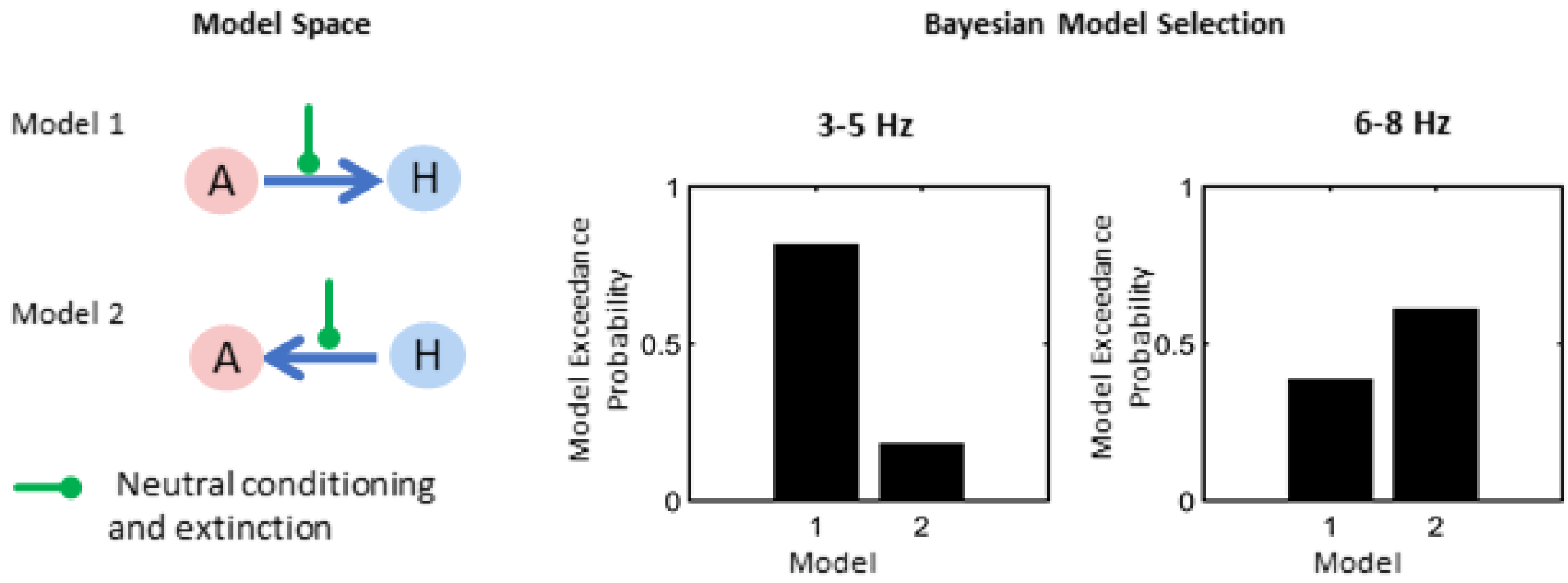


**Supplementary Figure 1: Dynamic Causal Modelling for neutral condition***:* For testing the effective connectivity between the amygdala and the hippocampus for neutral conditions in 3-5Hz and 6-8Hz, we employed a model space which comprised two models identical to that used for negative condition (Figure 5C). Specifically, models comprised of unidirectional connection from amygdala (A) to hippocampus (H) and from hippocampus to amygdala respectively with CS+ Neutral conditioning and extinction task modulation effects influencing the connectivity. Bayesian model selection identified model 1 (amygdala to hippocampus) as possessing the highest exceedance probability (82.7%) for 3-5 Hz while for 6-8 Hz model 2 (hippocampus to amygdala) had the highest exceedance probability (55%).
