# Supplementary material for: Adaptive shifts in amygdala–hippocampal theta coupling govern aversive learning and extinction
Source: Nat Commun. 2026 Jun 10;17:7835. doi: 10.1038/s41467-026-73967-4 (PMC13439568; doi:10.1038/s41467-026-73967-4)
Supplement: Supplementary file 3 [file 41467_2026_73967_MOESM3_ESM.pdf]

nature portfolio

Peer Review File

Adaptive shifts in amygdala–hippocampal theta coupling govern aversive learning and extinction

Corresponding Author: Dr Saurabh Sonkusare

This file contains all reviewer reports in order by version, followed by all author rebuttals in order by version.

Version 0:

Reviewer comments:

Reviewer #1

(Remarks to the Author)
Sonkusare and colleagues report on the dynamics of amygdala-hippocampal theta coupling during aversion learning and extinction via recording in human epileptic patients during an aversive and neutral conditioning paradigm and subsequent extinction.

The paper reads well and uses multiple analytic methods to examine LFP interactions across structures. The authors also do a nice job of referencing a considerable number of analytic/methods papers for examining LFP/EEG interactions across structures. The results generally support dynamic fluctuations in amygdala-hippocampal interactions as one might expect during such a learning task.

While I understand the focus on aversive conditioning, it is perhaps unfortunate that the authors did not include a positive CS+. The paper reads as if amygdala-hippocampal interactions largely support aversive conditioning, while invariably amygdala-hippocampal interactions support both +/- conditioning processes and the formation of post positive/negative emotional memories. Some comment on this somewhere in the manuscript may be appropriate.

Nonetheless, I find the findings noteworthy although some greater integration with similar/related findings from amygdala/hippocampal/entorhinal/MTL structures in the growing # of human epileptic recording papers may be helpful. The discussion went over the finding of the current paper well, but perhaps did not integrate well with other/similar/related findings in the field.

I have some minor to moderate suggestions for improvement as explicated below in order of occurrence. Generally, several/most of the figure legends could be significantly enhanced with additional explications as outlined below. Some of the figure legends are quite sparse on content.

Abstract

Minor: Lines 27-29 “Adaptive behaviour relies on the flexible encoding and suppression of aversive associations, yet the spectral and directional dynamics underlying amygdala–hippocampal interactions in humans remain poorly understood.

Perhaps insert “often” and separate into two sentences .. ”Adaptive behaviour often relies on the flexible encoding and suppression of aversive associations supported by amygdala-hippocampal interactions. The latter remains poorly understood and is the focus of the current report.”
There is “adaptive behavior” that is quite independent of amygdala-hippocampal interactions.
Introduction

Minor: Line 61 “These processes are not only fundamental learning”

Add “support… “These process support not only fundamental learning”, as there is perhaps more to “fundamental learning” then “These processes”.

Moderate: Line 76+ “Nevertheless, the conditioning-extinction paradigm has established a distributed

network……..prefrontal cortex. Within this circuit, the amygdala and hippocampus….
The paradigm didn't "establish" the network, one might write the paradigm "revealed" or "provided insight into" a distributed network.

Next sentence uses "circuit" instead of "network". There is not hard/fast definition of circuits vs networks, but I think of circuits as well-grounded, anatomical circuits, eg, EC-HPC microcircuits, intracortical layers, basal ganglia circuits, while networks are more nebulous in terms of circuit details, MTL networks, DMN networks, "executive control" network so I would use "networks" in the 2nd sentence, since it is used in the topic sentence of the paragraph.

Methods

Minor: Line 460 (i.e., delay conditioning). We did not undertake responses for establishment of a conditioned response is this is arguably independent of conscious contingency awareness48,49.
Line 461: this should be "conditioned response as this is" not response is this is!

Results

Moderate to Major: Figure 2: The figure legends are quite sparse and need greater explication. It seems obvious, but not explicit, that the purplish distribution is the CS+ neg (amygdala and HPC conditioning phase) while the green distribution is the CS+ neutral. If I am correct that should be explicit.

Actually, it is not clear to me what the green and purplish distribution actually represents as there is no Y axis label (the text refers to theta and gamma activity, but I suppose this is amplitude, or it could be some deviation from a mean). Last, I presume the X axis is time, but I could again be wrong and it is not clear why the time scales (if X is a time-scale) is different across distinct figures.

I went to the methods section for clarification, but there are no details on the basic analyses associated with Fig 2, as the methods go from paradigm to connectivity analyses, seemingly skipping the methods of Fig 2 and perhaps Fig 3.

As importantly, and I'm guessing here that the top traces, density maps by time/frequency are individual traces? Or are they grouped traces. There is a dotted line on these maps which could be the onset of fixation, or more likely the onset of the CS+ stimuli? Which perhaps is time 0 on time axis: this is not explicated in the figure legend. It is not at all clear why the time axis starts at -2.0 seconds, while the next interval is 0.5 seconds. I realize this may be related to the jittering of the fixation period between 1000-1500ms, but I just guessing as the figure legend is exceedingly sparse.

Finally on Fig 2 , I presume all subjects regardless of whether they had just amygdala, just hpc or both were tested across CS+ negative vs CS+ neutral conditions, so were any analyses done within subjects on the variation in theta/gamma/alpha across CS+ neg/neutral condition?

Moderate: Figure 3. I like the presentation in Fig 3B that well there is a high correlation between amydala and hippocampal broadband activity that is quite independent of conditioning. But I'm not sure there is any mention of this data in the discussion or any significant comments in the results presentation (I may have missed!). What did the authors expect and/or make of these data?

Discussion

Moderate: Line 322 "Gamma activity findings align with prior animal studies that associate gamma oscillations with local neuronal processing during fear conditioning31.

Gamma in the hippocampus in an inherent product of local circuits dynamics driven by rhythmic theta inputs (see Bragin … Buzsaki, 1995; Chrobak & Buzsaki, 1995). Basically, the rhythmic excitatory inputs drive local interneurons to discharge at gamma frequency regardless of the ongoing behavior (property of circuit/network) with minimal, if not any relation to some abstract/operationally defined psychological or "conditioning" phenomenon eg, "fear conditioning".

I guess what I'm objecting too is the specific association of increased gamma with "fear conditioning", it just seems inappropriate and misleading to those who may not understand the basic circuit dynamics.

Moderate: Line 324 "Interestingly, the reversal of these spectral patterns during extinction" should explicitly define what was reversed so "interestingly, the reversal evidencing….be explicit…"
In this paragraph or elsewhere, the authors may want, to explicate that "alpha" may simply reflect a faster frequency theta LFP. Line 206 reads "greater high theta-alpha (6-8 Hz)".

Personally, I'm more comfortable with describing 6-8 as high theta, or high theta-alpha, than just defining it as "alpha". I would expect that a CSD analysis of such LFPs in human HPC would not reveal significantly different sinks/sources for the generation of the faster frequency "high theta-alpha" LFPS, but alas I don't know of any human CSD analyses….the authors however may want to review a fairly recent human intracranial study by [Víctor J. López-Madrona, Agnès Trébuchon, Ioana Mindruta, Emmanuel J. Barbeau, Andrei Barborica, Costi Pistol, Irina Oane, F. Xavier Alario and Christian G. Bénar eNeuro 21 March 2024, 11 (4) ENEURO.0183-23.2023; https://doi.org/10.1523/ENEURO.0183-23.2023] for relevant information on such.

My main point is that in different places in the manuscript, 6-8 Hz seems to high theta or high theta-alpha or in this paragraph just alpha, so reviewing and explicitly defining would be useful.

Minor: Last, I came across the following manuscript that may be relevant to cite a the authors discretion.

[Theta–Alpha Connectivity in the Hippocampal–Entorhinal Circuit Predicts Working Memory Load, Jin Li, Dan Cao, Shan Yu, Haiyan Wang, Lukas Imbach, Lennart Stieglitz, Johannes Sarnthein and Tianzi Jiang Journal of Neuroscience 24 January 2024, 44 (4) e0398232023; https://doi.org/10.1523/JNEUROSCI.0398-23.2023].

Reviewer #2

(Remarks to the Author)
This manuscript "Adaptive shifts in amygdala–hippocampal theta coupling1
govern aversive learning and extinction", by Sonkusare et al, presents a well-conceived and technically rigorous study investigating the spectral and directional dynamics of human amygdala–hippocampal interactions during aversive learning and extinction using intracranial EEG. The work is timely, methodologically sound, and addresses an important gap in the literature by providing direct, high-resolution evidence for frequency-specific reconfiguration of emotional memory circuits in humans.
The authors employ a conditioning-extinction paradigm with IAPS stimuli and intracranial recordings from neurosurgical patients to interrogate local and interregional oscillatory activity. The two theta sub-bands herein analyzed as channels for information transfer and processing have few precedents in the literature (Kramis 1975, Goyal and Jacobs 2020, Remondes and Wilson 2013), as do the theta-nested gamma rhythms also studied here. In both cases, cross-frequency oscillatory interactions as substrates of learning and memory encoding, retrieval and extinction, remain understudied. This manuscript provides a fundamental contribution by reporting a double dissociation in theta sub-bands (3–5 Hz vs. 6–8 Hz), with distinct directional coupling across learning phases. These findings are well-supported by both phase connectivity metrics and computational modeling.
Major strengths:
1. Innovative use of intracranial EEG: The authors take advantage of the high temporal and spatial resolution of iEEG to provide insight into fast-timescale dynamics in amygdala–hippocampal communication, which are not accessible via non-invasive methods.
2. Insight into emotional learning: The frequency-specific findings—particularly the bidirectional and valence-dependent modulation of theta sub-bands—represent a significant advance in our understanding of the neurophysiological basis of affective flexibility in humans.
3. Strong analytical framework: The combination of power analyses, phase connectivity, transfer entropy, and DCM provides a comprehensive and convergent account of the data. Model selection is appropriately used to support directional inferences.
4. Translational relevance: By isolating oscillatory mechanisms specific to aversive learning and extinction, the work lays conceptual groundwork for spectral-targeted neuromodulation in disorders such as PTSD.
Limitations and areas for clarification:
1. Generalizability of findings: As acknowledged by the authors, participants were epilepsy patients undergoing clinical monitoring. While the authors excluded epileptogenic zones and controlled for noise, this sample limits the generalizability of findings. Further justification or contextualization of how these results may translate to neurotypical populations would strengthen the manuscript.
2. Anatomical specificity: The inability to resolve subnuclei within the amygdala and hippocampus is a limitation. If possible, anatomical localization of electrodes could be better visualized (e.g., in supplemental figures), and the potential impact of heterogeneous subregions further discussed.
3. Stimulus potency: The use of IAPS images as unconditioned stimuli may not fully capture the intensity of aversive experiences. While this choice has advantages in ecological validity and widespread applicability, it may limit the engagement of certain fear circuits. This tradeoff could be more explicitly discussed.
4. Definition of theta sub-bands: The division of the theta range into low (3–5 Hz) and high (6–8 Hz) sub-bands is central to the findings. It would be helpful to clarify whether this division aligned with prior human and maybe rodent literature, and support it with data-driven assessments resulting from the analysis of raw data from actual behavioral epochs.
5. Participant-level variability: Given the small sample size, it would be informative to report how consistent the observed effects were across participants. Any individual variability, lateralization effects, or correlations with behavioral measures (e.g., valence ratings) would enrich interpretation.
Minor comments:
• The methods could benefit from a more detailed description of trial structure and temporal alignment between stimuli and signal epochs.
• The discussion would be strengthened by further integration with human neuroimaging findings (e.g., fMRI studies of extinction networks).
• Clarify whether sex or age differences were assessed or controlled for, as these may influence emotional memory processing.
Summary:
This is an important and well-executed stud that makes a valuable contribution to our understanding of the neural dynamics underlying emotional learning and extinction. While some limitations remain—particularly regarding population

generalizability and anatomical resolution—the overall data quality, analytical rigor, and conceptual clarity merit publication.

Reviewer #3

(Remarks to the Author)
The manuscript titled ‘Adaptive shifts in amygdala–hippocampal theta coupling govern aversive learning and extinction’ addresses an important topic in the field. The study investigates the spectral and directional dynamics of amygdala–hippocampal interactions during aversive learning and extinction paradigm in humans. Using intracranial EEG (iEEG) from patients undergoing clinical monitoring for epilepsy, the authors conducted a two-day conditioning-extinction task with emotionally valenced stimuli. They identified frequency-specific changes in power and connectivity, notably distinguishing low (3–5 Hz) and high (6–8 Hz) theta sub-bands and their dynamic directionality. The results suggest a bidirectional and frequency-specific interplay between the amygdala and hippocampus underpinning emotional memory regulation.

Overall, the study leverages intracranial recordings, offering high temporal and spatial resolution not available in non-invasive modalities. This is a rare and valuable dataset in human neuroscience. The authors employ a comprehensive suite of analyses including ERSPs, wPLI, Transfer Entropy, Dynamic Causal Modelling (DCM), and phase-amplitude coupling (PAC), providing a multifaceted characterization of connectivity. The double dissociation in low and high theta band connectivity, with corresponding directional shifts, offers a novel insight into the dynamic regulation of emotional learning and extinction. The correlation of amygdalar theta activity with valence ratings adds interpretability, linking neural findings to subjective emotional appraisal. The discussion links the findings well with existing literature in both human and animal models, advancing the conceptual framework around emotional memory and extinction.

While the manuscript presents important and novel findings, major revisions are necessary to address limitations in generalisability, clarify methodological choices, and improve the presentation of results and figures. If these issues are addressed, the work could make a significant contribution to the field of human affective neuroscience.

Major Concerns
• While the authors acknowledge this, the epilepsy patient population may exhibit atypical neurodynamics, even when excluding epileptogenic zones. Future studies in healthy individuals (e.g., via MEG or advanced fMRI) are needed to replicate findings.
• No explicit behavioral indices of learning were collected during the conditioning/extinction phases, which limits interpretation of learning success on a trial-by-trial basis.
• The use of IAPS images, while ecologically valid, may produce less robust conditioning than electric shock or airpuff paradigms. This could affect the magnitude and specificity of neural responses.
• Although 30 patients were included, only 14 had simultaneous amygdala-hippocampus recordings, limiting some connectivity analyses' robustness.

Minor Issues:
Many grammatical and typographical errors should be corrected. Here are some examples:
Figure 1:
Panel A: The color-coded dots are very small and difficult to visualize. Improvements are necessary, as the current presentation may not be clear or meaningful to readers. Additionally, the two plots in the middle and on the right—representing the amygdala and hippocampus—are confusing without more detailed descriptions in the figure legend. If the two plots represent different subjects, using separate color bars for each plot would make interpretation easier for readers.

Line 132: Please define the abbreviation 'ERSP' at its first occurrence in the text.

Lines 132–153: Is there any reason to use unmatched closing parentheses (i.e., missing opening parentheses)? The presentation of the statistical results appears unusual due to the use of unmatched closing parentheses.

Figure 2 is of poor quality in terms of text font size, statistical plots, and figure legends, making it difficult to effectively convey the experimental results.

Result section:
The first part of the Results section, which includes ‘Amygdala ERSPs’ and ‘Hippocampal ERSPs’, is not well organized and contains some confusing subheadings.

Line 146: Fig. B top: missing figure number

Figure 4 appears to be missing, or incorrect figure numbers may have been used for Figures 5 and 6.

Line 207 (also line 213): Incorrect figure citation for wPLI — "(Fig. 5B left)" should be "(Fig. 5A, left)." The panel B presented Transfer Entropy (TE). However, the figure number itself also appears to be incorrect.

Figure 5A: I am wondering whether the data traces were smoothed, as this could significantly alter the observed patterns of wPLI changes.

Figure 5B: In the top portion of each plot, the y-axis should be labeled to indicate whether it represents amplitude or an index value. Additionally, abbreviations such as 'cond' and 'ext' should be defined in the figure legend to ensure the figure is self-explanatory.

Figure 6: Legends.
More clarity is needed when using terms such as negative, neutral, conditioning, and extinction, whether used individually or in combination. For example, the sentence 'Bottom row shows the PAC for phase of amygdala modulating amplitude of hippocampus activity contrasts between negative (bottom left) and neutral (bottom right) conditioning and extinction' is quite confusing and should be rewritten for better understanding.
The figure should include brief statistical results, along with appropriate labeling and descriptions for each part. The text "Phase Amplitude Coupling" in the top-left corner of the figure is unclear—it's not evident whether it refers to the left column specifically or the entire figure. This should be revised for clarity or removed altogether.

Line 502: incorrect reference citation style
……

Reviewer #4

(Remarks to the Author)
The present study investigated the neural circuit mechanisms underlying learning and extinction in the human brain. The present work observed increases in theta and gamma band activities in the amygdala during conditioning for emotionally arousing stimuli (IAPS images as the US) and subsequent reversal of these spectral patterns during extinction. Furthermore, the results suggest frequency-specific oscillatory dynamics within the amygdala-hippocampal circuit during emotional conditioning and extinction. More specifically, low theta dynamics dominated amygdala-hippocampal connectivity during learning, while high theta dynamics dominated hippocampal-amygdala connectivity during extinction. These results provide novel evidence that distinct frequency bands support adaptive emotional behavior. The manuscript is well written and clear. It was co-reviewed by a principal investigator and a postdoctoral fellow. We offer suggestions for areas where the paper can be improved below.

Introduction

• The authors do an excellent job of describing the background literature relevant to their study, but do not lay out their specific hypotheses based on this prior literature. More information on this study's specific hypotheses would be helpful.

Methods

• It would be helpful to know more details about the amygdala localizations. Prior work suggests the BLA is essential for the initial learning and consolidation of fear memories. However, the central nucleus is critical for orchestrating the expression of the fear response. Are the authors able to isolate the different contributions of the BLA vs. the CeA to the oscillatory responses? The authors mention in the limitations sections that functionality of each subnuclei cannot be differentiated with these recordings, however, many labs have used subnuclei segmentations in intracranial epilepsy patients to determine the contributions of different nuclei to different cognitive processes.
• Was the Extinction procedure always conducted the subsequent day after Conditioning? Or did the delay vary for some patients? It would be great to list this somewhere.
• The Paradigm section in the Methods says a triangle and a square were used for the CS. However, Figure 1 depicts a triangle and a circle.
• The Paradigm section in the Methods says the intertrial interval consisted of a white fixation cross on a black background, however, in Figure 1 there is a black fixation cross on a white background.
• The Paradigm section in the Methods doesn't detail the procedure used for the Extinction phase of the task.
• For the dynamic causal modelling/phase coupling analyses, it would be helpful to know whether ipsilateral amygdala-hippocampus interactions were analyzed or contralateral in some cases. Studies suggest some subregions of the amygdala (like the BLA) only project to the ipsilateral hippocampus, so it would be unclear how one would interpret phase coupling interactions between the amygdala and the contralateral hippocampus, for example.

Results

• You show greater amygdala theta and gamma activity to negative compared to neutral CS during the learning/conditioning phase, however, what does this theta/gamma activity look like across the 45 conditioning trials. Is this a pattern that only emerges towards the end of the learning? Or is it present from the first few trials?
o Similarly, for the extinction phase you see this trend reverse (reduced amygdala theta/gamma activity to negative CS). Does it take several trials for this reversal to occur? Is it most apparent towards the end of the 60 extinction trials?
• Are these effects unique to the amygdala/hippocampus? Do you see this reversal of activity between conditioning and extinction in other regions that we know are important for these kinds of tasks? i.e. ACC or prefrontal cortex?
• Are these amygdala and hippocampal ERSP effects during conditioning unique to the CS? Or do they carry over to the US (IAPS stimuli presentation period)? i.e. in the instances where you see greater amygdala theta and gamma activity to the

negative CS compared to the neutral CS, do you also see greater amygdala theta and gamma activity to the subsequent US?
• Lines 145-153 detailing the hippocampal ERSPs results note the same effect for learning and extinction (greater alpha and gamma activity to negative CS). However, the heading for this section mentions a shift in activity from learning to extinction involving theta, implying there is a difference in what is happening during learning vs. extinction. There is no mention of theta in the written results for the extinction phase here.

Figures

• The Figure 1 caption doesn't include any information about the Extinction procedure that's depicted.
• The Figure 3A caption doesn't match what is stated in the text. The figure legend says a greater difference between neutral and negative valence is associated with smaller theta activity. The text says a larger neutral-negative valence difference is associated with greater theta power.
• The Figure 3B caption mentions CS negative on top and CS neutral on bottom, but the figure itself depicts conditioning on the top and extinction on the bottom with no CS negative or CS neutral depictions. Is the red vs. blue trace in the HFBA plot the CS negative vs. neutral?
• In Figure 6, the PAC illustrations to the left imply that this PAC was observed in both the negative and neutral conditions. However, your study did not find PAC for phase of amygdala modulating amplitude of hippocampus in the neutral condition. I'm wondering if it may be clearer to only include these PAC pink and blue illustrations under the appropriate conditions where you observed significant PAC?

Discussion

• Line 316 mentions theta oscillations in the amygdala are linked to the encoding of emotional salience, however the Seidenbecher 2003 citation listed focuses on theta during memory retrieval. Their focus was on analyzing theta in animals already conditioned.
• Line 319-320 mentions that theta oscillations encode learned emotional associations, not simply valence. However, can the authors be certain that the CS+ hasn't taken on negative valence properties over the course of conditioning? We would argue that theta oscillations could still simply be encoding valence because the CS+ has taken on properties of unpleasantness. In general, this sentence can be worded differently to reflect the possibility that theta oscillations encode both stimulus valence and emotional associations, without suggesting the present study can tease those things apart for certain.
• Line 327 highlights the possibility that the amygdala is involved in extinction and the "unlearning" of the association. However, there is also evidence that extinction instead reflects the new learning of a new association rather than unlearning. It would be helpful to speak to this idea and how your findings involving the amygdala and hippocampus during conditioning vs. extinction reconcile these two schools of thought on whether extinction is reversal of learning/unlearning vs. learning a new association. (See this review from the Phelps group: https://pmc.ncbi.nlm.nih.gov/articles/PMC4598943/)
• In lines 328-338 the authors discuss theta-gamma dynamics during early and late conditioning/extinction. It would be great if the authors could clarify whether this in early/late in the context of trials across time or early/late on a trial-averaged basis looking at early vs. late with respect to stimulus onset.
• Citation needed in line 358 for "amygdala's role in affective salience signals"
• In lines 365-370 it could be clearer how the DCM effects differed between conditioning vs. extinction. The way this section is currently written it sounds like the authors are saying negative conditioning and extinction showed the same effects. Whereas in the results sections it's clear that these directionality and frequency shifts are different between conditioning and extinction.

Limitations and conclusion

• The authors need to be transparent about how the DCM analysis wasn't run for the neutral conditioning and extinction (due to computational demands), so these system flexibility shifts in frequency effects may not be unique to encoding threat and may also be apparent in the neutral condition.
• In line 399 the authors mention their study provides evidence of the mechanisms of how the brain "suppresses emotional memories". The suppression of emotional information is controversial in the learning and memory literature and the authors should reword this accordingly. (Something more appropriate would be "....we offer a mechanistic account of how the brain flexibly encodes emotional information and subsequently updates emotional associations.")
• A brief analysis of prefrontal regions is within the scope of the present study. The authors have prefrontal coverage in these patients and highlight the role of prefrontal structures in this kind of learning/extinction. It would be helpful to know whether the observed effects are unique to the MTL or are also seen in regions like the ACC and OFC.
• Future directions for how these novel findings might be used in the context of PTSD or other anxiety disorders would be helpful.

Reviewer #5

(Remarks to the Author)
I co-reviewed this manuscript with one of the reviewers who provided the listed reports. This is part of the Nature

Communications initiative to facilitate training in peer review and to provide appropriate recognition for Early Career Researchers who co-review manuscripts.

Version 1:

Reviewer comments:

Reviewer #1

(Remarks to the Author)
Sonkusare and colleagues report on the dynamics of amygdala-hippocampal theta coupling during aversion learning and extinction via recording in human epileptic patients during an aversive and neutral conditioning paradigm and subsequent extinction.

The authors have been very thorough in responding to all of the prior critiques.

Reviewer #2

(Remarks to the Author)
The authors have appropriately addressed my concerns. The manuscript is ready to be published.

Reviewer #3

(Remarks to the Author)
This is a revised version of the manuscript. My questions have been addressed.

Reviewer #4

(Remarks to the Author)
The authors have done an excellent job addressing the concerns of the reviewers, but some additional minor revisions could be made.

Introduction
The authors now state hypotheses at the end of introduction section, but we think there needs to be some added clarity.

For example, they say "expected for theta connectivity based on our previous amygdala neural recordings". However, this is fairly vague. Did they expect strong connectivity between the amygdala and hippocampus?

They also state "anticipated task induced condition differences in the low frequency range...". But did they expect a difference between learning vs. extinction? If that's what they mean by "condition", then we think that can be more clearly stated.

Methods
To our prior point on DCM modeling, the author's response to this comment is excellent. However, in the Methods section on page 14 the red text includes "...as the reviewer has pointed out."

We think this phrase about the reviewer can be removed.

Results
The authors have now run the DCM analysis for the neutral condition, however we think there needs to be some added clarity about the conclusions of this analysis. In the added red text in the Results on page 7 it's not clear what the conclusion is from this added analyses. A statement about how the results are different (or the same) between the DCM for the negative vs. neutral conditions would be helpful. Especially if the authors are trying to make the claim that there is something fundamentally different about the amygdala-hippocampus dynamics for negative vs. neutral condition.

Reviewer #5

(Remarks to the Author)
I co-reviewed this manuscript with one of the reviewers who provided the listed reports. This is part of the Nature Communications initiative to facilitate training in peer review and to provide appropriate recognition for Early Career Researchers who co-review manuscripts.

Open Access This Peer Review File is licensed under a Creative Commons Attribution 4.0 International License, which permits use, sharing, adaptation, distribution and reproduction in any medium or format, as long as you give appropriate credit to the original author(s) and the source, provide a link to the Creative Commons license, and indicate if changes were made.
In cases where reviewers are anonymous, credit should be given to 'Anonymous Referee' and the source.
The images or other third party material in this Peer Review File are included in the article's Creative Commons license, unless indicated otherwise in a credit line to the material. If material is not included in the article's Creative Commons license and your intended use is not permitted by statutory regulation or exceeds the permitted use, you will need to obtain permission directly from the copyright holder.
To view a copy of this license, visit https://creativecommons.org/licenses/by/4.0/

We thank the editor and reviewers' constructive suggestions and comments. Our responses are detailed below in dark blue. Relevant sections from the revised manuscript are quoted in italics with changes marked in red.

Reviewer #1 (Remarks to the Author):

Sonkusare and colleagues report on the dynamics of amygdala-hippocampal theta coupling during aversion learning and extinction via recording in human epileptic patients during an aversive and neutral conditioning paradigm and subsequent extinction.

The paper reads well and uses multiple analytic methods to examine LFP interactions across structures. The authors also do a nice job of referencing a considerable number of analytic/methods papers for examining LFP/EEG interactions across structures. The results generally support dynamic fluctuations in amygdala-hippocampal interactions as one might expect during such a learning task.

While I understand the focus on aversive conditioning, it is perhaps unfortunate that the authors did not include a positive CS+. The paper reads as if amygdala-hippocampal interactions largely support aversive conditioning, while invariably amygdala-hippocampal interactions support both +/- conditioning processes and the formation of post positive/negative emotional memories. Some comment on this somewhere in the manuscript may be appropriate.

Response: We thank the reviewer for this thoughtful comment. We agree that amygdala–hippocampal interactions are not exclusive to aversive learning, but more broadly support emotional learning and memory formation across both positive and negative valence. We have now clarified this point in the Discussion, explicitly noting that amygdala–hippocampal coupling supports emotional memory formation across valences, and that the observed effects should be interpreted as reflecting aversive learning within this broader functional framework. We also highlight future studies incorporating both positive and negative reinforcement will be essential for disentangling shared versus valence-specific dynamics within amygdala–hippocampal circuits.

Discussion Pg 8

"Amygdala–hippocampal coupling supports emotional memory formation across valences, and our results should be interpreted as reflecting aversive learning within this broader functional framework."

Limitations Pg 11

"Furthermore, incorporating both positive and negative valenced stimuli will be essential for disentangling shared versus valence-specific dynamics within amygdala–hippocampal circuits."

1) I have some minor to moderate suggestions for improvement as explicated below in order of occurrence. Generally, several/most of the figure legends could be significantly enhanced with additional explications as outlined below. Some of the figure legends are quite sparse on content.

Response: We thank the reviewer for the suggestions. We have now extensively revised the figures and all the captions with additional information for clarity.

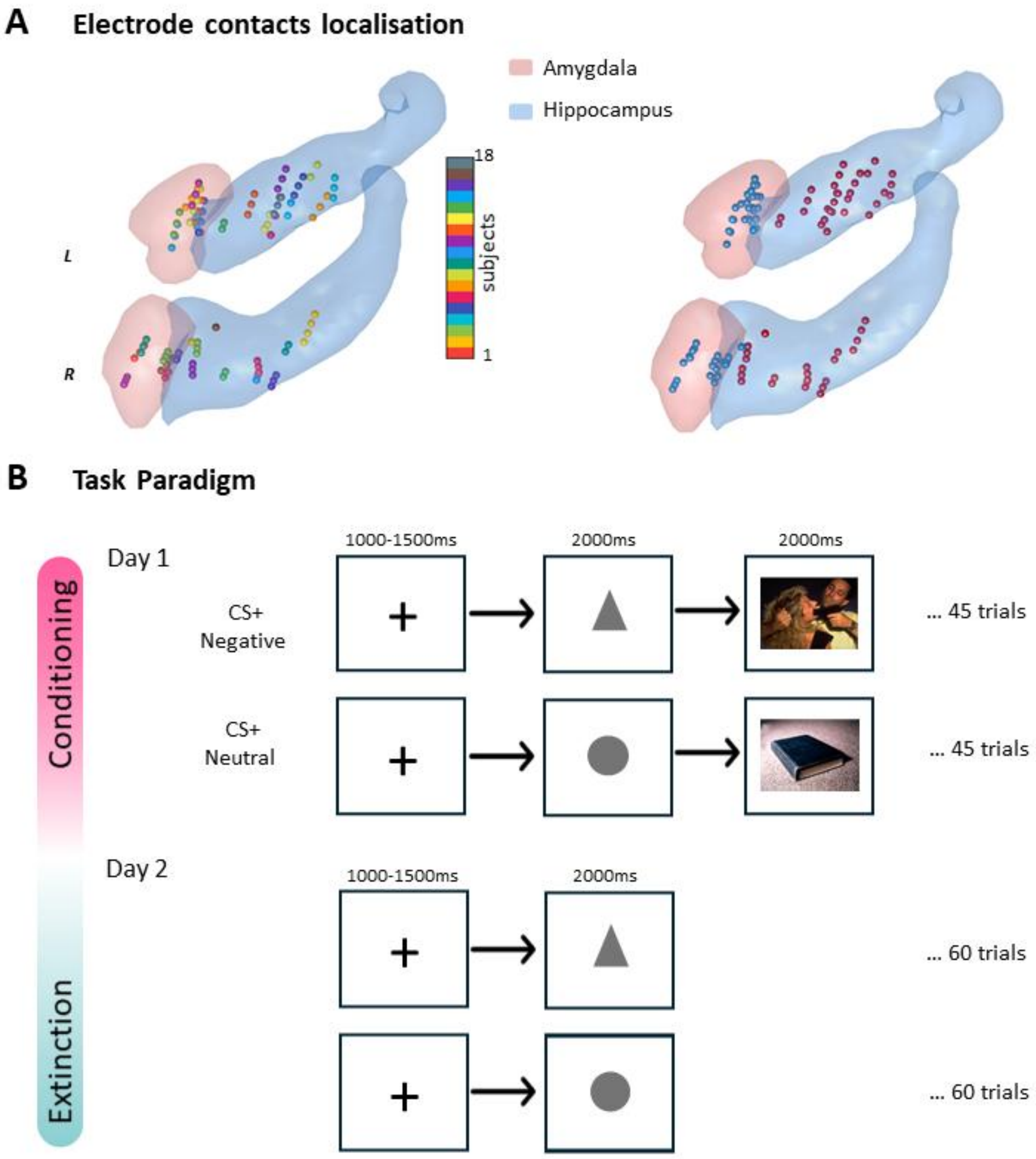

Figure 1. Localization of electrode contacts and task paradigm. A) Amygdala shown in pink and hippocampus shown in blue in standard MNI brain space (L=left side brain, R=right side brain). Each dot is an electrode contact colour coded corresponding to each patient (left). Amygdala electrode contacts shown in blue and hippocampus contacts shown in red (right) **B)** Conditioning and extinction task paradigm: The paradigm was conducted across two successive days in a clinical setting, with conditioning (pink) undertaken on Day 1 and extinction (teal) undertaken on Day 2. Conditioned stimuli (CS+) consisted of a triangle or a circle presented for 2000 ms, followed by presentation of the unconditioned stimulus (US) for 2000 ms. The triangle was paired with a negative US, comprising negatively valenced images, whereas the circle was paired with a neutral US, comprising neutrally valenced images selected from the International Affective Picture System (IAPS). Extinction procedure was carried on Day 2 which followed similar process as conditioning except negative and neutral images were not shown. A fixation cross preceded each CS presentation and was jittered between 1000–1500 ms.

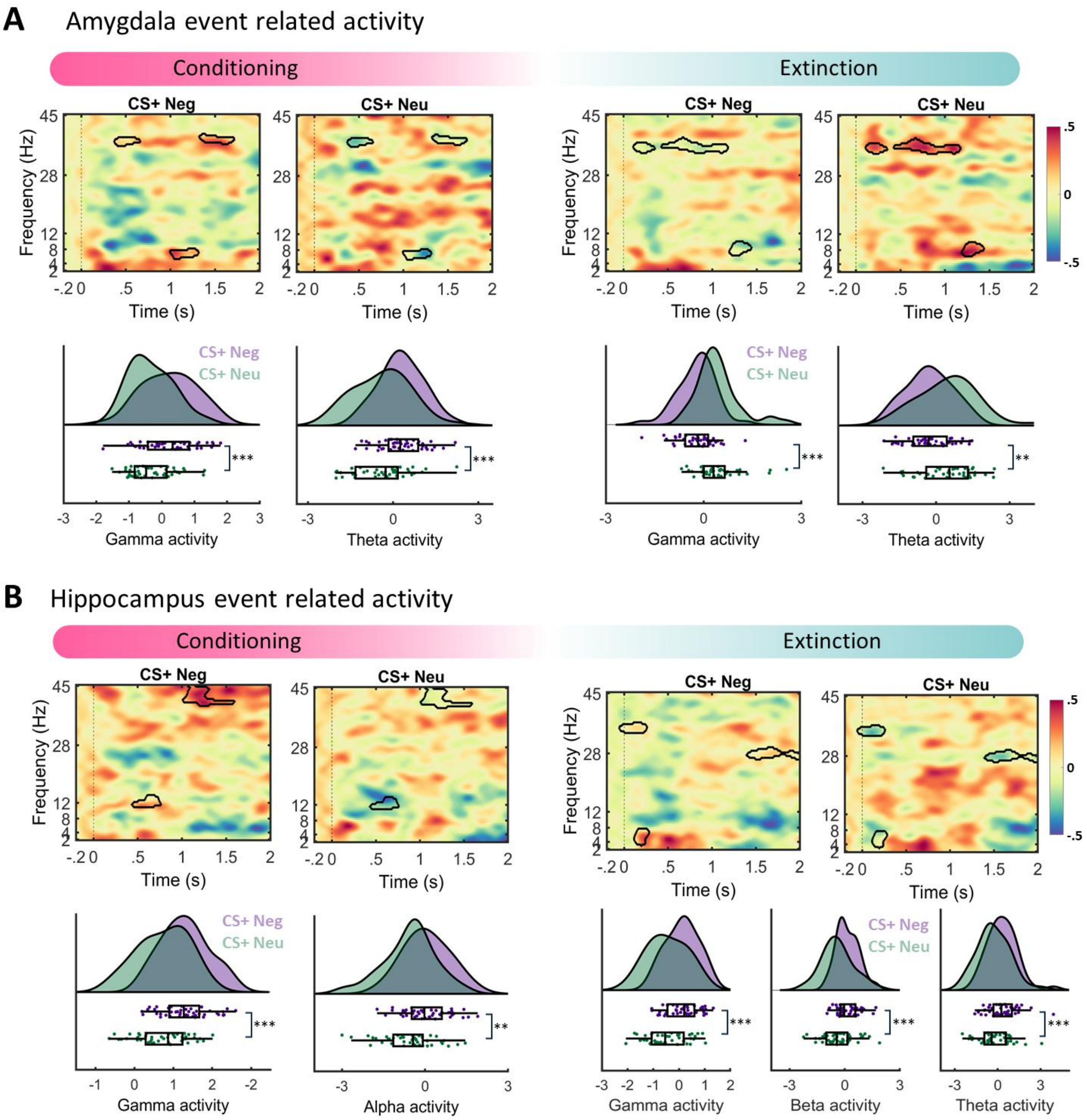

Figure 2. Task induced responses and condition differences. ***A)*** *Amygdala:* Grand-averaged event-related spectral perturbation (ERSP) maps during conditioning (left) and extinction (right) for CS+ Negative (CS+ Neg) and CS+ Neutral (CS+ Neu) stimuli. Vertical dotted line indicates the onset of stimuli. Warmer colours indicate task-related increases in spectral power, whereas cooler colours indicate power decreases relative to baseline. Black contours denote time–frequency clusters showing significant differences between CS+ Neg and CS+ Neu conditions, identified using permutation testing. Raincloud plots below the ERSP maps summarize mean spectral power extracted from the significant clusters. Purple distributions represent CS+ Neg trials and green distributions represent CS+ Neu trials. Y-axis in these raincloud plots depict the distribution density and has no independent interpretive scale. Notably, theta- and gamma-band responses show a reversal in relative activity during extinction compared with conditioning. ***B)*** *Hippocampus:* Similar grand averaged ERSP maps as for amygdala during conditioning (left) and extinction (right). Raincloud plots below the ERSP maps summarize mean spectral power extracted from the significant clusters. Purple distributions represent CS+ Neg trials and green distributions represent CS+ Neu trials. Y-axis in these raincloud plots depict the distribution density and has no independent interpretive scale.

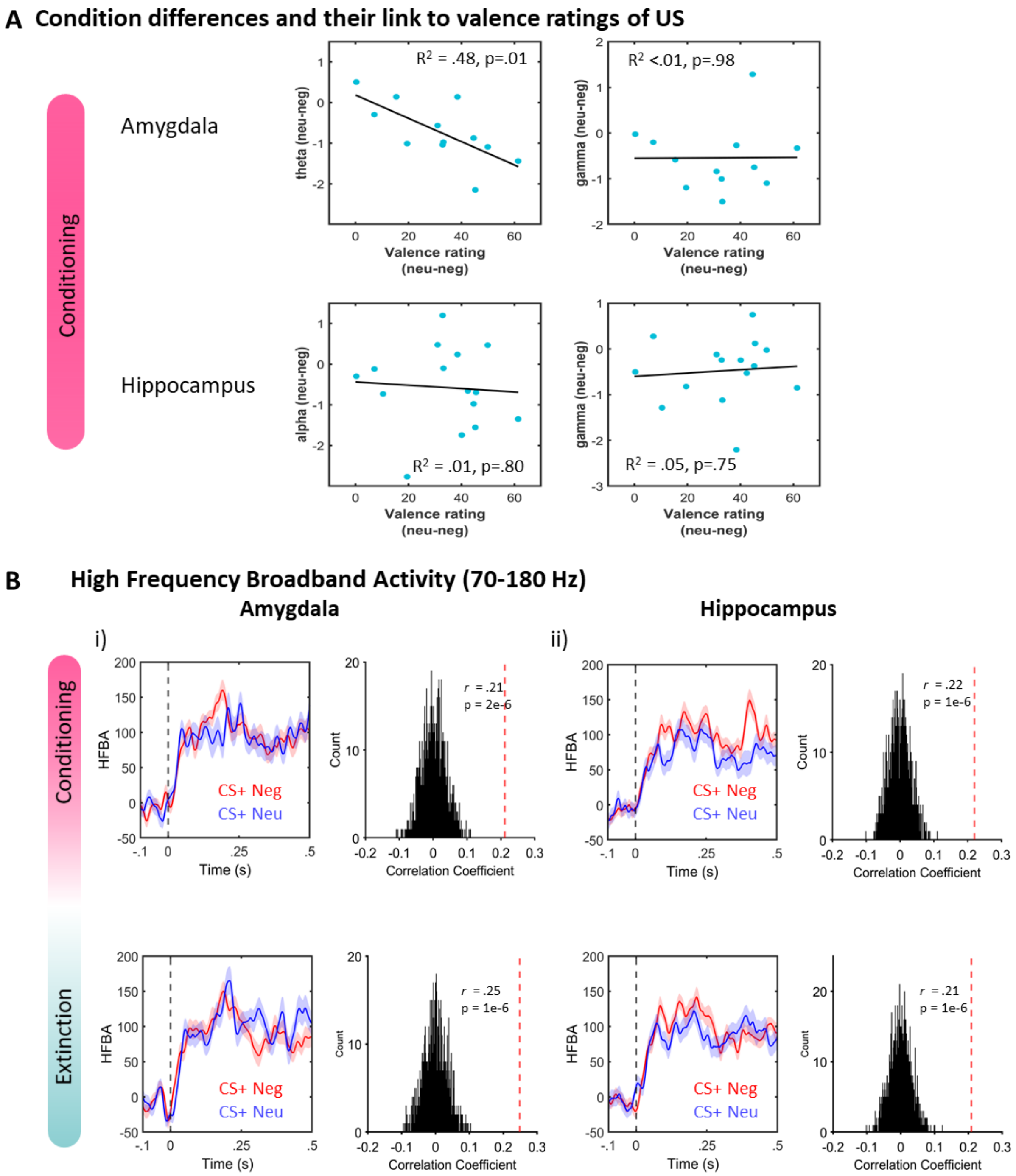

Figure 3: Event related condition differences during conditioning and their association with valence ratings of unconditioned stimuli and characterisation of high frequency activity profile. ***A)*** *Frequency-specific activity and valence ratings*: Mean event-related activity for each frequency band cluster during the conditioning phase (Fig. 2) was computed separately for each participant. For both the amygdala and hippocampus, frequency specific condition differences were calculated as CS+ Negative (Neg) minus CS+ Neutral (Neu). This resulted in theta- and gamma-band difference measures for the amygdala, and alpha- and gamma-band difference measures for the hippocampus. These were associated with the mean valence ratings of the negative and neutral unconditioned stimuli with Pearson's correlation corrected for multiple comparisons (see methods) (i) Amygdala: The mean theta-band difference between CS+ Negative and CS+ Neutral significantly linked to US valence ratings (left), such that larger differences in neutral versus negative valence ratings were associated with more negative neutral versus negative theta activity (greater CS+ Neg theta activity). In contrast,

gamma-band activity did not show a significant association with valence ratings (right) (ii) Hippocampus: Neither alpha-band (left) nor gamma-band (right) activity differences showed a significant association with valence ratings. ***B)*** *High frequency broadband activity (HFBA)* (i) Amygdala: HFBA during the conditioning phase for negative and neutral images (top) and for extinction phase (bottom) showing high similarity with adjacent plot showing statistically testing with permutation testing showing null distribution and correlation value in dashed vertical line (ii) Hippocampus: HFBA during the conditioning phase for negative and neutral images (top) and for extinction phase (bottom) showing high similarity with adjacent plot showing statistically testing with permutation testing showing null distribution and correlation value in dashed vertical line.

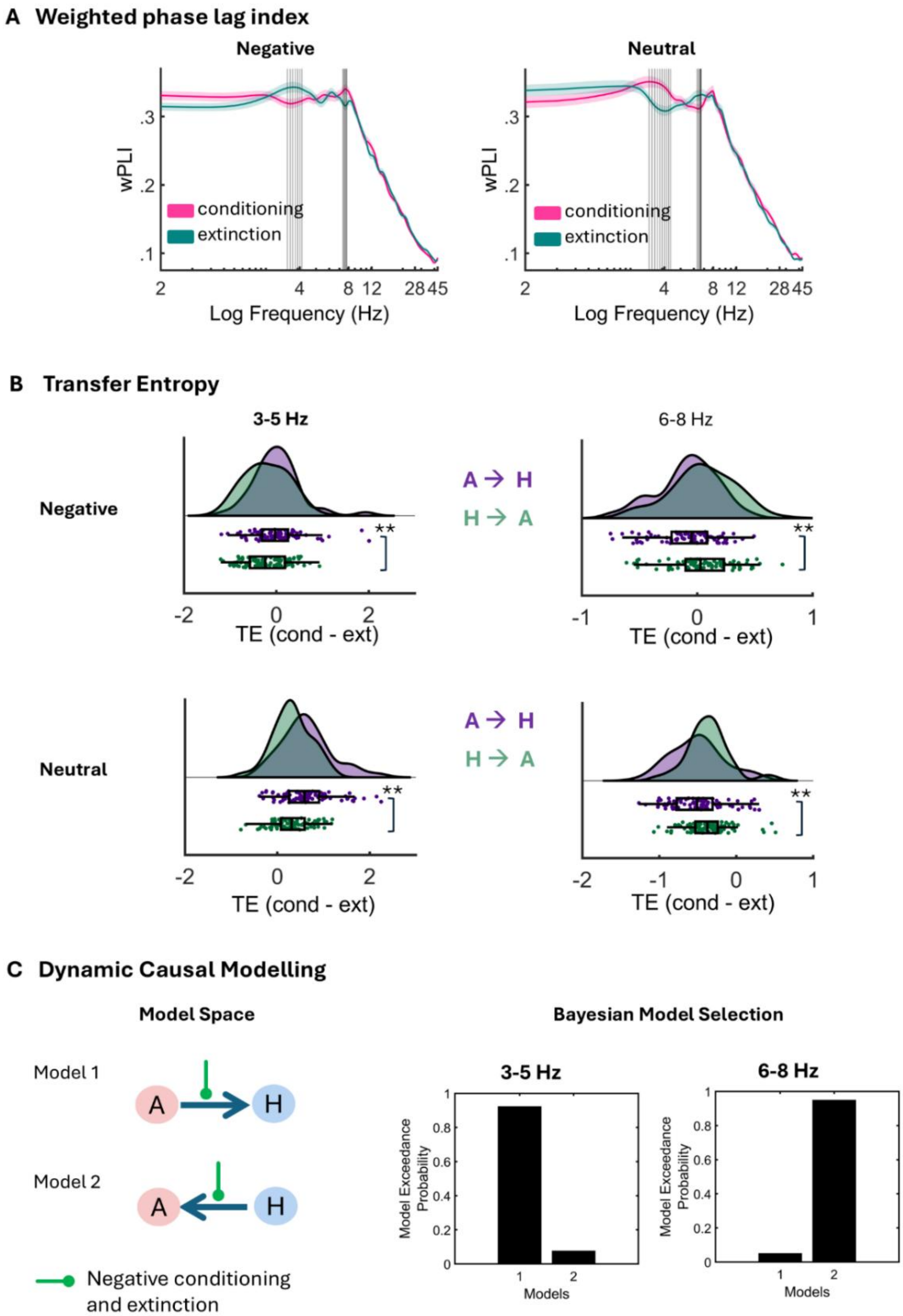

Figure 4: Amygdala-hippocampal connectivity. ***A)*** *Weighted phase lag index (wPLI):* connectivity differences in low theta (3-5 HZ) and high theta (6-8 Hz) were observed. Specifically, a double dissociation was found with lower low-theta and high theta connectivity in negative and neutral conditioning and extinction. Grey vertical lines show frequencies where significant differences were found with permutation testing (see methods). Shading indicates SEM ***B)*** *Transfer entropy (TE):* Directional information flow between the amygdala and hippocampus was quantified using transfer entropy in the low theta (3–5 Hz; left) and high theta (6–8 Hz; right) frequency bands during conditioning (cond) and extinction (ext) for CS+ Negative (CS+ Neg) and CS+ Neutral (CS+ Neu) stimuli. TE was computed for the amygdala → hippocampus and hippocampus → amygdala directions. Condition differences were calculated as CS+ Neg in conditioning minus CS+ Neg in extinction and CS+

Neu in conditioning minus CS+ Neu in extinction, in for each direction and shown as raincloud plots. Y-axis in these raincloud plots depict the distribution density and has no independent interpretive scale. Statistical analyses revealed predominant amygdala-to-hippocampus information flow in the low-theta range, whereas hippocampus-to-amygdala information flow dominated in the high-theta range, for both negative and neutral conditions. ***C)*** *Dynamic Causal Modelling:* For testing the effective connectivity in both 3-5Hz and 6-8Hz we employed a model space which comprised two models, with unidirectional connection from amygdala (A) to hippocampus (H) and from hippocampus to amygdala respectively and CS+ Negative conditioning and extinction task modulation effects influencing the connectivity. Bayesian model selection identified model 1 (amygdala to hippocampus) as possessing the highest exceedance probability (92.3%) for 3-5 Hz while for 6-8 Hz model 2 (hippocampus to amygdala) had the highest exceedance probability (94.9%).

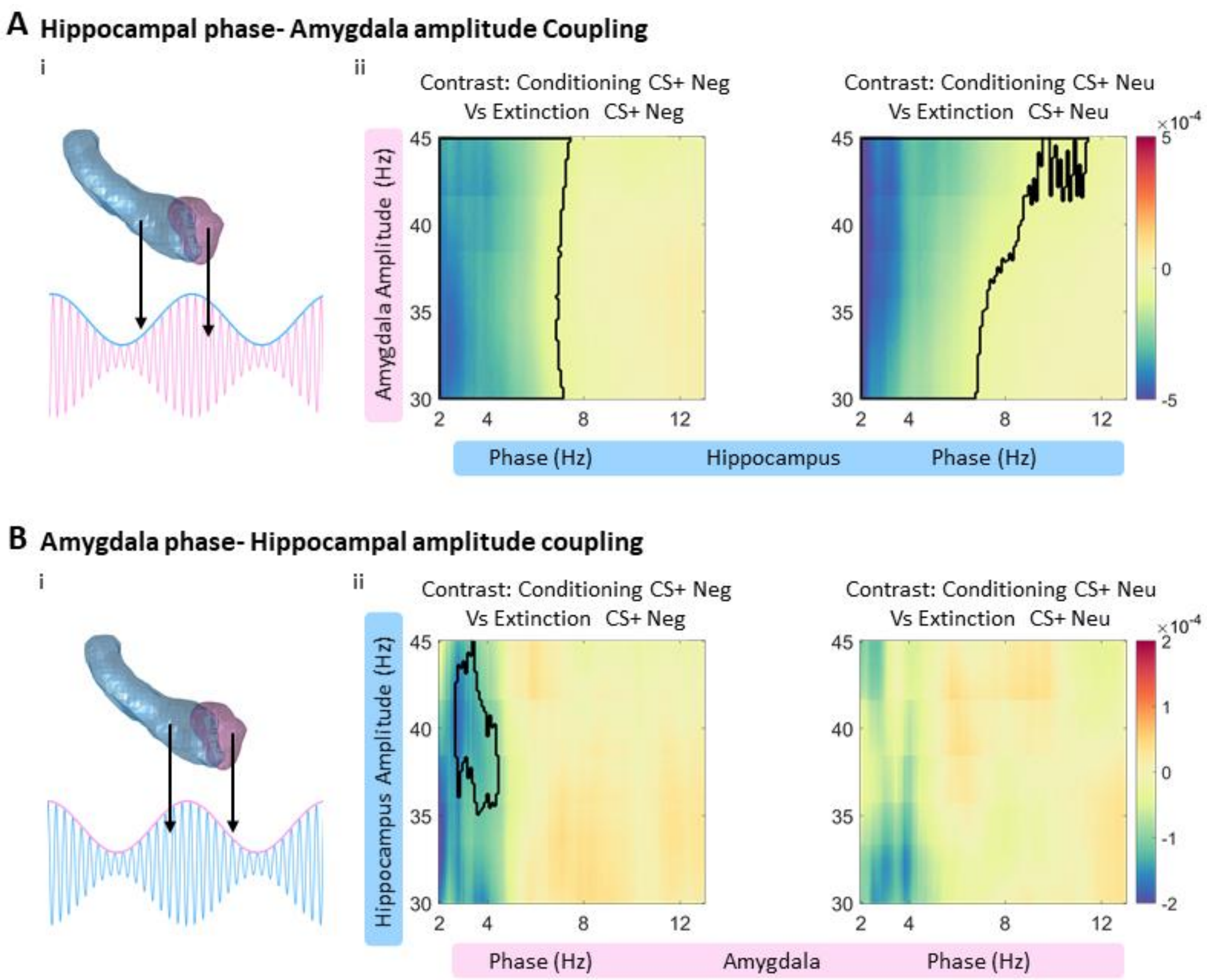

Figure 5: Phase–amplitude coupling (PAC) between the hippocampus and amygdala. *A)* *Hippocampal phase–amygdala amplitude coupling* (i) illustration of hippocampal low frequency phase shown in blue and amygdala frequency amplitude shown in pink ii) PAC comodulograms contrast between conditioning and extinction for CS+ Negative (left) and CS+ Neutral (right) stimuli. Black outlines denote clusters that reached statistically significant difference between the two conditions based on permutation testing. ***B)*** *Amygdala phase– Hippocampal amplitude coupling* (i) illustration of amygdala low frequency (2-12 Hz) phase shown in pink and hippocampal frequency amplitude (30-45 Hz) shown in blue ii) PAC comodulograms contrast between conditioning and extinction for CS+ Negative (left) and CS+ Neutral (right) stimuli. Black outlines denote clusters that reached statistically significant difference between the two conditions based on permutation testing (see methods).

Abstract

2) Minor: Lines 27-29 “Adaptive behaviour relies on the flexible encoding and suppression of aversive associations, yet the spectral and directional dynamics underlying amygdala–hippocampal interactions in humans remain poorly understood.

There is “adaptive behavior” that is quite independent of amygdala-hippocampal interactions.

Response: We have revised the sentence. Pg 2

“Adaptive behaviour relies on the flexible encoding and suppression of aversive associations often underpinned by amygdala-hippocampal interactions. Yet the spectral and directional dynamics underlying these interactions in humans remain poorly understood.”

Introduction

3) Minor: Line 61 “These processes are not only fundamental learning”

Add “support… “These process support not only fundamental learning”, as there is perhaps more to “fundamental learning” then “These processes”.

Response: We have incorporated this suggestion. Pg 3

“These processes support not only fundamental to learning but also central to understanding maladaptive responses in psychiatric disorders such as anxiety and post-traumatic stress disorder (PTSD)”

4) Moderate: Line 76+ “Nevertheless, the conditioning-extinction paradigm has established a distributed network……..prefrontal cortex. Within this circuit, the amygdala and hippocampus….

The paradigm didn’t “establish” the network, one might write the paradigm “revealed” or “provided insight into” a distributed network.

Next sentence uses “circuit” instead of “network”. There is not hard/fast definition of circuits vs networks, but I think of circuits as well-grounded, anatomical circuits, eg, EC-HPC microcircuits, intracortical layers, basal ganglia circuits, while networks are more nebulous in terms of circuit details, MTL networks, DMN networks, “executive control” network so I would use “networks” in the 2nd sentence, since it is used in the topic sentence of the paragraph.

Response: We have incorporated this suggestion and agree with the reviewer in terms of distinction between circuits and networks. Pg 3

“Nevertheless, the conditioning-extinction paradigm has revealed a distributed network supporting aversive learning and extinction, prominently involving the amygdala, hippocampus, insula, anterior cingulate cortex (ACC), and prefrontal cortex12-14. Within this network, the amygdala and hippocampus are central to the neural circuits governing aversive learning and extinction.”

Methods

5) Minor: Line 460 (i.e., delay conditioning). We did not undertake responses for establishment of a conditioned response is this is arguably independent of conscious contingency awareness48,49.

Line 461: this should be “conditioned response as this is” not response is this is!

Response: We have revised the sentences as per the suggestion.

Methods Pg 13

“We did not undertake responses for establishment of a conditioned response as this is arguably independent of conscious contingency awareness (Baeyens, Eelen et al. 1990, Walther and Nagengast 2006).”

Results

6) Moderate to Major: Figure 2: The figure legends are quite sparse and need greater explication. It seems obvious, but not explicit, that the purplish distribution is the CS+ neg (amygdala and HPC conditioning phase) while the green distribution is the CS+ neutral. If I am correct that should be explicit. Actually, it is not clear to me what the green and purplish distribution actually represents as there is no Y axis label (the text refers to theta and gamma activity, but I suppose this is amplitude, or it could be some deviation from a mean). Last, I presume the X axis is time, but I could again be wrong and it is not clear why the time scales (if X is a time-scale) is different across distinct figures.

Response: We have now edited the figure and the caption for clarity. In the raincloud plots we are using, the y-axis does not represent a measured quantity in the way a typical bar chart or scatter plot does; rather, it shows the density of the distribution (i.e., the shape of the cloud is a smoothed estimate of the probability distribution of the x-values). This is the same convention used in the example referenced (Allen, Poggiali et al. 2021), where the vertical axis simply spreads the density for visual clarity and does not carry a standalone unit or interpretable scale.

There is no meaningful y-axis label beyond “Density” — and even that is largely redundant in this context. The plot is meant to show the distribution of the x-values (e.g., how frequently different values occur), with the width of the cloud at any x-position corresponding to the relative density. We chose this format for exactly that reason: the focus is on comparing the

distributions along the x-axis, not on the absolute values of the y-axis. For clarity, we have added a note in the caption that the y-axis depicts the distribution density and has no independent interpretive scale.

"Y-axis depicts the distribution density and has no independent interpretive scale"

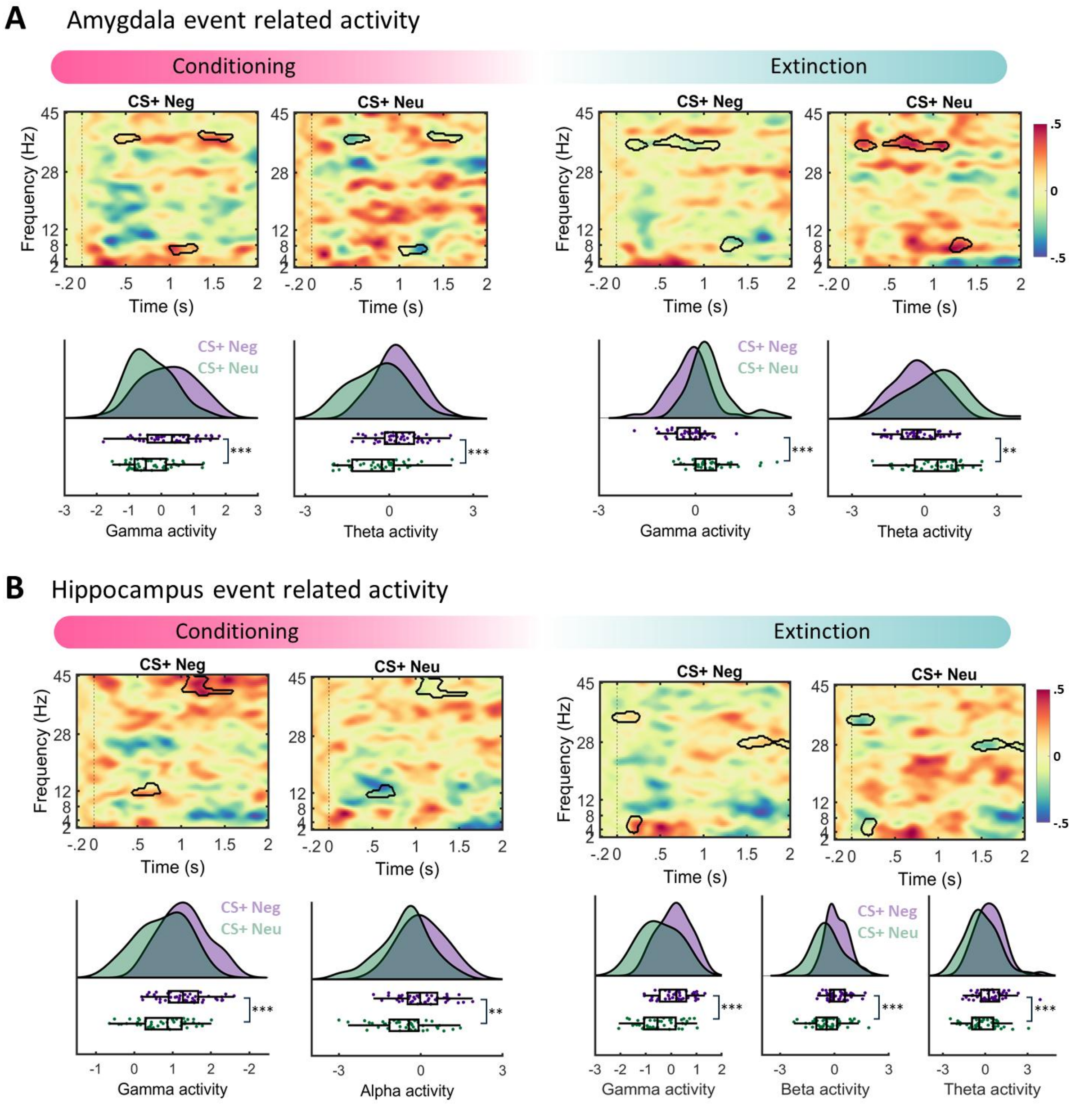

Figure 2. Task induced responses and condition differences. ***A)*** *Amygdala:* Grand-averaged event-related spectral perturbation (ERSP) maps during conditioning (left) and extinction (right) for CS+ Negative (CS+ Neg) and CS+ Neutral (CS+ Neu) stimuli. Vertical dotted line indicates the onset of stimuli. Warmer colours indicate task-related increases in spectral power, whereas cooler colours indicate power decreases relative to baseline. Black contours denote time–frequency clusters showing significant differences between CS+ Neg and CS+ Neu conditions, identified using permutation testing. Raincloud plots below the ERSP maps summarize mean spectral power extracted from the significant clusters. Purple distributions represent CS+ Neg trials and green distributions represent CS+ Neu trials.

Y-axis in these raincloud plots depict the distribution density and has no independent interpretive scale. Notably, theta- and gamma-band responses show a reversal in relative activity during extinction compared with conditioning. ***B) Hippocampus:*** Similar grand averaged ERSP maps as for amygdala during conditioning (left) and extinction (right). Raincloud plots below the ERSP maps summarize mean spectral power extracted from the significant clusters. Purple distributions represent CS+ Neg trials and green distributions represent CS+ Neu trials. Y-axis in these raincloud plots depict the distribution density and has no independent interpretive scale.

7) I went to the methods section for clarification, but there are no details on the basic analyses associated with Fig 2, as the methods go from paradigm to connectivity analyses, seemingly skipping the methods of Fig 2 and perhaps Fig 3.

As importantly, and I'm guessing here that the top traces, density maps by time/frequency are individual traces? Or are they grouped traces. There is a dotted line on these maps which could be the onset of fixation, or more likely the onset of the CS+ stimuli? Which perhaps is time 0 on time axis: this is not explicated in the figure legend. It is not at all clear why the time axis starts at -2.0 seconds, while the next interval is 0.5 seconds. I realize this may be related to the jittering of the fixation period between 1000-1500ms, but I just guessing as the figure legend is exceedingly sparse.

Finally on Fig 2 , I presume all subjects regardless of whether they had just amygdala, just hpc or both were tested across CS+ negative vs CS+ neutral conditions, so were any analyses done within subjects on the variation in theta/gamma/alpha across CS+ neg/neutral condition?

Response: We apologise that methods section did not mention the steps for these analyses. We have added this section. We have revised Fig 2 and expanded the caption which now clarifies the figure results. Specifically, the vertical dotted line indicates the onset of stimuli the baseline is of .2s (200ms). Analysis was undertaken on ERSP maps for each contact in amygdala and hippocampus to test the statistical difference between CS+ Neg and CS+ Neu in conditioning and similarly CS+ Neg and CS+ Neu in extinction. These details are mentioned in the Statistical Analysis section in the manuscript.

Methods Pg 13, 14

"Event Related Spectral Perturbation (ERSP)

Task induced changes were quantified and statistically tested with cluster-based permutations tests as employed in our previous work (Sonkusare, Ding et al. 2022, Sonkusare, Qiong et al. 2023). Specifically, event-related spectral perturbation (ERSP) quantifies changes in the power of ongoing neural oscillations across specific frequency bands and time points, relative to a baseline (Delorme and Makeig 2004). For our analyses, we used a 500 ms pre-stimulus period as baseline and examined a 2000 ms window following stimulus onset. ERSPs were computed using the fast Fourier transform (FFT) across 2–45 Hz in EEGLAB. These ERSP maps were first generated for individual electrode contacts (frequency × time) and then aggregated across

contacts within each region of interest to produce three-dimensional datasets (frequency × time × contacts).

High Frequency Broadband Activity (HFBA)

High-frequency activity (HFA) was estimated using high-frequency broadband amplitude (HFBA) following established approaches(Foster, Rangarajan et al. 2015). Preprocessed signals were then band-pass filtered between 70 and 180 Hz using consecutive 10-Hz frequency bands (70–80, 80–90, …, 170–180) with a zero-phase, two-pass finite impulse response (FIR) filter implemented in EEGLAB. The analytic amplitude of each band-limited signal was extracted using the Hilbert transform. These specific amplitudes were expressed as percentage change relative to this baseline (200-ms pre-stimulus) and averaged across 10 Hz frequency bands.

Statistical testing

To compare time frequency maps (ERSPs) between positive (relative to neutral) and negative conditions (relative to neutral), a nonparametric cluster-based permutation approach (Oostenveld, Fries et al. 2011) and has been previously employed by us(Sonkusare, Ding et al. 2022, Sonkusare, Qiong et al. 2023). This approach specifically corrects for multiple comparison issues in multidimensional data such as time-frequency decompositions or time domain data. It enables identification of clusters (time windows and/or frequency bins) with significant differences in the power changes induced by the presentation of pictures of different emotional valence. For permutations, the original paired samples for each region (condition ERSPs/connectivity metrics for each contact) were randomly permuted 1000 times such that each pair was maintained but its assignment to the condition (positive or negative) changed to create a null-hypothesis distribution. For ERSP maps, or wPLI or PAC maps, we applied strict significant level of $p < 0.01$ for cluster differences. We restricted the size of time frequency cluster >500 to find robust condition differences and to avoid small potentially meaningless clusters. Average power in the significant clusters were further compared using post-hoc paired t-tests with multiple comparison false discovery rate (FDR) correction (Benjamini and Hochberg 1995, Nichols and Hayasaka 2003). The condition differences were plotted raw time frequency plots (i.e., without subtraction of neutral condition) to appreciate early task induced activity and condition differences.

8) Moderate: Figure 3. I like the presentation in Fig 3B that well there is a high correlation between amygdala and hippocampal broadband activity that is quite independent of conditioning. But I'm not sure there is any mention of this data in the discussion or any significant comments in the results presentation (I may have missed!). What did the authors expect and/or make of these data?

Response: We have added a few sentences regarding this high frequency activity results in the Results section and the Discussion section. Specifically, this high frequency broadband activity is well validated marker for neuronal firing. Hence the similarity of this activity for amygdala and hippocampus shows that it is not selectively driven by stimulus negative or neutral CS+s but probably a general neuronal firing response. This high frequency activity interacts with low frequency oscillations to delineate the valence contexts.

Results Pg 5

"Similar High frequency broadband activity (HFBA) patterns in amygdala and hippocampus across negative and neutral conditions

We next examined whether HFBA—a proxy for neuronal firing—exhibited similar patterns in the amygdala and hippocampus. Permutation tests revealed strong correlations between negative and neutral conditioning and in extinction (amygdala - conditioning: $r = 0.21$, $p < 0.001$; extinction: $r = 0.25$, $p < 0.001$) (Fig. 3B left) and extinction phases (hippocampus - conditioning: $r = 0.22$, $p < 0.001$; extinction: $r = 0.21$, $p < 0.001$) (Fig. 3B right). These results suggest that these regions may engage a common local processing mechanism in response to stimulus presentation and not strictly valence-specific responses."

Discussion Pg 9

"The observation of similar high-frequency broadband (70–180 Hz) activity in the amygdala and hippocampus across negative and neutral conditions suggests that local neuronal processing in these regions is not selectively driven by stimulus valence. Such broadband activity may reflect general associative processes that operate alongside lower-frequency dynamics and interregional connectivity for valence-dependent learning and its updating during extinction."

9) Moderate: Line 322 "Gamma activity findings align with prior animal studies that associate gamma oscillations with local neuronal processing during fear conditioning31.

Gamma in the hippocampus in an inherent product of local circuits dynamics driven by rhythmic theta inputs (see Bragin …Buzsaki, 1995; Chrobak & Buzsaki, 1995). Basically, the rhythmic excitatory inputs drive local interneurons to discharge at gamma frequency regardless of the ongoing behavior (property of circuit/network) with minimal, if not any relation to some abstract/operationally defined psychological or "conditioning" phenomenon eg, "fear conditioning".

I guess what I'm objecting too is the specific association of increased gamma with "fear conditioning", it just seems inappropriate and misleading to those who may not understand the basic circuit dynamics.

Response: We thank the reviewer for this important point and agree that hippocampal gamma largely reflects intrinsic circuit dynamics driven by rhythmic theta inputs, as described in classical studies (Bragin, Jandó et al. 1995, Buzsáki and Chrobak 1995). Our intention was not to suggest that gamma oscillations are uniquely or causally generated by fear conditioning, but rather that gamma power was modulated during the presentation of conditioned stimuli. We have revised the text to clarify that observed gamma increases likely reflect theta-driven local processing within the hippocampus that is temporally linked to the CS, rather than implying that gamma itself is a direct marker of "fear conditioning." This phrasing better respects the underlying neurophysiology while maintaining the relevance of the observed modulation to the task.

Discussion Pg 8, 9

"Gamma activity modulation has been observed in prior animal studies during fear conditioning (Headley and Weinberger 2013)."

"Intricate links between gamma oscillations in the hippocampus arise from local circuit dynamics and are temporally organized by rhythmic theta inputs (Bragin, Jandó et al. 1995, Buzsáki and Chrobak 1995)."

10) Moderate: Line 324 "Interestingly, the reversal of these spectral patterns during extinction" should explicitly define what was reversed so "interestingly, the reversal evidencing….be explicit…"

Response: We have incorporated this suggestion. Pg 8

"Interestingly, the reversal of these spectral patterns during extinction i.e., reduced theta and gamma activity for negative relative to neutral trials in extinction, suggests a dynamic shift in amygdala engagement. This likely reflects its involvement in not only attenuating emotional responses to previously aversive stimuli but possibly also in the parallel unlearning of aversive associations."

11) In this paragraph or elsewhere, the authors may want, to explicate that "alpha" may simply reflect a faster frequency theta LFP. Line 206 reads "greater high theta-alpha (6-8 Hz)". Personally, I'm more comfortable with describing 6-8 as high theta, or high theta-alpha, than just defining it as "alpha". I would expect that a CSD analysis of such LFPs in human HPC would not reveal significantly different sinks/sources for the generation of the faster frequency "high theta-alpha" LFPS, but alas I don't know of any human CSD analyses….the authors however may want to review a fairly recent human intracranial study by [Víctor J. López-Madrona, Agnès Trébuchon, Ioana Mindruta, Emmanuel J. Barbeau, Andrei Barborica, Costi Pistol, Irina Oane, F. Xavier Alario and Christian G. Bénar eNeuro 21 March 2024, 11 (4) ENEURO.0183-23.2023; https://doi.org/10.1523/ENEURO.0183-23.2023] for relevant information on such.

My main point is that in different places in the manuscript, 6-8 Hz seems to high theta or high theta-alpha or in this paragraph just alpha, so reviewing and explicitly defining would be useful.

Response: We thank the reviewer for the insightful comment and the references. We have now replaced the words “theta-alpha” with just “high theta” throughout the manuscript to avoid any confusion.

12) Minor: Last, I came across the following manuscript that may be relevant to cite a the authors discretion.

[Theta–Alpha Connectivity in the Hippocampal–Entorhinal Circuit Predicts Working Memory Load, Jin Li, Dan Cao, Shan Yu, Haiyan Wang, Lukas Imbach, Lennart Stieglitz, Johannes Sarnthein and Tianzi Jiang Journal of Neuroscience 24 January 2024, 44 (4) e0398232023; https://doi.org/10.1523/JNEUROSCI.0398-23.2023].

Response: We thank the reviewer for this reference suggestion. We have incorporated the findings from this paper in the Introduction section.

Introduction Pg 4

“However, direct evidence for such frequency-resolved mechanisms in humans is limited, owing to the spatial and temporal constraints of non-invasive neuroimaging. Nevertheless, related intracranial EEG studies have reported frequency-specific connectivity, including theta–alpha coupling between the hippocampus and entorhinal cortex during memory (Li, Cao et al. 2024), and theta-band amygdala–prefrontal interactions19. (Chen, Tan et al. 2021).”

Reviewer #2 (Remarks to the Author):

This manuscript "Adaptive shifts in amygdala–hippocampal theta coupling govern aversive learning and extinction", by Sonkusare et al, presents a well-conceived and technically rigorous study investigating the spectral and directional dynamics of human amygdala–hippocampal interactions during aversive learning and extinction using intracranial EEG. The work is timely, methodologically sound, and addresses an important gap in the literature by providing direct, high-resolution evidence for frequency-specific reconfiguration of emotional memory circuits in humans.

The authors employ a conditioning-extinction paradigm with IAPS stimuli and intracranial recordings from neurosurgical patients to interrogate local and interregional oscillatory activity. The two theta sub-bands herein analyzed as channels for information transfer and processing have few precedents in the literature (Kramis 1975, Goyal and Jacobs 2020, Remondes and Wilson 2013), as do the theta-nested gamma rhythms also studied here. In

both cases, cross-frequency oscillatory interactions as substrates of learning and memory encoding, retrieval and extinction, remain understudied. This manuscript provides a fundamental contribution by reporting a double dissociation in theta sub-bands (3–5 Hz vs. 6–8 Hz), with distinct directional coupling across learning phases. These findings are well-supported by both phase connectivity metrics and computational modeling.

Major strengths:

1. Innovative use of intracranial EEG: The authors take advantage of the high temporal and spatial resolution of iEEG to provide insight into fast-timescale dynamics in amygdala–hippocampal communication, which are not accessible via non-invasive methods.

2. Insight into emotional learning: The frequency-specific findings—particularly the bidirectional and valence-dependent modulation of theta sub-bands—represent a significant advance in our understanding of the neurophysiological basis of affective flexibility in humans.

3. Strong analytical framework: The combination of power analyses, phase connectivity, transfer entropy, and DCM provides a comprehensive and convergent account of the data. Model selection is appropriately used to support directional inferences.

4. Translational relevance: By isolating oscillatory mechanisms specific to aversive learning and extinction, the work lays conceptual groundwork for spectral-targeted neuromodulation in disorders such as PTSD.

Limitations and areas for clarification:

1) Generalizability of findings: As acknowledged by the authors, participants were epilepsy patients undergoing clinical monitoring. While the authors excluded epileptogenic zones and controlled for noise, this sample limits the generalizability of findings. Further justification or contextualization of how these results may translate to neurotypical populations would strengthen the manuscript.

Response: We thank the reviewer for the insightful comment.

Introduction Pg 4

"Intracranial electroencephalography (iEEG) in neurosurgical patients offers a unique opportunity to bridge this gap, providing high temporal and spatial resolution to investigate the spectral signatures and directionality of amygdala-hippocampal interactions during emotional learning. Although such invasive data provide opportunities for fundamental neuroscience their generalizability may be limited, as such recordings are only feasible in clinical populations."

Limitation Pg 10

“This study has several limitations. First, our data come from patients with epilepsy which may limit generalisability. However, we adopted strict screening to only include electrode contacts not diagnosed as epileptogenic zones for our analyses. This limitation must be weighed against the unique strengths of iEEG, which provides direct recordings of neural population activity that are not accessible with non-invasive techniques such as functional MRI (fMRI) which measures slow hemodynamic response (~5 seconds) while EEG/MEG are constrained by poor spatial localization, particularly in deeper brain structures such a s amygdala and hippocampus.”

2) Anatomical specificity: The inability to resolve subnuclei within the amygdala and hippocampus is a limitation. If possible, anatomical localization of electrodes could be better visualized (e.g., in supplemental figures), and the potential impact of heterogeneous subregions further discussed.

Response: We have now edited Figure 1 with magnified localisation of electrode contacts. We fully agree that dissociating the respective contributions of the basolateral amygdala (BLA) and central amygdala (CeA) would be highly informative, given their well-established roles in fear learning and fear expression, respectively. However, we take extreme caution in doing so, for several methodological reasons listed below. First, although depth electrode contacts can be localised to the amygdala at the macroanatomical level with high confidence, the spatial resolution of standard clinical iEEG electrodes limits unambiguous assignment to specific subnuclei. The human amygdala contains small, densely packed, and interdigitated nuclei, and even with subnuclear masks, contact localisation remains uncertain. In our dataset, the inter-contact spacing in the electrodes is 1.5 mm, while the spatial spread of local field potentials is estimated to be ~2–3 mm, meaning that individual contacts likely sample activity from multiple neighbouring nuclei (Kreiman et al., 2006; Kajikawa et al., 2017).

Second, the oscillatory measures used here—particularly phase-based and directional metrics—are especially sensitive to volume conduction and field spread. While bipolar re-referencing can mitigate volume conduction, it can also introduce phase distortions or reversals and suppress genuine neuronal interactions by subtracting shared signals (Arnulfo et al., 2015; Shirhatti et al., 2016). Under these conditions, attempting to assign effects to specific subnuclei risks producing spurious or misleading results.

Finally, although very limited studies have employed subnuclear segmentation approaches, their interpretability depends critically on electrode density, contact spacing, and balanced sampling across nuclei. With only 53 contacts located within the amygdala will result in insufficient and uneven coverage of putative subnuclei for statistically robust comparisons.

For these reasons, and to avoid over-interpretation, we conservatively report effects at the level of the amygdala as a whole and now clarify this rationale more explicitly in the Methods and Limitations. We fully agree that subnuclear dissociation is an important direction for the field and highlight future work using higher-density electrodes (e.g., Neuropixels-like designs

with <1 mm spacing), larger datasets with balanced subnuclear coverage, and convergent imaging or histological validation to directly test differential BLA versus CeA contributions to oscillatory dynamics during fear learning and expression.

We have now added these remarks in the limitation's section Pg 10

"Amygdala and hippocampus are composed of multiple nuclei and thus precise functionality of each subnuclei cannot be differentiated with these recordings. Subnuclear dissociation would be an important direction for the field and highlight future work using higher-density electrodes (e.g., Neuropixels-like designs with <1 mm spacing), larger datasets with balanced subnuclear coverage, and convergent imaging or histological validation to directly test differential basolateral amygdala (BLA) versus central amygdala (CeA) contributions to oscillatory dynamics during fear learning and expression."

3) Stimulus potency: The use of IAPS images as unconditioned stimuli may not fully capture the intensity of aversive experiences. While this choice has advantages in ecological validity and widespread applicability, it may limit the engagement of certain fear circuits. This tradeoff could be more explicitly discussed.

Response: We acknowledge that IAPS images, while providing ecological validity and standardized presentation, may elicit milder aversive responses compared to primary unconditioned stimuli such as electric shocks, and thus may not fully engage all fear-related circuits, particularly those mediating intense autonomic or defensive responses. We have added sentences to explicitly discuss this limitation, clarifying that our results primarily reflect cortical–medial temporal dynamics associated with learned anticipatory and extinction processes, rather than maximal fear expression

Pg 11

"Finally, we acknowledge that IAPS images, while providing ecological validity and standardized presentation, may elicit milder aversive responses compared to primary unconditioned stimuli such as electric shocks, and thus may not fully engage all fear-related circuits, particularly those mediating intense autonomic or defensive responses."

4) Definition of theta sub-bands: The division of the theta range into low (3–5 Hz) and high (6–8 Hz) sub-bands is central to the findings. It would be helpful to clarify whether this division aligned with prior human and maybe rodent literature, and support it with data-driven assessments resulting from the analysis of raw data from actual behavioral epochs.

Response: We thank the reviewer for this insightful comment. The subdivision of the theta range into low (3–5 Hz) and high (6–8 Hz) sub-bands is supported by prior animal and human

literature indicating functional heterogeneity within theta oscillations. In rodents, slower theta components (~3–6 Hz) have been associated with affective and motivational processes, whereas faster theta (>6 Hz) is more strongly linked to locomotion and hippocampal-dependent memory functions (Kramis et al., 1975; McFarland et al., 1975; Buzsáki, 2002). Converging evidence in humans from intracranial EEG and MEG studies similarly supports dissociable roles for low- and high-theta activity, with lower theta frequencies implicated in emotional and interoceptive processing and higher theta frequencies in memory and cognitive control (Jacobs, 2014; Giraud & Poeppel, 2012), including recent human iEEG evidence demonstrating functionally distinct hippocampal theta sub-bands (Goyal et al., 2020).

Importantly, this subdivision was further supported by a data-driven approach: weighted phase-lag index analyses conducted on task-relevant behavioural epochs revealed distinct frequency-specific coupling within the theta range, motivating the separation into low- and high-theta sub-bands. Directed connectivity analyses (transfer entropy and dynamic causal modelling) were subsequently applied to these empirically defined frequency ranges.

Introduction Pg 11

"Oscillatory activity in the theta band (3–8 Hz) has emerged as a key candidate mediating amygdala-hippocampal interactions, with distinct sub-bands within the theta range potentially supporting different phases of learning (Herweg, Solomon et al. 2020). For instance, rodent studies have identified a frequency-specific dissociation, wherein lower theta frequencies (<6 Hz) dominate during aversive learning, while higher theta (>6 Hz) frequencies are associated with extinction and safety learning (Lesting, Narayanan et al. 2011). These findings suggest a frequency-specific reconfiguration of network dynamics that tracks the transition from threat acquisition to safety learning. Additionally functional heterogeneity within theta band, is supported by animal studies (Kramis, Vanderwolf et al. 1975) and converging human EEG including iEEG evidence demonstrating dissociable roles of low (3-6Hz)- and high-theta activity (>6Hz) (Lega, Jacobs et al. 2012, Goyal, Miller et al. 2020, Grossberg 2021). However, direct evidence for such frequency-resolved mechanisms in human amygdala-hippocampal coupling is limited and lacking in learning paradigms, owing to the spatial and temporal constraints of non-invasive neuroimaging. Nevertheless, related intracranial EEG studies have reported frequency-specific connectivity, including theta–alpha coupling between the hippocampus and entorhinal cortex during memory (Li, Cao et al. 2024), and theta-band amygdala–prefrontal interactions (Chen, Tan et al. 2021)."

5) Participant-level variability: Given the small sample size, it would be informative to report how consistent the observed effects were across participants. Any individual variability, lateralization effects, or correlations with behavioral measures (e.g., valence ratings) would enrich interpretation.

Response: We thank the reviewer for highlighting participant-level variability. Inter-individual variability in affective experience was instead incorporated directly by relating amygdala theta ERSP to each participant's US valence ratings. Our directional connectivity approach transfer entropy and its statistical analyses was based on within-subject differences between conditions thus accounting for potential variability. Furthermore, in or DCM analyses, Bayesian model selection was performed with random effects to taken into account participant variability. Electrode implantation is clinically determined, leading to heterogeneous and often unilateral amygdala–hippocampal coverage, which substantially reduces power for participant-level or lateralization analyses and risks misleading inference.

Limitations Pg 11

"Intracranial EEG studies are necessarily constrained by small and clinically heterogeneous samples, and subgroup, laterality or covariate analyses for sex or age would be statistically underpowered. Larger sample sizes with harmonised datasets can investigate this in future as well as to establish robust amygdala-hippocampal connectivity profile."

Minor comments:

6) The methods could benefit from a more detailed description of trial structure and temporal alignment between stimuli and signal epochs.

Response: We have added this information in the methods section

Methods Pg 13

"Illustration of the task paradigm is shown in Fig 1 B. During the task, patients were instructed to view the stimuli displayed on the screen. Two different shapes (triangle and circle) constituted the CS. CS+ was reinforced with negative or neutral valence images from International Affective Picture System (IAPS) (Lang and Bradley 2007) which acted as the US (US; contingency of 100%). 40 images each of negative and neutral category were selected. CS+ were presented 45 times each and were presented on a desktop screen 60cms away from the participant in random order. Intertrial interval consisting of a black fixation cross shown on a white background was jittered between 1000-1500ms. Each CS+ and US lasted for 2s. During the intertrial interval, a black fixation cross was shown on a white background. The US delivery occurred upon the termination of CS+ (i.e., delay conditioning). We did not undertake responses for establishment of a conditioned response as this is arguably independent of conscious contingency awareness (Baeyens, Eelen et al. 1990, Walther and Nagengast 2006). Further, we used 100% probability for reinforcing with USs. "

Extinction testing was conducted on the following day, approximately 24–30 hours after conditioning, as clinical constraints precluded testing at an exact 24-hour interval. During the extinction phase, only CS+ stimuli were presented for 2 seconds, and these were not followed

by either aversive or neutral unconditioned stimuli. The precise time-based control of sending trigger pulses for the paradigms was programmed with Psychtoolbox (Kleiner, Brainard et al. 2007) run on MATLAB version 2018b (The MathWorks, Inc., Natick, MA), interfaced via a parallel port. Due to logistical constraints and to avoid long experimental sessions, inherent to conducting intracranial EEG studies in clinical settings, we did not acquire any explicit behavioural measures of conditioning and extinction."

7) The discussion would be strengthened by further integration with human neuroimaging findings (e.g., fMRI studies of extinction networks).

Response: We have added a paragraph in the discussion section. Pg 10

"Our findings align closely with human fMRI work demonstrating that fear conditioning and extinction rely on coordinated amygdala–hippocampal–prefrontal networks. fMRI studies have shown dynamic changes in functional coupling between the amygdala and hippocampus across conditioning and extinction, alongside increased engagement of medial prefrontal regions during extinction and extinction recall (Milad and Rauch 2007, Sehlmeyer, Schöning et al. 2009). More broadly, human neuroimaging consistently implicates reconfiguration of this network during suppression and updating of aversive memories. The present iEEG results extend these findings by revealing the spectral and directional mechanisms—specifically theta sub-band–dependent bidirectional interactions—that underlie these large-scale fMRI connectivity patterns at a finer temporal scale."

8) Clarify whether sex or age differences were assessed or controlled for, as these may influence emotional memory processing.

Response: We thank the reviewer for this important point. Although sex and age can influence emotional memory processing in healthy populations, we did not incorporate this into our analyses was not designed or powered to assess these effects. Intracranial EEG studies are necessarily constrained by small and clinically heterogeneous samples, and subgroup or covariate analyses for sex or age would be statistically underpowered. Importantly, our primary inferences are based on group contrasts (conditioning vs. extinction) potentially negating demographic variability. We have now explicitly stated this in the limitations section and note that future studies with larger, harmonised intracranial datasets or complementary non-invasive cohorts will be better suited to explicitly address age- and sex-related modulation of emotional memory circuitry.

Limitations Pg 11

"We did not incorporate sex and age into our analyses which have been shown to influence emotional memory processing. Intracranial EEG studies are necessarily constrained by small

and clinically heterogeneous samples, and subgroup, laterality or covariate analyses for sex or age would be statistically underpowered. Larger sample sizes with harmonised datasets can investigate this in future as well as to establish robust amygdala-hippocampal connectivity profile."

Summary:

This is an important and well-executed stud that makes a valuable contribution to our understanding of the neural dynamics underlying emotional learning and extinction. While some limitations remain—particularly regarding population generalizability and anatomical resolution—the overall data quality, analytical rigor, and conceptual clarity merit publication.

Response: We thank the reviewer for positively appraising our work.

Reviewer #3 (Remarks to the Author):

The manuscript titled 'Adaptive shifts in amygdala–hippocampal theta coupling govern aversive learning and extinction' addresses an important topic in the field. The study investigates the spectral and directional dynamics of amygdala–hippocampal interactions during aversive learning and extinction paradigm in humans. Using intracranial EEG (iEEG) from patients undergoing clinical monitoring for epilepsy, the authors conducted a two-day conditioning-extinction task with emotionally valenced stimuli. They identified frequency-specific changes in power and connectivity, notably distinguishing low (3–5 Hz) and high (6–8 Hz) theta sub-bands and their dynamic directionality. The results suggest a bidirectional and frequency-specific interplay between the amygdala and hippocampus underpinning emotional memory regulation.

Overall, the study leverages intracranial recordings, offering high temporal and spatial resolution not available in non-invasive modalities. This is a rare and valuable dataset in human neuroscience. The authors employ a comprehensive suite of analyses including ERSPs, wPLI, Transfer Entropy, Dynamic Causal Modelling (DCM), and phase-amplitude coupling (PAC), providing a multifaceted characterization of connectivity. The double dissociation in low and high theta band connectivity, with corresponding directional shifts, offers a novel insight into the dynamic regulation of emotional learning and extinction. The correlation of amygdalar theta activity with valence ratings adds interpretability, linking neural findings to subjective emotional appraisal. The discussion links the findings well with existing literature in both human and animal models, advancing the conceptual framework around emotional memory and extinction.

While the manuscript presents important and novel findings, major revisions are necessary to address limitations in generalisability, clarify methodological choices, and improve the

presentation of results and figures. If these issues are addressed, the work could make a significant contribution to the field of human affective neuroscience.

Major Concerns

1) While the authors acknowledge this, the epilepsy patient population may exhibit atypical neurodynamics, even when excluding epileptogenic zones. Future studies in healthy individuals (e.g., via MEG or advanced fMRI) are needed to replicate findings.

Response: We thank the reviewer for the insightful comment. We have remarked about this in the introduction and limitations sections

Introduction Pg 4

"Intracranial electroencephalography (iEEG) in neurosurgical patients offers a unique opportunity to bridge this gap, providing high temporal and spatial resolution to investigate the spectral signatures and directionality of amygdala-hippocampal interactions during emotional learning. Although such invasive data provide opportunities for fundamental neuroscience their generalizability may be limited, as such recordings are only feasible in clinical populations."

Limitation Pg 10, 11

"This study has several limitations. First, our data come from patients with epilepsy which may limit generalisability. However, we adopted strict screening to only include electrode contacts not diagnosed as epileptogenic zones for our analyses. This limitation must be weighed against the unique strengths of iEEG, which provides direct recordings of neural population activity that are not accessible with non-invasive techniques such as functional MRI (fMRI) which measures slow hemodynamic response (~5 seconds) while EEG/MEG are constrained by poor spatial localization, particularly in deeper brain structures such a s amygdala and hippocampus."

2) No explicit behavioral indices of learning were collected during the conditioning/extinction phases, which limits interpretation of learning success on a trial-by-trial basis.

Response: We thank the reviewer for this comment. We agree that the absence of explicit behavioural indices during the conditioning and extinction phases limits the ability to assess learning success on a trial-by-trial basis and to directly relate neural dynamics to behavioural performance. This limitation reflects logistical constraints inherent to conducting intracranial EEG studies in clinical settings, which required the conditioning procedure to be completed within 12–15 minutes and extinction within approximately 10 minutes. Incorporating

behavioural ratings would have added an additional 4–5 minutes to each phase, which was not feasible within these constraints.

This has been mentioned in the methods sections as well as limitations section

Methods paradigm section Pg 13

"Due to logistical constraints and to avoid long experimental sessions, inherent to conducting intracranial EEG studies in clinical settings, we did not acquire any explicit behavioural measures of conditioning and extinction."

Limitations Pg 11

"In this study, no explicit behavioural indices of learning were collected during the conditioning/extinction phases which limits investigations of learning outcomes."

3) The use of IAPS images, while ecologically valid, may produce less robust conditioning than electric shock or airpuff paradigms. This could affect the magnitude and specificity of neural responses.

Response: We acknowledge that conditioning paradigms using primary reinforcers such as electric shock or airpuff can yield stronger and more rapid associative learning effects than visual stimuli alone. However, the use of IAPS images was a deliberate methodological choice to enhance ecological validity and to avoid introducing additional somatosensory stimulation that could confound limbic and hippocampal neural responses. A substantial body of prior work demonstrates that emotionally salient visual stimuli, including IAPS images, reliably support fear and affective learning and robustly engage amygdala–hippocampal circuitry (Phelps et al., 2001; LaBar & Cabeza, 2006; Lang et al., 2008). Notably, amygdala activation during conditioning has been shown to occur even in the absence of physical aversive reinforcement when stimuli are emotionally meaningful (Morris et al., 1998; Phan et al., 2004). Consistent with these findings, we observed condition-specific neural responses within the amygdala–hippocampal network, indicating that the paradigm was sufficient to induce associative processing. Nevertheless, we now explicitly acknowledge in the revised manuscript that the use of visual affective stimuli may limit the overall magnitude of conditioning effects relative to paradigms employing primary reinforcers.

Limitations Pg 11

"Finally, we acknowledge that IAPS images, while providing ecological validity and standardized presentation, may elicit milder aversive responses compared to primary unconditioned stimuli such as electric shocks, and thus may not fully engage all fear-related circuits, particularly those mediating intense autonomic or defensive responses. No explicit

behavioural indices of learning were collected during the conditioning/extinction phases which limits learning outcomes."

4) Although 30 patients were included, only 14 had simultaneous amygdala-hippocampus recordings, limiting some connectivity analyses' robustness.

Response: We agree that only a subset of participants (14 out of 30) had simultaneous amygdala–hippocampus recordings, reflecting the clinical constraints of electrode implantation in intracranial epilepsy monitoring. Connectivity analyses were therefore necessarily restricted to this subgroup. Importantly, an iEEG sample of 14 participants with simultaneous recordings is comparable to, and in many cases larger than, sample sizes used in prior intracranial connectivity studies (Sonkusare, Nguyen et al. 2020, Chen, Tan et al. 2021, Li, Cao et al. 2024), where access to specific regional pairs is inherently limited by clinical considerations. We have however, added a sentence in the limitations section. Pg 11

"Intracranial EEG studies are necessarily constrained by small and clinically heterogeneous samples, and subgroup, laterality or covariate analyses for sex or age would be statistically underpowered. Larger sample sizes with harmonised datasets can investigate this in future as well as to establish robust amygdala-hippocampal connectivity profile."

Minor Issues:

5) Many grammatical and typographical errors should be corrected. Here are some examples:

We have revised the overall text and corrected grammatical and typographical errors.

6) Figure 1:

Panel A: The color-coded dots are very small and difficult to visualize. Improvements are necessary, as the current presentation may not be clear or meaningful to readers. Additionally, the two plots in the middle and on the right—representing the amygdala and hippocampus—are confusing without more detailed descriptions in the figure legend. If the two plots represent different subjects, using separate color bars for each plot would make interpretation easier for readers.

Response: We have edited the figure to make it larger for clear visualisation and have edited the caption as well.

7) Line 132: Please define the abbreviation 'ERSP' at its first occurrence in the text.

Response: This has now been defined

Results section Pg 4

"Amygdala Event related spectral perturbations (ERSPs)"

8) Lines 132–153: Is there any reason to use unmatched closing parentheses (i.e., missing opening parentheses)? The presentation of the statistical results appears unusual due to the use of unmatched closing parentheses.

Response: An additional closed parenthesis was a mistake which has now been removed.

9) Figure 2 is of poor quality in terms of text font size, statistical plots, and figure legends, making it difficult to effectively convey the experimental results.

Response: This figure has been edited for clarity and the caption has also been revised

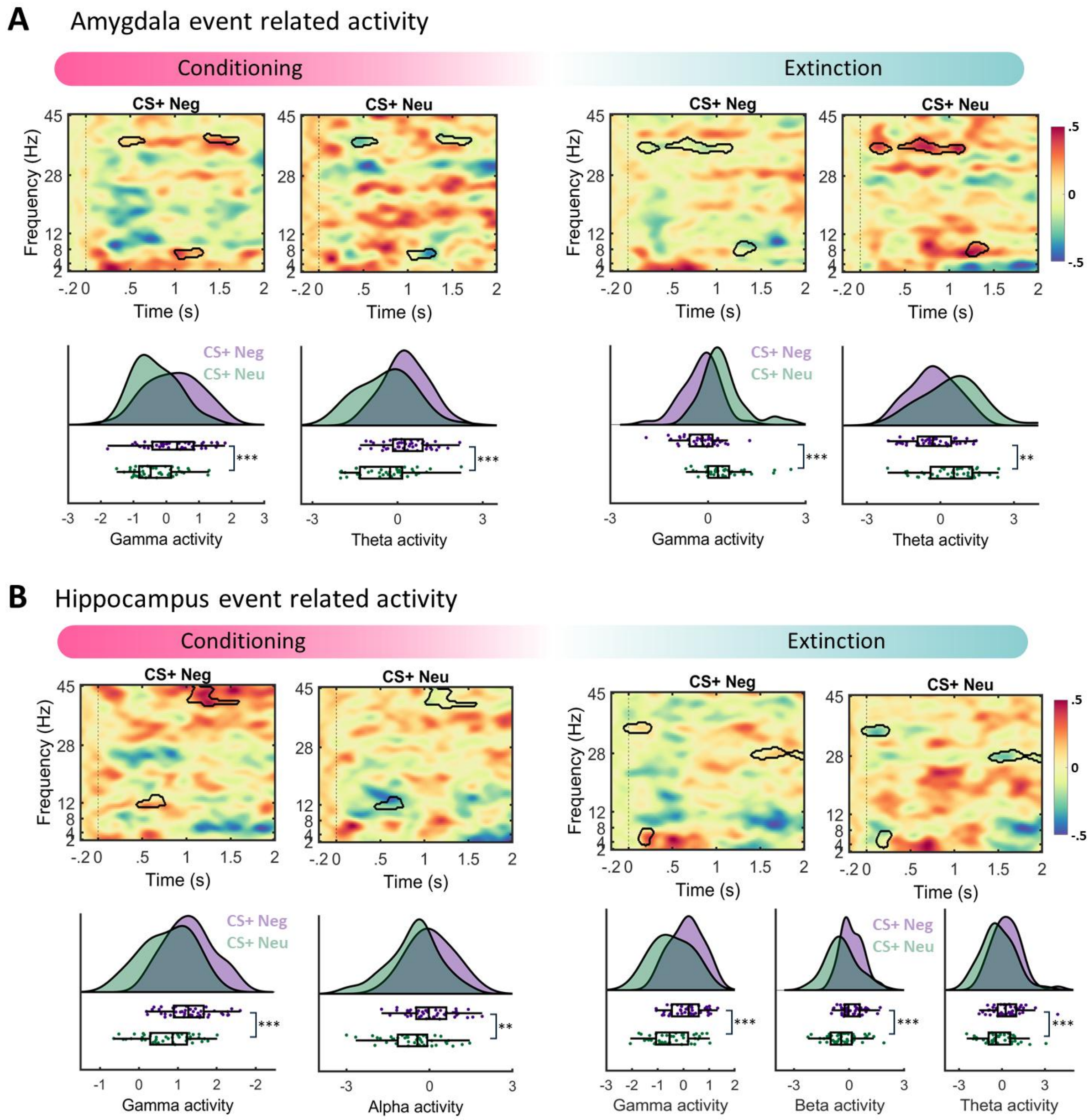

Figure 2. Task induced responses and condition differences. ***A)*** *Amygdala:* Grand-averaged event-related spectral perturbation (ERSP) maps during conditioning (left) and extinction (right) for CS+ Negative (CS+ Neg) and CS+ Neutral (CS+ Neu) stimuli. Warmer colours indicate task-related increases in spectral power, whereas cooler colours indicate power decreases relative to baseline. Black contours denote time–frequency clusters showing significant differences between CS+ Neg and CS+ Neu conditions, identified using permutation testing. Raincloud plots below the ERSP maps summarize mean spectral power extracted from the significant clusters. Purple distributions represent CS+ Neg trials and green distributions represent CS+ Neu trials. Y-axis in these raincloud plots depict the distribution density and has no independent interpretive scale. Notably, theta- and gamma-band responses show a reversal in relative activity during extinction compared with conditioning. ***B)*** *Hippocampus:* Similar grand averaged ERSP maps as for amygdala during conditioning (left) and extinction (right). Raincloud plots below the ERSP maps summarize mean spectral power extracted from the significant clusters. Purple distributions represent CS+ Neg trials and green distributions represent CS+ Neu trials. Y-axis in these raincloud plots depict the distribution density and has no independent interpretive scale.

Result section:

10) The first part of the Results section, which includes 'Amygdala ERSPs' and 'Hippocampal ERSPs', is not well organized and contains some confusing subheadings.

Response: Done. Subheading has been edited. Pg 4, 5

"Amygdala Event related spectral perturbations (ERSPs)

Hippocampal ERSPs"

11) Line 146: Fig. B top: missing figure number

Figure 4 appears to be missing, or incorrect figure numbers may have been used for Figures 5 and 6.

Response: We apologise for the incorrect figure number. This has now been corrected.

12) Line 207 (also line 213): Incorrect figure citation for wPLI — "(Fig. 5B left)" should be "(Fig. 5A, left)." The panel B presented Transfer Entropy (TE). However, the figure number itself also appears to be incorrect.

Response: We apologise for the incorrect figure number. This has now been corrected.

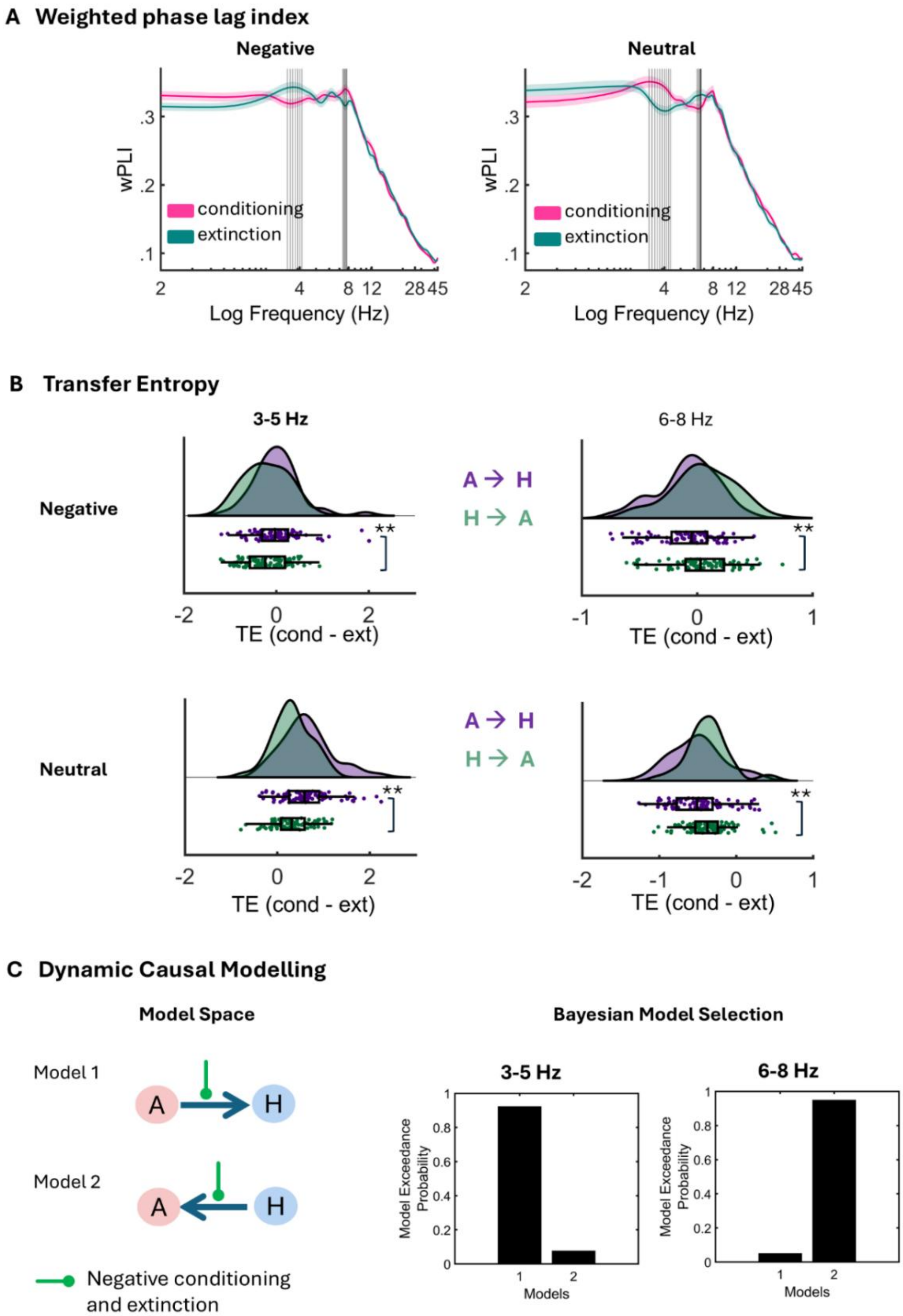

Figure 4: Amygdala-hippocampal connectivity. ***A)*** *Weighted phase lag index (wPLI):* connectivity differences in low theta (3-5 HZ) and high theta (6-8 Hz) were observed. Specifically, a double dissociation was found with lower low-theta and high theta connectivity in negative and neutral conditioning and extinction. Grey vertical lines show frequencies where significant differences were found with permutation testing (see methods). Shading indicates SEM ***B)*** *Transfer entropy (TE):* Directional information flow between the amygdala and hippocampus was quantified using transfer entropy in the low theta (3–5 Hz; left) and high theta (6–8 Hz; right) frequency bands during conditioning (cond) and extinction (ext) for CS+ Negative (CS+ Neg) and CS+ Neutral (CS+ Neu) stimuli. TE was computed for the amygdala → hippocampus and hippocampus → amygdala directions. Condition differences were calculated as CS+ Neg in conditioning minus CS+ Neg in extinction and CS+

Neu in conditioning minus CS+ Neu in extinction, in for each direction and shown as raincloud plots. Y-axis in these raincloud plots depict the distribution density and has no independent interpretive scale. Statistical analyses revealed predominant amygdala-to-hippocampus information flow in the low-theta range, whereas hippocampus-to-amygdala information flow dominated in the high-theta range, for both negative and neutral conditions. ***C)*** *Dynamic Causal Modelling:* For testing the effective connectivity in both 3-5Hz and 6-8Hz we employed a model space which comprised two models, with unidirectional connection from amygdala (A) to hippocampus (H) and from hippocampus to amygdala respectively and CS+ Negative conditioning and extinction task modulation effects influencing the connectivity. Bayesian model selection identified model 1 (amygdala to hippocampus) as possessing the highest exceedance probability (92.3%) for 3-5 Hz while for 6-8 Hz model 2 (hippocampus to amygdala) had the highest exceedance probability (94.9%).

13) Figure 5A: I am wondering whether the data traces were smoothed, as this could significantly alter the observed patterns of wPLI changes.

Response: The wPLI were not smoothed.

14) Figure 5B: In the top portion of each plot, the y-axis should be labeled to indicate whether it represents amplitude or an index value. Additionally, abbreviations such as 'cond' and 'ext' should be defined in the figure legend to ensure the figure is self-explanatory.

Response: We have now edited the figure and the caption for clarity. In the raincloud plots we are using, the y-axis does not represent a measured quantity in the way a typical bar chart or scatter plot does; rather, it shows the density of the distribution (i.e., the shape of the cloud is a smoothed estimate of the probability distribution of the x-values). This is the same convention used in the example referenced in the paper introducing raincloud plots (Allen, Poggiali et al. 2021), where the vertical axis simply spreads the density for visual clarity and does not carry a standalone unit or interpretable scale. We chose this format for exactly that reason: the focus is on comparing the distributions along the x-axis, not on the absolute values of the y-axis. For clarity, we have added a note in the caption that the y-axis depicts the distribution density and has no independent interpretive scale.

"y-axis depicts the distribution density and has no independent interpretive scale"

16) Figure 6: Legends.

More clarity is needed when using terms such as negative, neutral, conditioning, and extinction, whether used individually or in combination. For example, the sentence 'Bottom row shows the PAC for phase of amygdala modulating amplitude of hippocampus activity contrasts between negative (bottom left) and neutral (bottom right) conditioning and extinction' is quite confusing and should be rewritten for better understanding.

The figure should include brief statistical results, along with appropriate labeling and descriptions for each part. The text "Phase Amplitude Coupling" in the top-left corner of the

figure is unclear—it's not evident whether it refers to the left column specifically or the entire figure. This should be revised for clarity or removed altogether.

Response: We have now revised the figure and the caption for clarity.

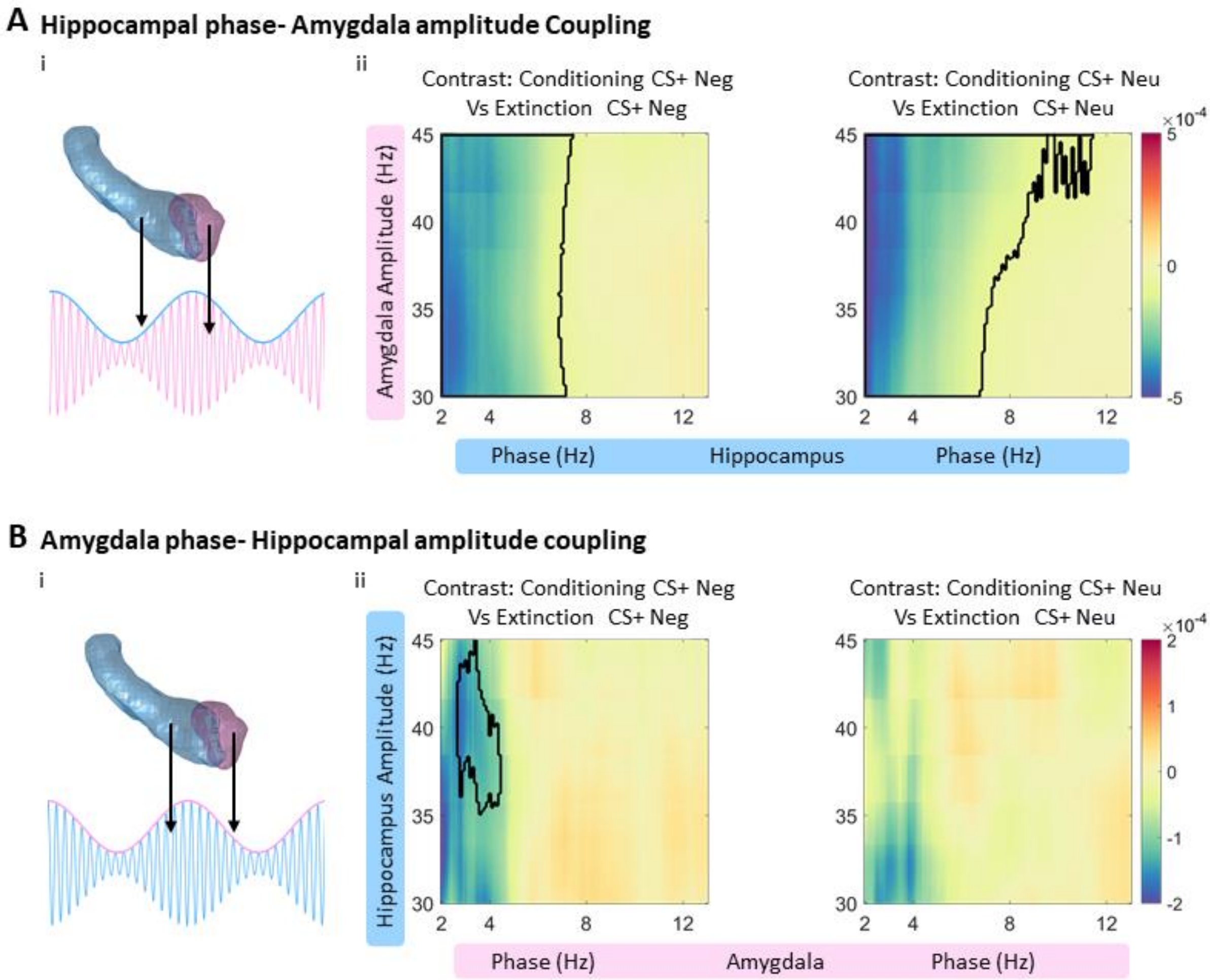

Figure 5: Phase–amplitude coupling (PAC) between the hippocampus and amygdala. ***A)*** *Hippocampal phase–amygdala amplitude coupling* (i) illustration of hippocampal low frequency phase shown in blue and amygdala frequency amplitude shown in pink ii) PAC comodulograms contrast between conditioning and extinction for CS+ Negative (left) and CS+ Neutral (right) stimuli. Black outlines denote clusters that reached statistically significant difference between the two conditions based on permutation testing. ***B)*** *Amygdala phase– Hippocampal amplitude coupling* (i) illustration of amygdala low frequency (2-12 Hz) phase shown in pink and hippocampal frequency amplitude (30-45 Hz) shown in blue ii) PAC comodulograms contrast between conditioning and extinction for CS+ Negative (left) and CS+ Neutral (right) stimuli. Black outlines denote clusters that reached statistically significant difference between the two conditions based on permutation testing (see methods).

17) "This approach allows robust, model -free quantification of dynamic, nonlinear interactions between brain regions and has been widely validated in both EEG and intracranial recordings Wollstadt, P., et al. (2014) Wibral, M., Vicente, R., & Lindner, M. (2014)." Line 502: incorrect reference citation style

Response: We have corrected this.

"This approach allows robust, model-free quantification of dynamic, nonlinear interactions between brain regions and has been widely validated in both EEG and intracranial recordings (Wollstadt, Martínez-Zarzuela et al. 2014)."

Reviewer #4 (Remarks to the Author):

The present study investigated the neural circuit mechanisms underlying learning and extinction in the human brain. The present work observed increases in theta and gamma band activities in the amygdala during conditioning for emotionally arousing stimuli (IAPS images as the US) and subsequent reversal of these spectral patterns during extinction. Furthermore, the results suggest frequency-specific oscillatory dynamics within the amygdala-hippocampal circuit during emotional conditioning and extinction. More specifically, low theta dynamics dominated amygdala-hippocampal connectivity during learning, while high theta dynamics dominated hippocampal-amygdala connectivity during extinction. These results provide novel evidence that distinct frequency bands support adaptive emotional behavior. The manuscript is well written and clear. It was co-reviewed by a principal investigator and a postdoctoral fellow. We offer suggestions for areas where the paper can be improved below.

Introduction

1) The authors do an excellent job of describing the background literature relevant to their study, but do not lay out their specific hypotheses based on this prior literature. More information on this study's specific hypotheses would be helpful.

Response: We have now added sentences about the hypotheses in the introduction section Pg 4

"We anticipated task induced condition differences in the low frequency range (2-12 Hz) especially with rapid onset latency for emotion valence paradigms, based on our previous amygdala neural recordings (Sonkusare, Qiong et al. 2023). We also expected for theta connectivity based on recent findings of hippocampal theta sub bands (Goyal, Miller et al. 2020). On an exploratory basis, we also characterised interactions across various frequencies termed "cross frequency coupling (CFC)", speculated to play a crucial role in the coordination of complex cortical computation (Jensen and Colgin 2007)."

Methods

2) It would be helpful to know more details about the amygdala localizations. Prior work suggests the BLA is essential for the initial learning and consolidation of fear memories. However, the central nucleus is critical for orchestrating the expression of the fear response. Are the authors able to isolate the different contributions of the BLA vs. the CeA to the oscillatory responses? The authors mention in the limitations sections that functionality of each subnuclei cannot be differentiated with these recordings, however, many labs have used

subnuclei segmentations in intracranial epilepsy patients to determine the contributions of different nuclei to different cognitive processes.

Response: We thank the reviewer for this insightful and important comment. We fully agree that dissociating the respective contributions of the basolateral amygdala (BLA) and central amygdala (CeA) would be highly informative, given their well-established roles in fear learning and fear expression, respectively. However, we take extreme caution in not pursuing these analyses, for several methodological reasons listed below. Although depth electrode contacts can be localised to the amygdala at the macroanatomical level with high confidence, the spatial resolution of standard clinical iEEG electrodes limits unambiguous assignment to specific subnuclei. The human amygdala contains small, densely packed, and interdigitated nuclei, and even with subnuclear masks, contact localisation remains uncertain. In our dataset, the inter-contact spacing in the electrodes is 1.5 mm, while the spatial spread of local field potentials is estimated to be ~2–3 mm, meaning that individual contacts likely sample activity from multiple neighbouring nuclei (Kreiman, Hung et al. 2006, Kajikawa, Smiley et al. 2017, Ray 2023). Under these conditions, attempting to assign effects to specific subnuclei risks producing spurious or misleading results. Finally, although very limited studies have employed subnuclear segmentation approaches, their interpretability depends critically on electrode density, contact spacing, and balanced sampling across nuclei. With only 53 contacts located within the amygdala will result in insufficient and uneven coverage of putative subnuclei for statistically robust comparisons.

For these reasons, and to avoid over-interpretation, we conservatively report effects at the level of the amygdala as a whole and now clarify this rationale more explicitly in the Methods and Limitations. We fully agree that subnuclear dissociation is an important direction for the field and highlight future work using higher-density electrodes (e.g., Neuropixels-like designs with <1 mm spacing), larger datasets with balanced subnuclear coverage, and convergent imaging or histological validation to directly test differential BLA versus CeA contributions to oscillatory dynamics during fear learning and expression.

Methods, Data Acquisition Pg 12

"In our dataset, the inter-contact spacing in the electrodes is 1.5 mm, while the spatial spread of local field potentials is estimated to be ~2–3 mm, meaning that individual contacts likely sample activity from multiple neighbouring nuclei (Kreiman, Hung et al. 2006, Kajikawa, Smiley et al. 2017, Ray 2023). Therefore, we did not undertake subnuclear localisation of amygdala and hippocampus which may risk producing spurious or misleading results."

Limitations section Pg 11

"Subnuclear dissociation would be an important direction for the field and highlight future work using higher-density electrodes (e.g., Neuropixels-like designs with <1 mm spacing), larger datasets with balanced subnuclear coverage, and convergent imaging or histological

validation to directly test differential BLA versus CeA contributions to oscillatory dynamics during fear learning and expression."

3) Was the Extinction procedure always conducted the subsequent day after Conditioning? Or did the delay vary for some patients? It would be great to list this somewhere.

Response: We thank the reviewer for the insightful comment. The Extinction procedure always conducted the subsequent day after Conditioning with 24-30 hours. Precise 24 hour separation is difficult due to logistical constraints of clinical settings. We have added this information in the methods section

Methods Pg 13

"Extinction testing was conducted on the following day, approximately 24–30 hours after conditioning, as clinical constraints precluded testing at an exact 24-hour interval. During the extinction phase, only CS+ stimuli were presented for 2 seconds, and these were not followed by either aversive or neutral unconditioned stimuli."

4) The Paradigm section in the Methods says a triangle and a square were used for the CS. However, Figure 1 depicts a triangle and a circle.

Response: We apologise for the oversight on our part. The stimulus was indeed a circle. This has been corrected

Figure 1 CAption

"Two different shapes (triangle and circle) constituted the CS. CS+ was reinforced with negative or neutral valence images from International Affective Picture System (IAPS) (Lang and Bradley 2007) which acted as the US (US; contingency of 100%)."

5) The Paradigm section in the Methods says the intertrial interval consisted of a white fixation cross on a black background, however, in Figure 1 there is a black fixation cross on a white background.

Response: We apologise for another oversight. The figure is now corrected. There is a black fixation cross on a white background. We have corrected this information in the main text Methods paradigm section. Pg 13

"Intertrial interval consisting of a black fixation cross shown on a white background were jittered between 1000-1500ms"

6) The Paradigm section in the Methods doesn't detail the procedure used for the Extinction phase of the task.

Response: This information has now been added. Pg 13

"Extinction testing was conducted on the following day, approximately 24–30 hours after conditioning, as clinical constraints precluded testing at an exact 24-hour interval. During the extinction phase, only CS+ stimuli were presented for 2 seconds, and these were not followed by either aversive or neutral unconditioned stimuli."

7) For the dynamic causal modelling/phase coupling analyses, it would be helpful to know whether ipsilateral amygdala-hippocampus interactions were analyzed or contralateral in some cases. Studies suggest some subregions of the amygdala (like the BLA) only project to the ipsilateral hippocampus, so it would be unclear how one would interpret phase coupling interactions between the amygdala and the contralateral hippocampus, for example.

Response: We thank the reviewer for this insightful comment. All connectivity analyses, including dynamic causal modelling and phase-coupling measures, were performed exclusively on ipsilateral amygdala–hippocampus pairs. No contralateral amygdala–hippocampus interactions were analysed, in recognition of known anatomical constraints as the reviewer has pointed out. In two participants who had bilateral depth electrode coverage of both the amygdala and hippocampus, analyses were restricted to hemisphere with greater number of contacts. In these cases, we selected the hemisphere with the greater number of contacts to maximise signal reliability and statistical power while maintaining anatomical consistency across subjects.

We have now clarified this selection criterion and the restriction to ipsilateral connections in the Methods section. Pg 14

"No contralateral amygdala–hippocampus interactions were analysed, in recognition of known anatomical constraints as the reviewer has pointed out. In two participants who had bilateral depth electrode coverage of both the amygdala and hippocampus, analyses were restricted to hemisphere with greater number of contacts."

Results

8) You show greater amygdala theta and gamma activity to negative compared to neutral CS during the learning/conditioning phase, however, what does this theta/gamma activity look like across the 45 conditioning trials. Is this a pattern that only emerges towards the end of the learning? Or is it present from the first few trials?

Similarly, for the extinction phase you see this trend reverse (reduced amygdala theta/gamma activity to negative CS). Does it take several trials for this reversal to occur? Is it most apparent towards the end of the 60 extinction trials?

Response: Our analyses was based on grand averaged time frequency conditioning differences. In the present dataset, we are unable to reliably characterise the evolution of amygdala theta and gamma activity across the full sequence of the 45 conditioning trials or

the 60 extinction trials. This limitation arises because trials were visually inspected and excluded on a subject-by-subject basis for artefacts and interictal epileptiform activity, resulting in a variable number and distribution of retained trials across participants. Consequently, early versus late trial averages are not matched across subjects, precluding a robust and interpretable analysis of learning-related temporal dynamics.

To avoid introducing bias or over-interpreting uneven trial sampling, we therefore focused our analyses on condition-wise contrasts (negative vs neutral CS) pooled across the conditioning phase, rather than modelling trial-by-trial learning trajectories. We agree that examining how amygdala oscillatory responses evolve over learning is an important question for future work. Addressing this will require datasets with higher trial retention, automated artefact rejection, and task designs optimised for longitudinal modelling of learning-related neural dynamics.

10) Are these effects unique to the amygdala/hippocampus? Do you see this reversal of activity between conditioning and extinction in other regions that we know are important for these kinds of tasks? i.e. ACC or prefrontal cortex?

Response: We thank the reviewer for the insightful comment.

We appreciate the reviewer's thoughtful suggestion and fully agree that prefrontal regions such as the ACC and OFC play an important role in aversive learning and extinction. However, the primary aim of the present study was to provide a mechanistic, frequency- and direction-specific characterisation of amygdala–hippocampal interactions, given the rarity of simultaneous intracranial recordings from both structures in humans. Expanding the analyses to prefrontal regions would substantially broaden the scope of the paper. Importantly, prefrontal electrode coverage in these patients is heterogeneous and not uniformly available across participants, limiting statistical power and comparability across regions. For these reasons, we chose to restrict the analyses to the amygdala–hippocampal circuit to maintain anatomical specificity and conceptual clarity. We now explicitly acknowledge this as a limitation and outline planned future work that will systematically examine prefrontal contributions (including ACC and OFC) and their interactions with the MTL during learning and extinction, which is being addressed in a dedicated follow-up study.

Limitations Pg 11

"Prefrontal contributions (including ACC and OFC) and their interactions with the amygdala and the hippocampus during learning and extinction is also critical which should be systematically examined in future."

11) Are these amygdala and hippocampal ERSP effects during conditioning unique to the CS? Or do they carry over to the US (IAPS stimuli presentation period)? i.e. in the instances where

you see greater amygdala theta and gamma activity to the negative CS compared to the neutral CS, do you also see greater amygdala theta and gamma activity to the subsequent US?

Response: We thank the reviewer for this thoughtful question. In the present design, ERSP analyses were intentionally time-locked to the conditioned stimulus (CS), which allowed us to quantify learning-related changes relative to a well-defined pre-CS (fixation cross) baseline. In contrast, the unconditioned stimulus (US; IAPS images) was not preceded by a comparable baseline period, and its presentation followed immediately after the CS, making it difficult to disentangle US-evoked activity from residual CS-related and anticipatory responses. As a result, a direct and statistically valid comparison of theta and gamma activity during the US period is not feasible with the current data.

12) Lines 145-153 detailing the hippocampal ERSPs results note the same effect for learning and extinction (greater alpha and gamma activity to negative CS). However, the heading for this section mentions a shift in activity from learning to extinction involving theta, implying there is a difference in what is happening during learning vs. extinction. There is no mention of theta in the written results for the extinction phase here.

Response: This has now been corrected. Pg 5

"The hippocampus showed greater alpha and gamma activity to negative compared to neutral CS+ (Fig. B top): gamma – CS+ negative: mean = 0.30, sd = 0.61), CS+ neutral: mean = -0.25, sd = 0.65, t_{45} = 4.33, P_{FDR} = 0.00008); alpha – CS+ negative: mean = 0.06, sd = 0.82; CS+ neutral: mean = -0.55, sd = 0.94, t_{45} = 3.16, P_{FDR} = 0.002. During extinction increased activity to negative compared to neutral CS+ in the theta and gamma range was observed (Fig. 2B bottom): gamma – extinction negative: mean = 0.13, sd = 0.62), neutral: mean = -0.45, sd = 0.77, t_{45} = 3.87, P_{FDR} = 0.0003; beta negative: mean = 0.16, sd = 0.60, extinction neutral: mean = -0.39, sd = 0.87, t_{45} = 4.06, P_{FDR} = 0.0001; theta – negative: mean = 0.33, sd = 1.02), neutral: mean = -0.27, sd = 1.11), t_{42} = −3.17, P_{FDR} = 0.0006."

Figures

13) The Figure 1 caption doesn't include any information about the Extinction procedure that's depicted.

Response: This information has been added in the caption and also in the methods section of paradigm

*"**Figure 1. Localization of electrode contacts and task paradigm. A)** Amygdala shown in pink and hippocampus shown in blue in standard MNI brain space (L=left side brain, R=right side brain). Each dot is an electrode contact colour coded corresponding to each patient (left). Amygdala electrode contacts shown in blue and hippocampus contacts shown in red (right) **B)** Conditioning and extinction task paradigm: The paradigm was conducted across two*

successive days in a clinical setting, with conditioning (pink) undertaken on Day 1 and extinction (teal) undertaken on Day 2. Conditioned stimuli (CS+) consisted of a triangle or a circle presented for 2000 ms, followed by presentation of the unconditioned stimulus (US) for 2000 ms. The triangle was paired with a negative US, comprising negatively valenced images, whereas the circle was paired with a neutral US, comprising neutrally valenced images selected from the International Affective Picture System (IAPS). Extinction procedure was carried on Day 2 which followed similar process as conditioning except negative and neutral images were not shown. A fixation cross preceded each CS presentation and was jittered between 1000–1500 ms. "

Methods section Pg 13

"Extinction testing was conducted on the following day, approximately 24–30 hours after conditioning, as clinical constraints precluded testing at an exact 24-hour interval. During the extinction phase, only CS+ stimuli were presented for 2 seconds, and these were not followed by either aversive or neutral unconditioned stimuli."

14) The Figure 3A caption doesn't match what is stated in the text. The figure legend says a greater difference between neutral and negative valence is associated with smaller theta activity. The text says a larger neutral-negative valence difference is associated with greater theta power.

Response: We have now edited this for clearer understanding. We have also added sentences in the Results sections to clarify this analysis. Fig 3 caption

"The mean theta-band difference between CS+ Negative and CS+ Neutral significantly linked to US valence ratings (left), such that larger differences in neutral versus negative valence ratings were associated with more negative neutral versus negative theta activity (greater CS+ Neg theta activity)."

*"Amygdalar theta activity predicted the relative valence ratings i.e. participants who rated negative images as more aversive (i.e., had larger neutral–negative valence differences) showed greater theta power to CS+ negative stimuli compared to CS+ neutral ones (i.e., more negative y-values) (R^2 = .48, **p=.01) (Fig. 3A)."*

In the results section Pg 16

"For association of the mean of significant clusters in traditional frequency ranges from each patient with corresponding valence ratings, linear regression was employed with p significance at .01 after multiple comparisons corrections. Mean event-related activity for each frequency band cluster during the conditioning phase (Fig. 2) was computed separately for each participant. For those with multiple contacts in the region of interest, we averaged the significant cluster for those contacts. For both the amygdala and hippocampus, frequency specific condition differences were calculated as CS+ Neutral (Neu) minus CS+ Negative (Neg). This resulted in theta- and gamma-band difference measures for the amygdala, and alpha- and gamma-band difference measures for the hippocampus. These were associated with the

mean valence ratings of the negative and neutral unconditioned stimuli with Pearson's correlation corrected for multiple comparisons."

15) The Figure 3B caption mentions CS negative on top and CS neutral on bottom, but the figure itself depicts conditioning on the top and extinction on the bottom with no CS negative or CS neutral depictions. Is the red vs. blue trace in the HFBA plot the CS negative vs. neutral?

Response: We have added this legend in the figure and revised the caption for the figure for clearer understanding.

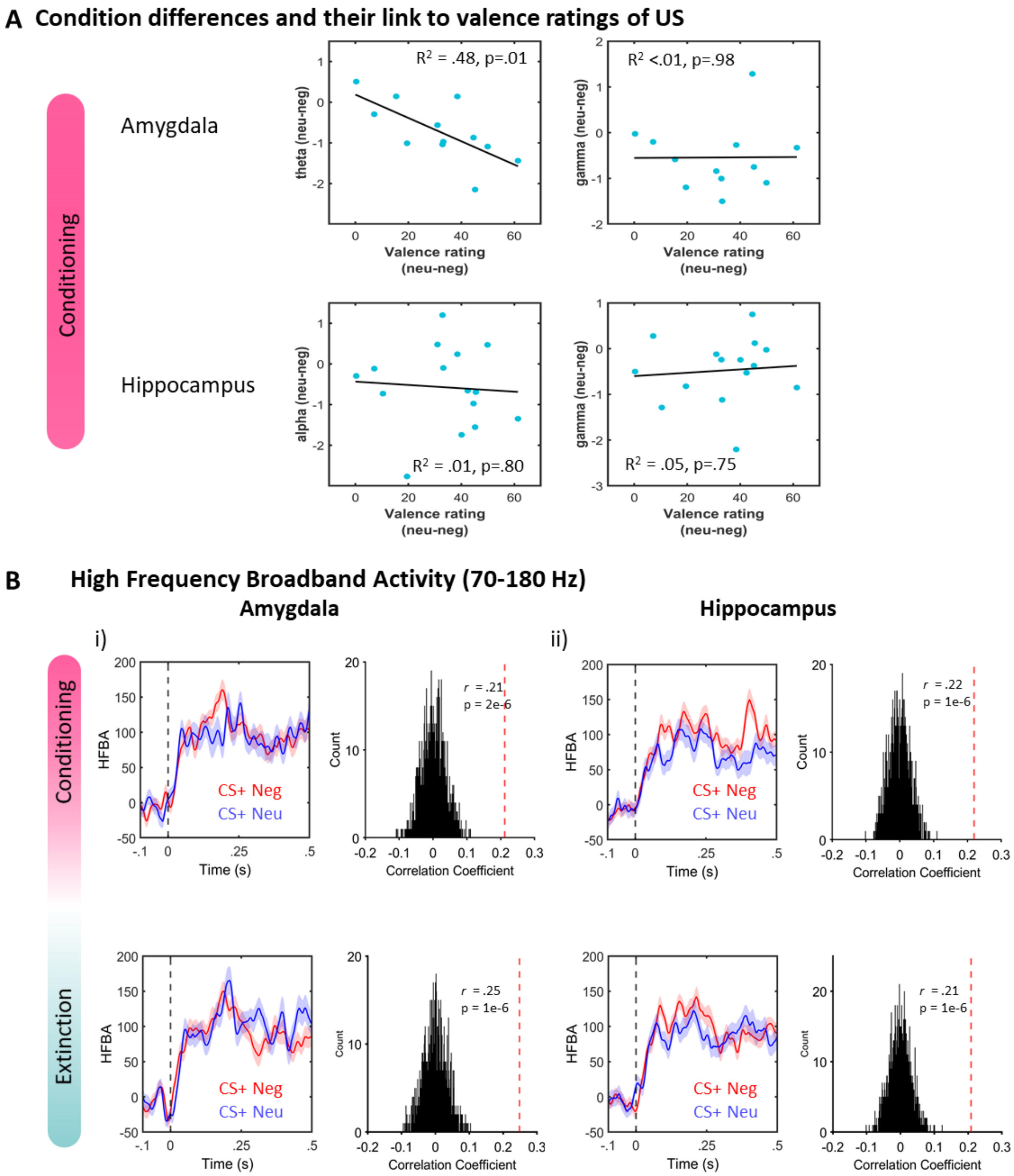

Figure 3: Event related condition differences during conditioning and their association with valence ratings of unconditioned stimuli and characterisation of high frequency activity profile. ***A)*** *Frequency-specific activity and valence ratings*: Mean event-related activity for each frequency band cluster during the conditioning phase (Fig. 2) was computed separately for each participant. For both the amygdala and hippocampus, frequency specific condition differences were calculated as CS+ Negative (Neg) minus CS+ Neutral (Neu). This resulted in theta- and gamma-band difference measures for the amygdala, and alpha- and gamma-band difference measures for the hippocampus. These were associated with the mean valence ratings of the negative and neutral unconditioned stimuli with Pearson's correlation corrected for multiple comparisons (see methods) (i) Amygdala: The mean theta-band difference between CS+ Negative and CS+ Neutral significantly linked to US valence ratings (left), such that larger differences in neutral versus negative valence ratings were associated with more negative neutral versus negative theta activity (greater CS+ Neg theta activity). In contrast, gamma-band activity did not show a significant association with valence ratings (right) (ii) Hippocampus: Neither alpha-band (left) nor gamma-band (right) activity differences showed a significant association with valence ratings. ***B)*** *High frequency broadband activity (HFBA)* (i) Amygdala: HFBA during the conditioning phase for negative and neutral images (top) and for extinction phase (bottom) showing high similarity with adjacent plot showing statistically testing with permutation testing showing null distribution and correlation value in dashed vertical line (ii) Hippocampus: HFBA during the conditioning phase for negative and neutral images (top) and for extinction phase (bottom) showing high similarity with adjacent plot showing statistically testing with permutation testing showing null distribution and correlation value in dashed vertical line.

16) In Figure 6, the PAC illustrations to the left imply that this PAC was observed in both the negative and neutral conditions. However, your study did not find PAC for phase of amygdala modulating amplitude of hippocampus in the neutral condition. I'm wondering if it may be clearer to only include these PAC pink and blue illustrations under the appropriate conditions where you observed significant PAC?

Response: We have now revised the figures and this in the manuscript and in the figures and we apologies that this wasn't clear in the methods section. Specifically, PAC analyses and conditioning differences were undertaken for both condition and extinction for both negative and neutral trials. The plots of each PAC co-modulogram is the contrast of the specific conditions now labelled for clarity. Contrasts are shown to summarise the results instead of showing 8 separate comodulgram plots.

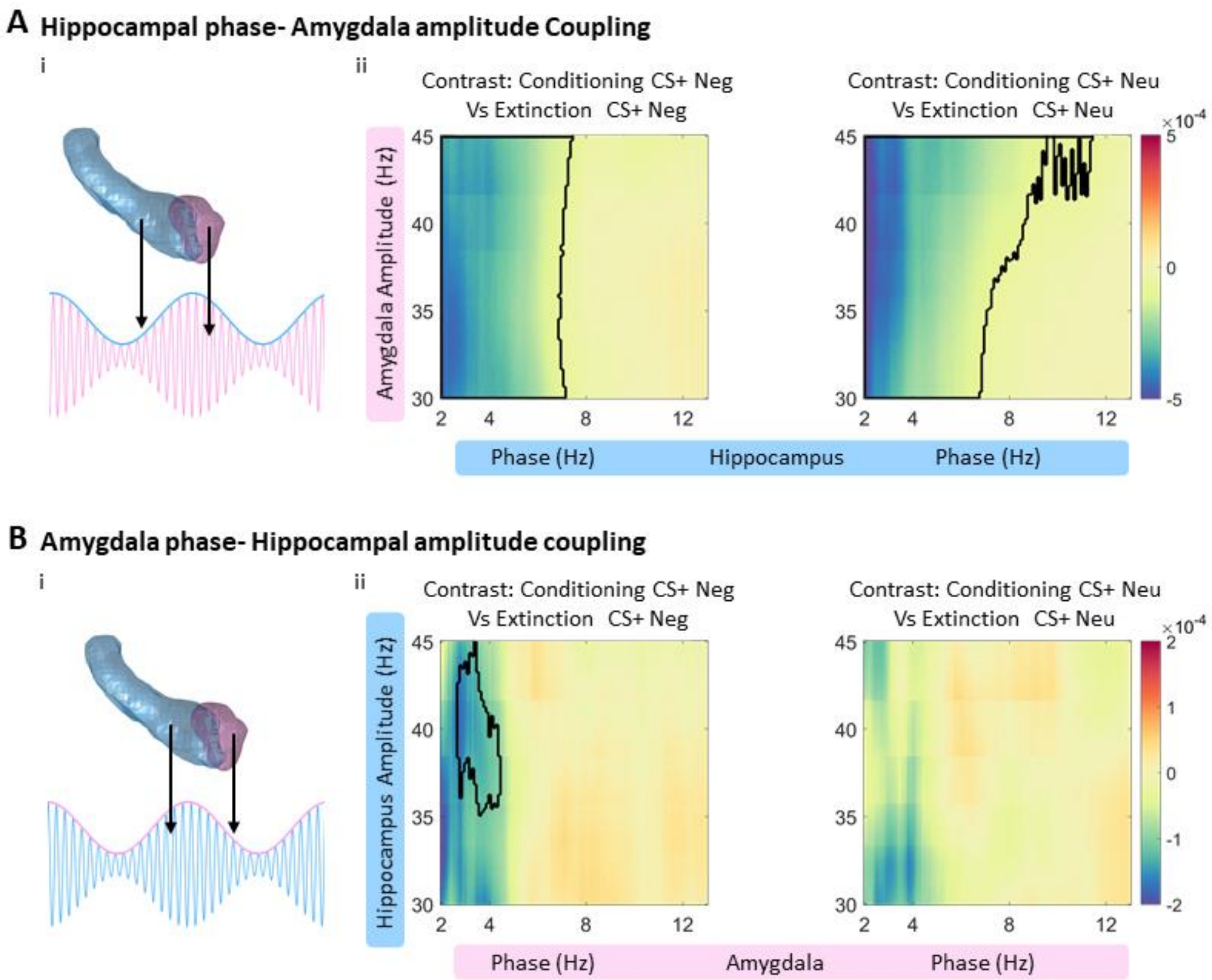

Figure 5: Phase–amplitude coupling (PAC) between the hippocampus and amygdala. ***A) Hippocampal phase–amygdala amplitude coupling*** (i) illustration of hippocampal low frequency phase shown in blue and amygdala frequency amplitude shown in pink ii) PAC comodulograms contrast between conditioning and extinction for CS+ Negative (left) and CS+ Neutral (right) stimuli. Black outlines denote clusters that reached statistically significant difference between the two conditions based on permutation testing. ***B) Amygdala phase– Hippocampal amplitude coupling*** (i) illustration of amygdala low frequency (2-12 Hz) phase shown in pink and hippocampal frequency amplitude (30-45 Hz) shown in blue ii) PAC comodulograms contrast between conditioning and extinction for CS+ Negative (left) and CS+ Neutral (right) stimuli. Black outlines denote clusters that reached statistically significant difference between the two conditions based on permutation testing (see methods).

Discussion

17) Line 316 mentions theta oscillations in the amygdala are linked to the encoding of emotional salience, however the Seidenbecher 2003 citation listed focuses on theta during memory retrieval. Their focus was on analyzing theta in animals already conditioned.

Response: We have replaced that citation with an appropriate one. Specifically, Chen et al., (Chen, Tan et al. 2021) demonstrated changes in amygdala theta predicted associability (learning rate) about emotionally relevant stimuli.

"Specifically, theta oscillations in the amygdala are linked to the encoding of emotional salience (Chen, Tan et al. 2021)."

18) Line 319-320 mentions that theta oscillations encode learned emotional associations, not simply valence. However, can the authors be certain that the CS+ hasn't taken on negative valence properties over the course of conditioning? We would argue that theta oscillations could still simply be encoding valence because the CS+ has taken on properties of unpleasantness. In general, this sentence can be worded differently to reflect the possibility that theta oscillations encode both stimulus valence and emotional associations, without suggesting the present study can tease those things apart for certain.

Response: We thank the reviewer for the suggestion which we have now incorporated. Pg 8

"Of note, here, theta response to CS+ (not the response to emotional USs), predicted subjective valence rating of USs, suggesting that theta oscillations encode both stimulus valence and emotional associations."

19) Line 327 highlights the possibility that the amygdala is involved in extinction and the "unlearning" of the association. However, there is also evidence that extinction instead reflects the new learning of a new association rather than unlearning. It would be helpful to speak to this idea and how your findings involving the amygdala and hippocampus during conditioning vs. extinction reconcile these two schools of thought on whether extinction is reversal of learning/unlearning vs. learning a new association. (See this review from the Phelps group: https://pmc.ncbi.nlm.nih.gov/articles/PMC4598943/)

Response: We agree that extinction is better understood as new learning rather than unlearning. Our findings support this view by showing that both the amygdala and hippocampus remain actively engaged during conditioning and extinction, but with a frequency- and direction-specific reorganization of their interaction. Conditioning and extinction were each associated with changes in oscillatory power in both regions, alongside reversals in directional theta-band communication. Rather than reflecting suppression of amygdala involvement, extinction was characterized by altered hippocampal–amygdala coordination across theta sub-bands, consistent with the formation of a new memory that regulates fear expression without erasing the original association. We have added a few sentences in the discussion section. Pg 9

"Extinction is widely considered to reflect new learning rather than erasure of the original fear memory (Dunsmoor, Niv et al. 2015). Our results support this account by showing that both the amygdala and hippocampus remain engaged during conditioning and extinction, but with a reconfiguration of spectral power and directional connectivity. Conditioning and extinction were distinguished by frequency-specific shifts in theta-band communication and reversals in information flow between regions. This dynamic reorganization suggests that extinction arises

from new learning implemented through altered amygdala–hippocampal coordination, rather than simple unlearning of prior associations."

20) In lines 328-338 the authors discuss theta-gamma dynamics during early and late conditioning/extinction. It would be great if the authors could clarify whether this in early/late in the context of trials across time or early/late on a trial-averaged basis looking at early vs. late with respect to stimulus onset.

Response: We wanted to remark about the condition differences found time relative to stimulus presentation i.e., 0-2000 milliseconds. Specially, hippocampus showed condition differences between CS+ Neg and CS+ Neu during conditioning at around 1 seconds for alpha activity, whereas in extinction this was found at 250-300 milliseconds. We have clarified this in the text. Pg 8

"Furthermore, hippocampal dynamics exhibited a temporal shift (time relative to stimulus presentation i.e., 0-2000 milliseconds): during conditioning, late ~1s alpha and gamma activity predominated, whereas extinction was characterized by early (300ms) and increased theta and gamma activity."

21) Citation needed in line 358 for "amygdala's role in affective salience signals"

Response: We have added the citation.

"In negative and neutral conditioning and extinction, amygdala-led low-theta synchrony predominated, consistent with the amygdala's established role in initiating affective salience signals (Anderson and Phelps 2001)."

22) In lines 365-370 it could be clearer how the DCM effects differed between conditioning vs. extinction. The way this section is currently written it sounds like the authors are saying negative conditioning and extinction showed the same effects. Whereas in the results sections it's clear that these directionality and frequency shifts are different between conditioning and extinction.

Response: We have revised the text for clear understanding. Pg 10

"Dynamic causal modelling revealed that conditioning and extinction differentially modulated both the directionality and frequency of amygdala–hippocampal interactions. During conditioning, connectivity was best explained by a 3–5 Hz amygdala-to-hippocampus model, consistent of the amygdala influence during aversive learning. In contrast, during extinction, the winning model shifted to a 6–8 Hz hippocampus-to-amygdala direction, suggesting a reversal of information flow i.e., hippocampus to amygdala, suggesting contextual modulation during safety learning. Thus, although both frequency phases engaged amygdala–

hippocampal coupling, they were characterised by distinct frequency bands and opposing directions of effective connectivity, rather than reflecting the same underlying mechanism."

Limitations and conclusion

23) The authors need to be transparent about how the DCM analysis wasn't run for the neutral conditioning and extinction (due to computational demands), so these system flexibility shifts in frequency effects may not be unique to encoding threat and may also be apparent in the neutral condition.

Response: We have now undertaken DCM analyses for the neutral condition. This is reported in the supplementary materials. We found consistent results with amygdala to hippocampus model winning with 81.6% probability and ambiguous finding of hippocampus to amygdala influence in 6-8Hz with 61.2% probability.

Results Pg 7

For neutral conditioning and extinction Bayesian model selection identified model 1 (amygdala to hippocampus) as possessing the highest exceedance probability (82.7%) for 3-5 Hz while for 6-8 Hz model 2 (hippocampus to amygdala) had the highest exceedance probability (55%) (supplementary materials SFig1).

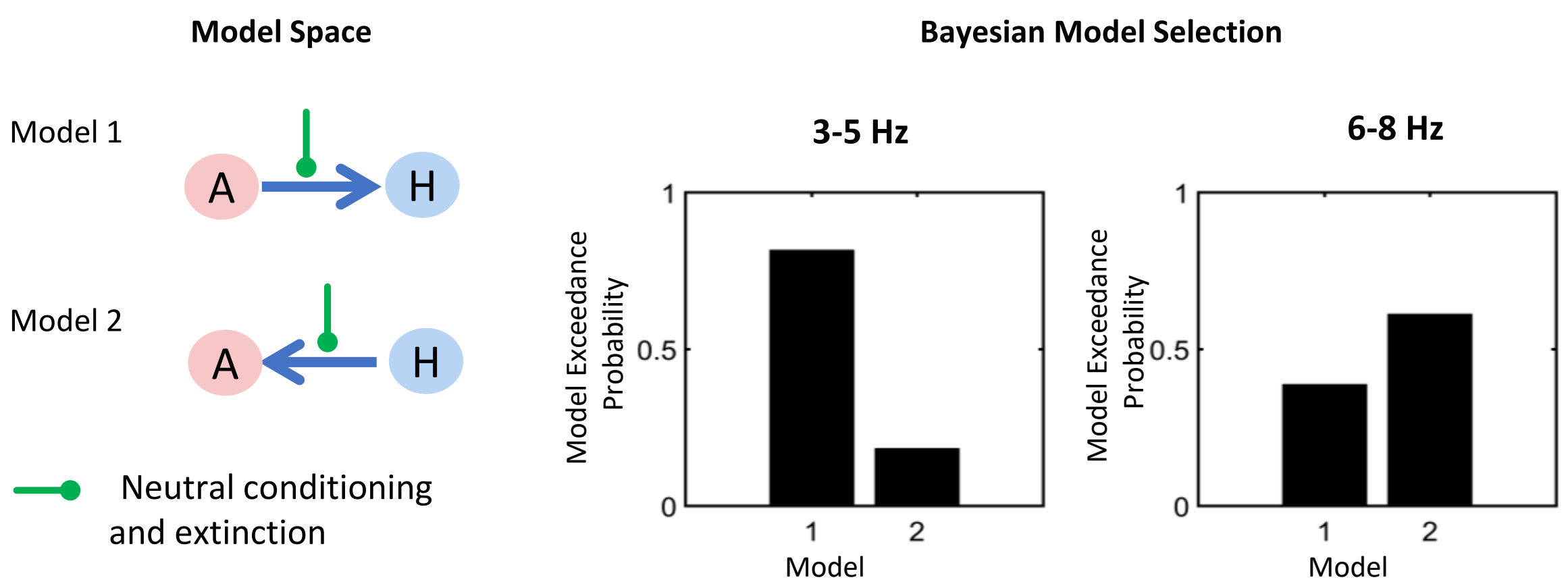

SFigure1: Dynamic Causal Modelling for neutral condition: Model space consisted of identical models shown in Figure 5 C except the experimental modulation was for CS+ Neutral in conditioning and extinction. Bayesian model selection identified model 1 (amygdala to hippocampus) as possessing the highest exceedance probability (81.6%) for 3-5 Hz while for 6-8 Hz model 2 (hippocampus to amygdala) had the highest exceedance probability (61.2%).

24) In line 399 the authors mention their study provides evidence of the mechanisms of how the brain "suppresses emotional memories". The suppression of emotional information is controversial in the learning and memory literature and the authors should reword this accordingly. (Something more appropriate would be "....we offer a mechanistic account of

how the brain flexibly encodes emotional information and subsequently updates emotional associations.”)

Response: We have reworded this as suggested Pg 11

“By identifying a double dissociation in low- and high-theta connectivity and revealing directional shifts in information flow across learning phases, we offer a mechanistic account of how the brain flexibly encodes and subsequently updates emotional associations.”

25) A brief analysis of prefrontal regions is within the scope of the present study. The authors have prefrontal coverage in these patients and highlight the role of prefrontal structures in this kind of learning/extinction. It would be helpful to know whether the observed effects are unique to the MTL or are also seen in regions like the ACC and OFC.

Response: We appreciate the reviewer’s thoughtful suggestion and agree that prefrontal regions such as the ACC and OFC play an important role in aversive learning and extinction. The primary aim of the present study was to provide a mechanistic, frequency- and direction-specific characterisation of amygdala–hippocampal interactions, motivated by past findings and the rarity of simultaneous intracranial recordings from both structures in humans. Expanding the analyses to prefrontal regions would substantially broaden the scope of the paper. Critically, amygdala and hippocampus are the most commonly implanted regions for this neurosurgical procedure. Prefrontal electrode coverage in these patients is heterogeneous and not uniformly available across participants, limiting statistical power and comparability across regions. For these reasons, we chose to restrict the analyses to the amygdala–hippocampal circuit to maintain anatomical specificity and conceptual clarity. We now explicitly acknowledge this as a limitation. We are however acquiring iEEG data with this paradigm with prefrontal coverage to specifically and systematically examine prefrontal contributions (including ACC and OFC) and their interactions with the MTL during learning and extinction, which is being addressed in a dedicated follow-up study.

Limitations Pg 11

“Prefrontal contributions (including anterior cingulate and orbito-frontal cortex) and their interactions with the amygdala and the hippocampus during learning and extinction is also critical which should be systematically examined in future.”

26) Future directions for how these novel findings might be used in the context of PTSD or other anxiety disorders would be helpful.

Response: We have added sentences remarking on the potential future use of our results. Pg 11, 12

“These findings enrich current models of affective flexibility and suggest new spectral targets for neuromodulation therapies in disorders characterized by impaired extinction learning, such as PTSD (Ressler and Mayberg 2007, Freire, Cabrera-Abreu et al. 2020). The observed

frequency-specific dissociation highlights oscillatory markers as potential biomarkers of treatment engagement or response, enabling patient stratification by symptom severity. Importantly, this study identifies two anatomical targets for neuromodulation. Theta burst stimulation (TBS; 5 Hz), currently FDA-approved for psychiatric conditions such as depression (Cohen, Bikson et al. 2021), could be adapted to target the amygdala or hippocampus in PTSD and anxiety disorders. Specifically, lower-theta inhibitory stimulation of the amygdala or higher-theta inhibitory stimulation of the hippocampus may modulate amygdala-hippocampal connectivity, offering a potential therapeutic approach for these disorders. Future work should investigate how these oscillatory dynamics interface with prefrontal regions and whether they can be harnessed to guide personalized interventions for affective dysregulation."

Reviewer #5 (Remarks to the Author):

I co-reviewed this manuscript with one of the reviewers who provided the listed reports. This is part of the Nature Communications initiative to facilitate training in peer review and to provide appropriate recognition for Early Career Researchers who co-review manuscripts.

References

Allen, M., et al. (2021). "Raincloud plots: a multi-platform tool for robust data visualization." Wellcome open research **4**: 63.

Anderson, A. K. and E. A. Phelps (2001). "Lesions of the human amygdala impair enhanced perception of emotionally salient events." Nature **411**(6835): 305-309.

Baeyens, F., et al. (1990). "Contingency awareness in evaluative conditioning: A case for unaware affective-evaluative learning." Cognition and emotion **4**(1): 3-18.

Benjamini, Y. and Y. Hochberg (1995). "Controlling the false discovery rate: a practical and powerful approach to multiple testing." Journal of the Royal statistical society: series B (Methodological) **57**(1): 289-300.

Bragin, A., et al. (1995). "Gamma (40-100 Hz) oscillation in the hippocampus of the behaving rat." Journal of Neuroscience **15**(1): 47-60.

Buzsáki, G. and J. J. Chrobak (1995). "Temporal structure in spatially organized neuronal ensembles: a role for interneuronal networks." Current opinion in neurobiology **5**(4): 504-510.

Chen, S., et al. (2021). "Theta oscillations synchronize human medial prefrontal cortex and amygdala during fear learning." Science advances **7**(34): eabf4198.

Cohen, S. L., et al. (2021). "A visual and narrative timeline of US FDA milestones for Transcranial Magnetic Stimulation (TMS) devices." Brain stimulation **15**(1): 73.

Delorme, A. and S. Makeig (2004). "EEGLAB: an open source toolbox for analysis of single-trial EEG dynamics including independent component analysis." Journal of neuroscience methods **134**(1): 9-21.

Dunsmoor, J. E., et al. (2015). "Rethinking extinction." Neuron **88**(1): 47-63.

Foster, B. L., et al. (2015). "Intrinsic and task-dependent coupling of neuronal population activity in human parietal cortex." Neuron **86**(2): 578-590.

Freire, R. C., et al. (2020). "Neurostimulation in anxiety disorders, post-traumatic stress disorder, and obsessive-compulsive disorder." Anxiety Disorders: Rethinking and Understanding Recent Discoveries: 331-346.

Goyal, A., et al. (2020). "Functionally distinct high and low theta oscillations in the human hippocampus." Nature communications **11**(1): 2469.

Grossberg, S. (2021). "A neural model of intrinsic and extrinsic hippocampal theta rhythms: Anatomy, neurophysiology, and function." Frontiers in Systems Neuroscience **15**: 665052.

Headley, D. B. and N. M. Weinberger (2013). "Fear conditioning enhances gamma oscillations and their entrainment of neurons representing the conditioned stimulus." Journal of Neuroscience **33**(13): 5705-5717.

Herweg, N. A., et al. (2020). "Theta oscillations in human memory." Trends in cognitive sciences **24**(3): 208-227.

Jensen, O. and L. L. Colgin (2007). "Cross-frequency coupling between neuronal oscillations." Trends in cognitive sciences **11**(7): 267-269.

Kajikawa, Y., et al. (2017). "Primary generators of visually evoked field potentials recorded in the macaque auditory cortex." Journal of Neuroscience **37**(42): 10139-10153.

Kleiner, M., et al. (2007). "What's new in Psychtoolbox-3?".

Kramis, R., et al. (1975). "Two types of hippocampal rhythmical slow activity in both the rabbit and the rat: relations to behavior and effects of atropine, diethyl ether, urethane, and pentobarbital." Experimental neurology **49**(1): 58-85.

Kreiman, G., et al. (2006). "Object selectivity of local field potentials and spikes in the macaque inferior temporal cortex." Neuron **49**(3): 433-445.

Lang, P. and M. M. Bradley (2007). "The International Affective Picture System (IAPS) in the study of emotion and attention." Handbook of emotion elicitation and assessment **29**: 70-73.

Lega, B. C., et al. (2012). "Human hippocampal theta oscillations and the formation of episodic memories." Hippocampus **22**(4): 748-761.

Lesting, J., et al. (2011). "Patterns of coupled theta activity in amygdala-hippocampal-prefrontal cortical circuits during fear extinction." PloS one **6**(6): e21714.

Li, J., et al. (2024). "Theta–Alpha Connectivity in the Hippocampal–Entorhinal Circuit Predicts Working Memory Load." Journal of Neuroscience **44**(4).

Milad, M. R. and S. L. Rauch (2007). "The role of the orbitofrontal cortex in anxiety disorders." Annals of the new York Academy of Sciences **1121**(1): 546-561.

Nichols, T. and S. Hayasaka (2003). "Controlling the familywise error rate in functional neuroimaging: a comparative review." Statistical methods in medical research **12**(5): 419-446.

Oostenveld, R., et al. (2011). "FieldTrip: open source software for advanced analysis of MEG, EEG, and invasive electrophysiological data." Computational intelligence and neuroscience **2011**(1): 156869.

Ray, S. (2023). How Do Local Field Potentials Measured with Microelectrodes Differ from iEEG Activity? Intracranial EEG: A Guide for Cognitive Neuroscientists, Springer**:** 273-282.

Ressler, K. J. and H. S. Mayberg (2007). "Targeting abnormal neural circuits in mood and anxiety disorders: from the laboratory to the clinic." Nature neuroscience **10**(9): 1116-1124.

Sehlmeyer, C., et al. (2009). "Human fear conditioning and extinction in neuroimaging: a systematic review." PloS one **4**(6): e5865.

Sonkusare, S., et al. (2022). "Power signatures of habenular neuronal signals in patients with bipolar or unipolar depressive disorders correlate with their disease severity." Translational psychiatry **12**(1): 72.

Sonkusare, S., et al. (2020). "Intracranial-EEG evidence for medial temporal pole driving amygdala activity induced by multi-modal emotional stimuli." Cortex **130**: 32-48.

Sonkusare, S., et al. (2023). "Frequency dependent emotion differentiation and directional coupling in amygdala, orbitofrontal and medial prefrontal cortex network with intracranial recordings." Molecular psychiatry **28**(4): 1636-1646.

Walther, E. and B. Nagengast (2006). "Evaluative conditioning and the awareness issue: assessing contingency awareness with the four-picture recognition test." Journal of Experimental Psychology: Animal Behavior Processes **32**(4): 454.

Wollstadt, P., et al. (2014). "Efficient transfer entropy analysis of non-stationary neural time series." PloS one **9**(7): e102833.

We thank the editor and reviewers' constructive suggestions and comments. Our responses are detailed below in dark blue. Relevant sections from the revised manuscript are quoted in italics with changes marked in red.

Reviewer #4 (Remarks to the Author):

The authors have done an excellent job addressing the concerns of the reviewers, but some additional minor revisions could be made.

Introduction
The authors now state hypotheses at the end of introduction section, but we think there needs to be some added clarity.

For example, they say "expected for theta connectivity based on our previous amygdala neural recordings". However, this is fairly vague. Did they expect strong connectivity between the amygdala and hippocampus?

They also state "anticipated task induced condition differences in the low frequency range...". But did they expect a difference between learning vs. extinction? If that's what they mean by "condition", then we think that can be more clearly stated.

Response: We have provided greater specificity in our hypotheses. We have revised the relevant passage to clarify both points:

Page 4

"We anticipated task-induced differences in the low frequency range (2–12 Hz) between conditioning (learning) and extinction phases, as well as between negative and neutral conditions within each phase, with rapid onset latencies for emotionally valenced stimuli, based on our previous amygdala neural recordings. We also expected stronger theta connectivity based on recent findings of hippocampal theta sub bands."

Methods
To our prior point on DCM modeling, the author's response to this comment is excellent. However, in the Methods section on page 14 the red text includes "...as the reviewer has pointed out." We think this phrase about the reviewer can be removed.

Response: We thank the reviewer for pointing this out. We have removed it.

Results
The authors have now run the DCM analysis for the neutral condition, however we think there needs to be some added clarity about the conclusions of this analysis. In the added red text in the Results on page 7 it's not clear what the conclusion is from this added analyses. A statement about how the results are different (or the same) between the DCM for the negative vs. neutral conditions would be helpful. Especially if the authors are trying to make the claim that there is something fundamentally different about the amygdala-hippocampus dynamics for negative vs. neutral condition.

Response: We have added a sentence to explain the DCM results.

Page 7

"*For the 3–5 Hz band, the winning model robustly indicated amygdala-to-hippocampus connectivity with a probability of 92.3%, whereas in the 6–8 Hz band, the hippocampus-to-amygdala model was favoured with a probability of 94.9%. (Fig 4C). In other words, the task (conditioning vs extinction) influences brain dynamics most clearly via amygdala's influence on the hippocampus in 3-5Hz and vice versa in 6-8Hz. For neutral conditioning and extinction Bayesian model selection identified model 1 (amygdala to hippocampus) as possessing the highest exceedance probability (82.7%) for 3-5 Hz while for 6-8 Hz model 2 (hippocampus to amygdala) had the highest exceedance probability (55%) (supplementary materials SFig1). These findings suggest that the directional motifs between amygdala and hippocampus are preserved across conditions but are robustly recruited only during negative emotional learning and extinction, which is consistent with emotional salience amplifying, rather than reconfiguring, amygdala–hippocampus theta dynamics."*
